# Supplementary material for: Richness and biogeography of Pteridoflora in montane forests of eastern Mexico
Source: PLoS One. 2026 May 7;21(5):e0346965. doi: 10.1371/journal.pone.0346965 (PMC13152183; doi:10.1371/journal.pone.0346965)
Supplement: Attachment — Submitted filename: PONE-D-25-44575_R1_rev.pdf [file pone.0346965.s004.pdf]

PLOS One

Richness and biogeography of Pteridoflora in montane forests of eastern Mexico

--Manuscript Draft--

**Manuscript Number:**	PONE-D-25-44575R1
**Article Type:**	Research Article
**Full Title:**	Richness and biogeography of Pteridoflora in montane forests of eastern Mexico
**Short Title:**	Mexican montane pteridoflora
**Corresponding Author:**	Isolda Luna-Vega<br>Universidad Nacional Autonoma de Mexico Facultad de Ciencias<br>mexico city, MEXICO
**Keywords:**	Conservation; diversity; ferns; floristics; lycophytes; montane environments
**Abstract:**	This study compiles and updates the checklist of ferns and lycophytes from the Sierra Madre Oriental (SMOR), Mexico. We analyzed the distribution and richness of these groups of seedless vascular plants, ensuring the accuracy and reliability of the data. We reviewed information on these taxa from regional floristic studies, field explorations, and herbarium specimens. Our updated list includes 567 species, of which 511 are ferns and 56 lycophytes, organized into 37 families and 137 genera. The five richest families are Pteridaceae (131), Polypodiaceae (76), Dryopteridaceae (68), Aspleniaceae (45), and Selaginellaceae (41). The five most diverse genera are Asplenium (42), Elaphoglossum (30), Pleopeltis (24), Myriopteris (22), and Selaginella (21). The Mexican states with the greatest species richness within the SMOR are Puebla (423 species), Hidalgo (322), Querétaro (227), and Tamaulipas (181). The five grid cells with the highest numbers, each containing more than 190 species, are situated in the southern part of the SMOR (Puebla and Veracruz). The physiographic provinces with the greatest diversity are Carso Huasteco and the Gran Sierra Plegada, with 523 and 175 species, respectively. Latitudinal analysis indicated low richness at the northern extreme (28 °- 29 ° N, 11 species) and high richness at the southern extreme (19 °- 20 ° N, 469 species), with a notable peak at 24 °- 25 ° N (350 species). The highest diversity within elevation intervals occurs at 1,000-1,500 m (455 species). The SMOR contains 22 species of the pteridoflora classified under various risk categories according to the Mexican Official Norm NOM-059-SEMARNAT-2010, including seven listed on the IUCN Red List and ten protected under CITES. The taxonomic diversity index shows that the SMOR is among Mexico's most species-rich mountain ranges. The species richness documented here in the SMOR accounts for 50-54% of the pteridoflora diversity recorded in Mexico, increasing to 59.3% based on recent floristic studies conducted in the region.
**Order of Authors:**	J. Daniel Tejero-Díez
	Perla V. Rodríguez-Sánchez
	Raúl Contreras-Medina
	Julio Cesar Ramírez Martínez
	Jonathan D. Amith
	Leccinum J. García-Morales
	Celia Sanginés-Franco
	Isolda Luna-Vega
**Response to Reviewers:**	Rebuttal<br>Reviewer #1: I have reviewed your manuscript titled "Richness and biogeography of Pteridoflora in montane forests of eastern Mexico" and found it to be well-written. The methods are appropriate, and the results and discussion are well presented. I have only two concerns:<br><br>1. You need to explicitly explain how you obtained the geographical coordinates for the specimens that lack them.

Powered by Editorial Manager® and ProduXion Manager® from Aries Systems Corporation

Author's response: For herbarium specimens without geographic coordinates, we used topographic maps at scale 1:50,000 produced by the Instituto Nacional de Estadística y Geografía (INEGI) and the previously published nomenclator of localities for the Sierra Madre Oriental (Ortiz-Bermúdez, 2004) to obtain the corresponding geographic coordinates. At the end, we tried to include only georeferenced and verified data in our study.

Ortiz-Bermúdez, E. (2004). Nomenclator de localidades. In: Biodiversidad de la Sierra Madre Oriental, Luna-Vega I., J.J. Morrone & D. Espinosa (eds.). pp. 25-62. Mexico City: UNAM-CONABIO.

2. The dataset used for all the analyses (species with their geographical coordinates) is not available. I encourage you to make this information public.
Author's response: the database is available through personal communication to the first author.

Reviewer #2: Methodologically, I think the article is well presented.
Although it is presented from a descriptive perspective of biodiversity, I believe that further exploration is needed to evaluate the reasons behind the results.

Spatial sampling bias:
1.The manuscript integrates data from literature, herbaria, and field collections; however, the potential spatial bias of sampling effort is not evaluated. It is recommended to include a sampling density map to visualize the spatial distribution of records.
Author's response: We have already created a sampling density map to show the spatial distribution of records. We chose not to include it in the first draft because of the manuscript's length. In this new version, we are adding the suggested map to Figure 2.
Reviewer 2: The referee suggested that the use of spatial rarefaction methods or effort-weighted corrections could improve the interpretation of results.
Author's response: As suggested by the referee, we undertook an effort-weighted sampling method for our manuscript. In our study using the Chao1 richness estimator, we identified 567 species. According to the estimator, the average estimated richness at this sampling level was about 670 species, with a 95% confidence interval of approximately 632-729 species. The difference between the lower and upper confidence limits is roughly 100 species, indicating moderate uncertainty around the estimate, as expected in communities with many rare or low-detectability taxa.
Comparing the observed richness (567 species) to the Chao1 mean estimate (≈670 species), our data falls short by about 100 species from the estimated total richness. This indicates that our sampling completeness is approximately 85%, which is fairly high, because Chao1 uses the number of very infrequently observed species to infer unseen diversity, it tends to produce higher richness estimates when rare species are relatively common in the sample (Chao, 1984; Chao, 1987).
Overall, these results suggest that although the sampling did not fully capture the total estimated richness, it still documented a significant portion of the community. To finish the task, we decided to create a new figure to compare our results with the Chao1 richness estimator (Fig. 6).

2. Details of sampling design:
Although field collections are mentioned, the manuscript does not provide information on sampling effort, the number of sites, or the spatial design employed. Providing these details would allow assessment of the representativeness of records and their potential influence on spatial patterns.
Author's response: Our study primarily relies on herbarium data and databases, so our field collection was minimal. Our database has been compiled and curated continuously by a Mexican specialist in ferns and lycophytes for over three decades. Therefore, we can confidently state that we have sufficient information on the spatial patterns of these taxa (see paragraphs above).
3. Data quality and geographic precision:
There is no evaluation of geographic precision or data quality. The manuscript does not describe how duplicate records, uncertain georeferences, or historical localities were handled. Ensuring data quality is essential to confirm that the generated maps reflect biological patterns rather than artifacts of incomplete or imprecise data.
Author's response: For vascular plants, species occurrence data typically come from

Powered by Editorial Manager® and ProduXion Manager® from Aries Systems Corporation

direct field observations or natural history specimens in herbaria (Daru, 2025), although specimens trump observations in representing global plant biogeographic patterns (Daru & Rodriguez, 2023). In general, no hard-and-fast rule exists for judging data quality in biogeographical analyses (Zizka et al. 2019). Indeed, collecting efforts are not randomly or regularly distributed, and biodiversity patterns are scale-dependent and sensitive to spatial resolution (James et al. 2018). Also, sampling designs were rarely quantified (Daru et al. 2018). In species occurrence records, problems with geographic location are a significant concern; particularly, erroneous or overly imprecise coordinates can bias biodiversity patterns at multiple spatial scales (Zizka et al. 2019).

As a result, data quality in online databases is a significant concern and has restricted their usefulness and reliability for research and conservation (Anderson et al. 2016). Herbarium specimens and their data have advantages that are verifiable, repeatable, sustainable, and persistent (James et al. 2018). In our paper, fortunately, most records in our database come from herbarium specimens revised directly by some of the authors, mainly the first author, a Mexican specialist in ferns and lycophytes. However, botanists have identified multiple biases in plant sampling from herbarium collections. For example, various analyses based on millions of herbarium and museum records have found spatial, temporal, taxonomic, functional trait, phylogenetic, and collector biases (Daru et al. 2018; Daru & Rodriguez 2023; Daru 2025). Limitations in our database were addressed and revised to minimize, primarily, spatial bias.

In our database, as is common with herbarium specimens, duplicate samples are found across different botanical collections. In our study, we removed duplicate records from the biogeographic analysis to ensure unique locations. Additionally, we identified and corrected records with switched latitude and longitude. We also excluded specimens with vague localities and inconsistencies. For herbarium specimens lacking geographic coordinates, we used topographic maps at a 1:50,000 scale from the Instituto Nacional de Estadística y Geografía (INEGI) and the previously published locality list for the Sierra Madre Oriental (Ortiz-Bermúdez, 2004) to obtain the corresponding coordinates. Ultimately, we aimed to include only georeferenced and verified data in our study.

Specimens stored in Natural History collections are available for long periods, sometimes up to 100 years, as in our study, because we have specimens collected in the 1880s. Unfortunately, collecting efforts are not evenly distributed over time and have declined since the mid-20th century (Daru et al. 2018; James et al. 2018). However, working with historical data presents challenges if there is no information about the species' locality or date. Additionally, the spatial location of the find may only be roughly known, or there could be issues with taxonomic naming (Lütolf et al. 2006). We examined all these issues in the historical records of ferns and lycophytes from the SMOR, and because of this, we did not include the 31 historical records from the 1800s.

Lütolf et al. (2006) highlight the use and importance of historical records, named 'ghost of past presence data', to support presence data in biogeographic studies. These records can be used as a strategy to verify a species' presence and reconfirm its presence where it was once observed. Also, these historical records are important because they can reveal changes in species distributions driven by anthropogenic activities or climate change (James et al., 2018; Franklin, 2023).

Anderson et al. (2016). Final report of the task group of GBIF data fitness for use in distribution modelling – Are species occurrence data in global online repositories fit for modelling species distributions? The case of the Global Biodiversity Information Facility (GBIF).

Daru, B. H. (2025). Tracking hidden dimensions of plant biogeography from herbaria. New Phytologist, 246(1), 61-77.

Daru, B. H., Rodriguez, J. (2023). Mass production of unvouchered records fails to represent global biodiversity patterns. Nature Ecology & Evolution, 7, 816-831.

Daru, B. H. et al. (2018). Widespread sampling biases in herbaria revealed from large-scale digitization. New Phytologist, 217, 939-955.

Franklin, J. (2023). Species distribution modelling supports the study of past, present and future biogeographies. Journal of Biogeography, 50, 1533-1545.

James et al. (2018). Herbarium data: Global biodiversity and societal botanical needs for novel research. Applications in Plant Science, 6(2), e1024.

Lütolf et al. (2006). The ghost of past species occurrence: improving species distribution models for presence-only data. Journal of Applied Ecology, 43, 802-815.

Ortiz-Bermúdez, E. (2004). Nomenclátor de localidades. In: Biodiversidad de la Sierra

Powered by Editorial Manager® and ProduXion Manager® from Aries Systems Corporation

Madre Oriental, Luna-Vega I., J.J. Morrone & D. Espinosa (eds.). pp. 25-62. Mexico City: UNAM-CONABIO.
Zizka et al. (2019). CoordinateCleaner: Standardized cleaning of occurrence records from biological collections databases. Methods in Ecology and Evolution, 10, 744-751.

4. Interpretation of maps and absence of records:
The manuscript assumes that the generated maps reflect biological patterns; however, it does not discuss whether absence of records corresponds to true species absence. It is suggested to clarify that some areas with no records may result from limited sampling and to consider the potential effect of this on interpreting richness and endemism hotspots.

Author's response: Fortunately, the first author of this study has been working with these organisms for more than 30 years. However, Dr. Tejero has not collected samples from all sites, so in some areas, the sampling of certain species may be incomplete.
'Presence-only' data are common sources of species distribution information, available through natural history collections like museum and herbarium collections (Lütolf et al. 2006). Absence data are rare or not easily accessible within herbarium datasets, and the absence of collections of a taxon at a specific location and time does not necessarily indicate its absence (James et al. 2018).
Mexico is a megadiverse country and, among ferns and lycophytes, is recognized as one of the Latin American countries with the highest diversity of these two plant groups (Almeida and Salino 2016). Large parts of the Neotropics remain inadequately sampled for vascular plants. The collections in this region are unevenly distributed, and many areas lack records of ferns and lycophytes (Almeida and Salino 2016). Mexico is no exception, and several regions, including the Sierra Madre Oriental, are still poorly sampled for these plants (Contreras-Medina et al. 2022). In our paper, we acknowledge this situation and state that "Some areas within the SMOR boundaries lack records of ferns or lycophytes, and 13 grid cells contain only one species. These low-diversity areas may be related to unexplored zones and insufficient sampling, as observed in many sectors of Mexico [68, 66] and the Neotropics [1, 104]."
5. Sensitivity to spatial scale:
The manuscript states that the 30′ × 30′ resolution provides reliable results, but no spatial sensitivity analysis is presented. It is recommended to recalculate richness, WE, and CWE metrics at different resolutions (e.g., 15′ and 60′) and compare patterns using rank correlations and hotspot overlap. This would allow evaluation of whether the identified richness and endemism centers are robust or dependent on the selected scale.

Author's response: In the original version, we explained the reason for choosing the 30 × 30 minutes latitude/longitude grid-cells (see page 9). "The spatial size of grid cells allows for a reliable spatial resolution of the distribution compared with other studies conducted with plants in the SMOR [18, 20, 27] regarding biodiversity data and biological conservation. We selected this scale because similar scales have been tested in previous studies on biogeography and the diversity of various biological groups in the Mexican biota [Rodríguez et al. 2018, Villaseñor et al, 2021], including ferns and lycophytes [Contreras-Medina et al., 2022]."
A 1° × 1° grid cell spatial scale has been previously used in biogeographic analyses in the SMOR, such as in the studies by Santa Anna del Conde et al. (2009) with Cactaceae and Villaseñor & Ortiz (2022) with vascular plants. We chose a finer spatial scale to obtain more precise patterns of fern and lycophyte distribution. In biogeographic analyses at the Mexican state level, a finer scale than ours has been applied in Hidalgo (Villaseñor et al. 2022) and Oaxaca (Contreras-Medina et al. 2022), using 15 × 15-minute and 20 × 20-minute grid cells, respectively.
We consider it unnecessary to recalculate the grid-cell richness at 15 and 60 degrees, nor the endemism centers. During the development of this study, we tested different grid cell sizes, as the referee suggested, but the 30x30 size seems most suitable because many spatial studies with other Mexican taxa use this size, which is appropriate for comparative results [Rodríguez et al. 2018, Villaseñor et al., 2021].
Villaseñor JL, Ortiz E. (2022). A phytogeographic assessment of the Sierra Madre Oriental physiographic province, Mexico. Botanical Sciences, 100(4), 1102–23.
Villaseñor JL, Ortiz E, Sánchez-González A. (2022). Riqueza y distribución de la flora vascular del estado de Hidalgo, México. Revista Mexicana de Biodiversidad, 93,

Powered by Editorial Manager® and ProduXion Manager® from Aries Systems Corporation

	e933920.<br>Santa Anna del Conde H, Contreras-Medina R, Luna-Vega I. (2009). Biogeographic analysis of endemic cacti of the Sierra Madre Oriental, Mexico. Biological Journal of the Linnean Society, 97(2), 373–89.<br><br>6. Conclusions<br>The article lacks proposals for the management, conservation, and restoration of vegetation and species in the study area. I believe that this document could serve as the basis for a series of public policies for the protection of the ecosystem.<br>Author's response: Indeed, we do not propose in this manuscript ideas about the management, conservation, and restoration of the vegetation of this mountain range. This task involves a different methodology than the one we are not using here. Our current objective is to know the spatial distribution of the taxa. Despite this, we have elaborated some maps of Mexican Natural protected areas that will be part of a subsequent study.
**Additional Information:**	
**Question**	**Response**
**Financial Disclosure**<br><br>Enter a financial disclosure statement that describes the sources of funding for the work included in this submission. Review the submission guidelines for detailed requirements. View published research articles from *PLOS ONE* for specific examples.<br><br>This statement is required for submission and **will appear in the published article** if the submission is accepted. Please make sure it is accurate.<br><br>**Funded studies**<br>Enter a statement with the following details:<br>• Initials of the authors who received each award<br>• Grant numbers awarded to each author<br>• The full name of each funder<br>• URL of each funder website<br>• Did the sponsors or funders play any role in the study design, data collection and analysis, decision to publish, or preparation of the manuscript?<br><br>Did you receive funding for this work?	Yes
Please add funding details.<br>as follow-up to "**Financial Disclosure**<br><br>Enter a financial disclosure statement that describes the sources of funding for the	Project DGAPA-PAPIIT IN219424 funded the project.

work included in this submission. Review the submission guidelines for detailed requirements. View published research articles from *PLOS ONE* for specific examples.<br><br>This statement is required for submission and **will appear in the published article** if the submission is accepted. Please make sure it is accurate.<br><br>**Funded studies**<br>Enter a statement with the following details:<br>• Initials of the authors who received each award<br>• Grant numbers awarded to each author<br>• The full name of each funder<br>• URL of each funder website<br>• Did the sponsors or funders play any role in the study design, data collection and analysis, decision to publish, or preparation of the manuscript?<br><br>Did you receive funding for this work?"	
Please select the country of your main research funder (please select carefully as in some cases this is used in fee calculation).<br>as follow-up to "**Financial Disclosure**<br><br>Enter a financial disclosure statement that describes the sources of funding for the work included in this submission. Review the submission guidelines for detailed requirements. View published research articles from *PLOS ONE* for specific examples.<br><br>This statement is required for submission and **will appear in the published article** if the submission is accepted. Please make sure it is accurate.	MEXICO - MX

**Funded studies**<br>Enter a statement with the following details:<br>• Initials of the authors who received each award<br>• Grant numbers awarded to each author<br>• The full name of each funder<br>• URL of each funder website<br>• Did the sponsors or funders play any role in the study design, data collection and analysis, decision to publish, or preparation of the manuscript?<br><br>Did you receive funding for this work?"	
**Competing Interests**<br><br>Use the instructions below to enter a competing interest statement for this submission. On behalf of all authors, disclose any competing interests that could be perceived to bias this work—acknowledging all financial support and any other relevant financial or non-financial competing interests.<br><br>This statement is required for submission and **will appear in the published article** if the submission is accepted. Please make sure it is accurate and that any funding sources listed in your Funding Information later in the submission form are also declared in your Financial Disclosure statement.<br><br>View published research articles from *PLOS ONE* for specific examples.	The authors have declared that no competing interests exist.

**NO authors have competing interests**<br><br>Enter: *The authors have declared that no competing interests exist.*<br><br>**Authors with competing interests**<br><br>Enter competing interest details beginning with this statement:<br><br>*I have read the journal's policy and the authors of this manuscript have the following competing interests: [insert competing interests here]*<br><br>* typeset	
**Ethics Statement**<br><br>Enter an ethics statement for this submission. This statement is required if the study involved:<br><br>• Human participants<br>• Human specimens or tissue<br>• Vertebrate animals or cephalopods<br>• Vertebrate embryos or tissues<br>• Field research<br><br>Write "N/A" if the submission does not require an ethics statement.<br><br>General guidance is provided below. Consult the submission guidelines for detailed instructions. **Make sure that all information entered here is included in the Methods section of the manuscript.**	N/A

**Format for specific study types**<br><br>**Human Subject Research (involving human participants and/or tissue)**<br>• Give the name of the institutional review board or ethics committee that approved the study<br>• Include the approval number and/or a statement indicating approval of this research<br>• Indicate the form of consent obtained (written/oral) or the reason that consent was not obtained (e.g. the data were analyzed anonymously)<br><br>**Animal Research (involving vertebrate animals, embryos or tissues)**<br>• Provide the name of the Institutional Animal Care and Use Committee (IACUC) or other relevant ethics board that reviewed the study protocol, and indicate whether they approved this research or granted a formal waiver of ethical approval<br>• Include an approval number if one was obtained<br>• If the study involved *non-human primates*, add *additional details* about animal welfare and steps taken to ameliorate suffering<br>• If anesthesia, euthanasia, or any kind of animal sacrifice is part of the study, include briefly which substances and/or methods were applied<br><br>**Field Research**<br><br>Include the following details if this study involves the collection of plant, animal, or other materials from a natural setting:<br>• Field permit number<br>• Name of the institution or relevant body that granted permission	
**Data Availability**<br><br>Authors are required to make all data underlying the findings described fully available, without restriction, and from the time of publication. PLOS allows rare exceptions to address legal and ethical concerns. See the PLOS Data Policy and FAQ for detailed information.	Yes - all data are fully available without restriction

A Data Availability Statement describing where the data can be found is required at submission. Your answers to this question constitute the Data Availability Statement and **will be published in the article**, if accepted.<br><br>**Important:** Stating 'data available on request from the author' is not sufficient. If your data are only available upon request, select 'No' for the first question and explain your exceptional situation in the text box.<br><br>Do the authors confirm that all data underlying the findings described in their manuscript are fully available without restriction?	
**Describe where the data may be found in full sentences. If you are copying our sample text, replace any instances of XXX with the appropriate details.**<br><br>• If the data are **held or will be held in a public repository**, include URLs, accession numbers or DOIs. If this information will only be available after acceptance, indicate this by ticking the box below. For example: *All XXX files are available from the XXX database (accession number(s) XXX, XXX.*).<br>• If the data are all contained **within the manuscript and/or Supporting Information files**, enter the following: *All relevant data are within the manuscript and its Supporting Information files.*<br>• If neither of these applies but you are able to provide **details of access elsewhere**, with or without limitations, please do so. For example:<br><br>*Data cannot be shared publicly because of [XXX]. Data are available from the XXX Institutional Data Access / Ethics Committee (contact via XXX) for researchers who meet the criteria for access to confidential data.*<br><br>*The data underlying the results presented in the study are available from (include the name of the third party*	All relevant data are within the manuscript and its Supporting Information files

and contact information or URL).<br>• This text is appropriate if the data are owned by a third party and authors do not have permission to share the data.<br><br>* typeset	
Additional data availability information:	

Manuscript Click here to access/download;Manuscript;Manuscript.docx

Richness and biogeography of Pteridoflora in montane forests of eastern Mexico

J. Daniel Tejero-Díez[1], Perla V. Rodríguez-Sánchez[1], Raúl Contreras-Medina[2], Julio Cesar Ramírez-Martínez[3], Jonathan D. Amith[4], Leccinum J. García-Morales[5], Celia Sanginés-Franco[3], and Isolda Luna-Vega[3]*

[1] Laboratorio de Botánica Estructural, Carrera de Biología, Facultad de Estudios Superiores Iztacala, Universidad Nacional Autónoma de México (UNAM), Tlalnepantla, Estado de México, México

[2] Laboratorio de Biodiversidad, Facultad de Ciencias, Universidad Autónoma Benito Juárez de Oaxaca (UABJO), Oaxaca de Juárez, Oaxaca, México

[3] Laboratorio de Biogeografía y Sistemática, Departamento de Biología Evolutiva, Facultad de Ciencias, Universidad Nacional Autónoma de México (UNAM), Coyoacán, México City, México

[4] Department of Botany, National Museum of Natural History, Smithsonian Institution, Washington DC, United States of America

[5] Departamento de Posgrado e Investigación, Instituto Tecnológico de Ciudad Victoria (ITCV), Victoria, Tamaulipas, México

*Corresponding author

E-mail: luna.isolda@gmail.com (ILV)

Abstract

This study compiles and updates the checklist of ferns and lycophytes from the Sierra Madre Oriental (SMOR), Mexico. We analyzed the distribution and richness of these groups of seedless vascular plants to ensure the accuracy and reliability of the data. We reviewed information on these taxa from regional floristic studies, field explorations, and herbarium specimens. Our updated list includes 567 species, of which 511 are ferns and 56 lycophytes, organized into 37 families and 137 genera. The five ~~richest~~ families are Pteridaceae (131), Polypodiaceae (76), Dryopteridaceae (68), Aspleniaceae (45), and Selaginellaceae (41). The five most ~~diverse~~ genera are *Asplenium* (42), *Elaphoglossum* (30), *Pleopeltis* (24), *Myriopteris* (22), and *Selaginella* (21). The Mexican states with the greatest species richness within the SMOR are Puebla (423 ~~species~~), Hidalgo (322), Querétaro (227), and Tamaulipas (181). The five grid cells with the highest ~~numbers~~, each containing more than 190 species, are situated in the southern part of the SMOR (Puebla and Veracruz). The physiographic provinces with the greatest diversity are Carso Huasteco and the Gran Sierra Plegada, with 523 and 175 species, respectively. Latitudinal analysis indicated low richness at the northern extreme (28 °- 29 ° N, 11 species) and high richness at the southern extreme (19 °- 20 ° N, 469 species), with a notable peak at 24 °- 25 ° N (350 species). The highest diversity within elevation intervals occurs at 1,000-1,500 m (455 species). The SMOR contains 22 species of the pteridoflora classified under various risk categories according to the Mexican Official Norm NOM-059-SEMARNAT-2010, including seven listed on the IUCN Red List and ten protected under CITES. The taxonomic diversity index shows that the SMOR is among Mexico's most species-rich mountain ranges. The species richness documented here in the SMOR accounts for 50-54%

of the ~~pteridoflora~~ diversity recorded in Mexico, increasing to 59.3% based on recent floristic studies conducted in the region.

Keywords: conservation, diversity, ferns, floristics, lycophytes, montane environments.

Introduction

In the montane areas of tropical regions, the lycophytes and ferns are two lineages of vascular plants that are highly diverse worldwide [1, 2, 3]. These two seedless plant groups are known as pteridobionts because they share the same double-phase life cycle [4, 5, 6]. This taxonomic success and abundance in environments with high atmospheric humidity is due to both the sexual phase (zygote formation) and the sporophyte phase (low stomatal reactivity) being sensitive to water deficit [7, 8, 9]. ~~Make~~ them ideal indicators of the conservation quality of tropical montane cloud forests [10, 11]. Therefore, biological studies in biogeography, ecology, or taxonomy are crucial to our understanding of various aspects of ecosystem management.

In the Neotropics, Mexico is one of the countries with a highly diverse pteridoflora [1] according to Hassler [12] with 1,057 species, including 99 endemics. If hybrids and infraspecies are included, the number of taxa increases to 1,123 (sensu Tejero-Díez database). These taxa account for almost 5% of the country's total vascular flora, which includes 23,314 species (sensu [13]), of which 20% are endemic [14]. The Mexican pteridoflora has been extensively studied from a taxonomic perspective [15], and recent nomenclatural updates have been incorporated into the Mexican checklists of ferns and lycophytes [12]. Nonetheless, investigating certain regions —where information remains scattered or incomplete— ~~remains~~ necessary. Ultimately, understanding the historical

processes behind distribution and the ecological and environmental relationships within species is crucial.

Knowledge about the spatial patterns of richness and endemism in Mexican pteridoflora is limited, especially in highly biodiverse areas. The Sierra Madre Oriental (SMOR) is the second largest mountain range and is regarded as a highly biodiverse Mexican biogeographic province [16, 17, 18], where ferns and lycophytes contribute significantly to this biodiversity. The SMOR boasts a higher floristic richness than any other mountainous region in Mexico, with 8,472 species [18]. This richness is attributed to several factors, such as its geographic location on both sides of the Cancer tropic, complex topography, diverse climate types, the presence of nearly all Mexican vegetation types, and a broad elevational gradient ranging from 100 to over 3,600 meters [17].

Previous floristic checklists and biogeographic research related to ferns and lycophytes have been conducted in the main mountain ranges of Mexico, such as the Sierra Madre del Sur [6], the Sierra Madre Oriental [19, 20], the Trans-Mexican Volcanic Belt [21], and the Sierra Madre Occidental [14], as well as the Chiapas Highlands [22]. Recently, floristic checklists of the SMOR covering all vascular plants have been published [18, 19], but our review of the pteridoflora has identified issues like synonymies, incorrect geographic coordinates, mistaken taxonomic identifications or confusion of particular species, as well as species not recorded in the cited studies but present in the SMOR. Additionally, the study by [20], conducted in the SMOR, includes only a subset of leptosporangiate ferns, the most diverse group of ferns, with an estimated 10,323 species worldwide [23].

The aims of this study were: (1) to carry out an updated and revised floristic inventory of the pteridoflora of the SMOR and (2) to analyze the distributional patterns of ferns and lycophytes using three different area units (states, physiographic provinces and grid cells of 30 × 30 minutes latitude/longitude) and to identify elevational and latitudinal richness patterns, (3) to compare the species richness of the SMOR with other Mexican mountain chains and among the physiographic provinces of the SMOR using a taxonomic diversity index, and (4) to revise the categories of threatened taxa of ferns and lycophytes based on the IUCN Red Lists, the Convention on International Trade in Endangered Species of Wild Fauna and Flora (CITES), and the federal regulatory framework established by the Mexican government (NOM-059-SEMARNAT-2010).

Materials and Methods

Study area

The Sierra Madre Oriental (SMOR) is in northeastern Mexico (Fig. 1A) and is the country's second most important and extensive mountainous province system [16, 24]. This montane region is regarded as part of the Mesoamerican hotspot proposed for ferns and lycophytes by [3]. Geologically, the SMOR resulted from the deformation of Cenozoic and Mesozoic marine sedimentary rocks that were uplifted, shortened, and transported north-eastward, creating a fold-and-thrust belt during the Laramide orogeny [25].

The SMOR extends 800 km in length, with an amplitude of 80-100 km [25]. Although various biotic and abiotic criteria have led to different proposed boundaries for the SMOR [16], we adopted the regionalisation framework of [26], which is based on

physiographic regionalisation (Fig. 1A). This regionalisation scheme of the SMOR has also been applied in studies examining the distribution of Cactaceae [27], leptosporangiate ferns [20], and vascular plants [18]. Within this mountain system, 10 physiographic subprovinces are recognized: Sierras Transversales, Pliegues de Saltillo-Parras, Sierras y Llanuras Occidentales, Gran Sierra Plegada, Sierra de San Carlos, Sierra de Tamaulipas, Carso Huasteco, Serranía de El Burro, Sierra de la Paila, and Sierras y Llanuras Coahuilenses. The SMOR constitutes a biogeographic province of Mexico [16, 24], and together with the Trans-Mexican Volcanic Belt, the Sierra Madre del Sur, and the Sierra Madre Occidental, forms part of the Mexican Transition Zone [24]. The SMOR covers parts of the Mexican states of Coahuila, Durango, Guanajuato, Hidalgo, Nuevo León, Puebla, Querétaro, San Luis Potosí, Tamaulipas, Veracruz, and Zacatecas (Figs. 1B, C). Its latitudinal extent spans from the 20th to the 30th parallel, bounded to the north by the Grande (Bravo) River and to the south by the Trans-Mexican Volcanic Belt [16]. Its location places it in contact with key atmospheric systems, including the subtropical high-pressure belt, trade winds, winter cold fronts, and summer tropical storms linked to hurricanes. This, combined with a latitudinal range of ten degrees, and elevational variations from around 100 m in the lowlands of Tamaulipas and San Luis Potosí [17] to 3,713 m above sea level at Cerro El Potosí in Nuevo León (with an average of 1,000 to 1,500 m, Fig. 1C), and the mountain chain running parallel to the Gulf of Mexico opposite the entry of humid winds, produces a significant orographic shadow effect. This results in a complex climate mosaic, with sites experiencing high rainfall, others experiencing dry seasons, and warm- and temperate-climate conditions, and gradients between these conditions [28]. Furthermore, there are isothermal regimes and extremes, with temperature fluctuations of up to 14°C between the hottest and coldest months. Generally, the climate becomes drier and more extreme towards

the north of the SMOR, with its western slope facing the Mexican Plateau. Nearly all recognized Mexican climatic types are present within this area. Vegetation in the SMOR includes xeric shrubland, conifer forests, oak forests, montane cloud forests, tropical forests, and mixed forests [16, 17] (Fig. 1B).

Figure 1. Maps of northeastern Mexico covering the Sierra Madre Oriental (SMOR). A. Physiographic regionalization. B. Land use and vegetation types. C. Elevation. State abbreviations: Coahuila = COAH, Durango = DGO, Nuevo León = NL, Zacatecas = ZAC, Tamaulipas = TAMPS, Querétaro = QRO, Veracruz = VER, Guanajuato = GTO, Puebla = PUE, San Luis Potosí = SLP, Hidalgo = HGO. Map created by the authors using ArcGIS Pro. All vector layers (Physiography, Land Use and Vegetation Series VII, Hypsometry/Topography, and Political Division) were sourced from INEGI (https://www.inegi.org.mx/), used under the Terms of Free Use of Information which permit commercial utilization.

Data sources

We obtained distributional data for ferns and lycophytes from three main sources: 1) the review of floristic studies conducted in the SMOR [4, 18, 19, 29, 30, 31, 32, 33, 34, 35, 36, 37, 38, 39, 40, 41, 42, 43, 44, 45, 46, 47, 48, 49, 50, 51, 52, 53, 54, 55, 56, 57, 58, 59, 60, 61], 2) the revision of herbarium vouchers deposited in herbaria such as ARIZ, ASU, BH, BM, BRIT, BRY, CIB, CNS, DUKE, ENCB, F, FCME, GBH, HGOM, HPC, HUAP, HZAC, IEB, IND, ITCV, LAM, LL, MEXU, MICH, MIN, MMNS, MO, MSC, MU, NCU, NY, P, PH, QMEX, SJNM, TAES, TEX, UAMIZ, US, UAT, UC, UCR-BPS, USF, UT,

UTEP, VDB, WIS, WLM, WTU, XAL, and YU (acronyms following [62], 3) field surveys in some Mexican states, such as Hidalgo, Querétaro, Puebla, and Tamaulipas, to collect botanical specimens. Voucher specimens collected during the fieldwork were deposited at the National Herbarium of Mexico (MEXU), herbarium of the Benemérita Universidad Autónoma de Puebla (HUAP), the herbarium of the Instituto Tecnológico de Ciudad Victoria (ITCV), and the United States National Herbarium (US). We revised all taxonomic names and excluded synonyms and misidentifications, as described in [12]. We reviewed the known distribution as described in [15]. We verified the accurate identification of endemic species and those considered rare (with 1 to 3 specimens in the total number of herbaria reviewed or field collections). The accurate identification of names that did not match expert reports in the literature was also reviewed. We compiled a database from all these information sources, including 4,479 georeferenced records of lycophytes and ferns in the SMOR. Using this information, we created geographic distribution maps of the species with ArcGIS Pro [63].

Since the publication of ‘The Pteridophytes of Mexico’ by [15], new combinations, genera, species, and records of ferns and lycophytes have been published and reported. For these reasons, and in accordance with the International Code of Nomenclature for algae, fungi, and plants (Shenzhen Code) [64], we verified the correct names of each species. The nomenclature and author citations were standardized following the [12] database (except in *Selaginella* sensu [65] and updated to align with the latest integrative and circumscription taxa systematics based on the proposal of the Pteridophyte Phylogeny Group [23].In our database, as is common with herbarium specimens, duplicate samples are found across different botanical collections. In our study, we removed duplicate records from the

biogeographic analysis to ensure unique locations. Additionally, we identified and corrected records with swapped latitude and longitude values. We also excluded specimens with vague localities and inconsistencies. For herbarium specimens lacking geographic coordinates, we used topographic maps at a 1:50,000 scale from the Instituto Nacional de Estadística y Geografía (INEGI) and the previously published locality list for the Sierra Madre Oriental [66] to obtain the corresponding coordinates. Ultimately, we aimed to include only georeferenced and verified data in our study.

To visualize the spatial distribution of collection records and identify potential sampling biases across the SMOR, we created a heatmap from all georeferenced occurrences included in the database. The heatmap was generated in ArcGIS Pro [63] using the Point Density tool, which measures the spatial intensity of records. This method allowed us to highlight areas with high densities of fern and lycophyte collections, enabling the detection of well-sampled regions and zones with limited survey effort. The resulting layer served as a valuable tool for evaluating the dataset's spatial completeness and providing context for subsequent analyses of species richness and distribution patterns.

Biogeographic and diversity analyses

Mexican states were included in the SMOR, physiographic provinces were used to perform the biodiversity analyses, and 30 × 30 minute grid cells were used as units of analysis in ArcGIS Pro [63]. In the grid analysis, we recorded 86 grid cells containing at least one fern or lycophyte species from this framework. The spatial size of grid cells provides a reliable spatial resolution for the distribution compared with other studies using

plants in the SMOR [18, 20, 27] regarding biodiversity data and biological conservation. We selected this scale size because similar scales have been tested in previous studies on biogeography and the diversity of different biological groups of the Mexican biota [67, 68], including ferns and lycophytes [69].

The total expected richness of ferns and lycophytes in the SMOR was estimated using a nonparametric abundance model (Chao1) in EstimateS [70] to evaluate its performance and determine whether it fits the data.

In the grid cell analysis, we used three standard biodiversity metrics (species richness, weighted endemism, and corrected weighted endemism) to measure the biodiversity patterns previously used for different groups of the Mexican pteridoflora [20, 69, 71, 72]. These metrics are more than just theoretical concepts; they are practical tools that have been effectively used in the study of the Mexican pteridoflora [73]. We measured species richness as the total number of species present within each grid cell. Each fern or lycophyte species was counted as present in a grid cell, regardless of whether it was recorded once or multiple times in that cell [27, 74]. The weighted endemism (WE) involves several steps [73]. First, we divided each grid cell occurrence by the number of grid cells in which one species occurs. For example, a fern species limited to a single grid cell was scored as '1' for that grid cell and '0' for all remaining grid cells, whereas a fern species found in five grid cells was scored as '0.2' for each of the five grid cells and '0' for all other grid cells; then we obtained the sum of all score species values for each grid cell. We calculated the corrected weighted endemism (CWE) by dividing the WE value by the number of species in a grid cell [73, 74]. The WE index was considered a biodiversity measure sensitive to species richness, suggesting that richness patterns partially mirror the

WE value [27, 74]. In contrast, the CWE index emphasizes grid cells that contain a proportion of species with limited ranges, which are not necessarily linked to species-rich grid cells [27, 74].

The biodiversity metrics mentioned above were calculated using the software Biodiverse, version 3.0 [75], and mapped in ArcGIS Pro [63]. Grid cells containing only one species were excluded from the CWE analysis because they lack overlapping distributions, which is a key criterion for identifying areas of endemism [76, 77]. Grid cells with the highest scores in the WE index were considered centers of richness, while those with the highest values in the CWE index were regarded as centers of endemism [73, 74].

The geographic distribution of ferns and lycophytes inhabiting the SMOR was established based on the study of [15] as a general framework. We defined nine categories related to the geographic distribution of each species as follows: 1) Cosmopolitan, taxa distributed worldwide; 2) American, taxa distributed across the American continent; 3) Nearctic, taxa found in Mexico (and sometimes in Mesoamerica) and North America; 4) Neotropical, taxa found in Mexico, Central America, the Antilles, and South America; 5) Mesoamerican, taxa distributed in Mexico and Central America; 6) Endemic to the Mexican territory; 7) Endemic to the SMOR, taxa with a known distribution limited to the boundaries of the SMOR; 8) Regional endemic, in adjacent areas to the SMOR; and 9) Introduced taxa.

We determined the frequency of each fern and lycophyte species following the method recommended by [78], based on the number of records for each species and assigned them to their respective frequency classes. This reflects that some species will thrive at the expense of others, leading to differences in species frequency, with a few

species having many individuals and others being rare. We divided the species into five classes of record frequency according to the following ranges: A, 1-11 records; B, 12-23; C, 24-35; D, 36-47; and E, 48-59.

Typically, the species richness of a specific flora is expressed as the number of species or as a biodiversity index that accounts for the number of species per area on a logarithmic scale. To compare the richness of lycophytes and ferns among the main Mexican mountain ranges and the physiographic provinces of the SMOR, we used the taxonomic diversity index (IB), which accounts for the number of species per area on a logarithmic scale, following the formula IB = S/Ln A, where S is the number of recorded species and A is the size of the area [79]. Therefore, using biodiversity indices is crucial for comparing sampled areas of different sizes. Using this formula, we assessed species richness across geographic regions, specifically the main Mexican mountain ranges and the physiographic provinces of the SMOR.

Finally, we assessed the species richness of lycophytes and ferns across different latitudes and elevation gradients. The SMOR covers a wide elevation range from 100 m to 3,713 m above sea level. Based on this, we divided the elevation into eight intervals of 500 m each to illustrate species distribution along this gradient in a graph. These intervals have been used in previous studies on ferns and lycophytes in other Neotropical areas [14, 80, 81, 82], so we applied them to our data for comparison.

We divided the SMOR into 1° latitudinal belts to assess the relationship between species richness and latitude. Additionally, species richness was calculated for all grid cells within each 1° latitude belt from 19°-20° N to 28°-29° N. The species richness values for

each latitudinal belt were determined using the taxonomic diversity index (IB) for that belt [83, 84]. We presented the results of this analysis on a graph showing the taxonomic diversity index values against latitude.

Conservation status

We revised the threatened taxa categories for ferns and lycophytes inhabiting the SMOR in accordance with the Mexican federal regulatory framework [85], the IUCN Red Lists [86], and the Convention on International Trade in Endangered Species of Wild Fauna and Flora (CITES) [87]. Regarding the Mexican risk categories, we reviewed the Mexican Official Norm NOM-059-SEMARNAT-2010 [85] (hereafter, NOM-059) to determine how many ferns or lycophytes are listed and to confirm the assigned risk category for each species. The NOM-059 is the official document issued by the Mexican government, and lists threatened species of plants, fungi, and animals [85]. This document outlines the federal regulatory framework, including the specifications for inclusion and analysis to assess the risk of threatened species. Similarly, the risk categories were assessed according to the IUCN Red List of Threatened Species [86] and the CITES [87] lists. Comparing the IUCN Red List with the Mexican Official Norm NOM-059, some categories can be considered equivalent, such as the threatened category in the Mexican Official Norm to the vulnerable category of the IUCN. In contrast, the special protection category of the NOM-059 includes some minor categories from the IUCN [88].

Results

Based on examined herbarium specimens, web databases, specialized literature, and specimens collected during field expeditions, we compiled a database of 4,479 georeferenced accession records (Fig. 2A). These records correspond to 567 species from the SMOR, belonging to 137 genera and 37 families. From this checklist, 511 species (34 families, 123 genera) are from Polypodiidae, while 56 (3 families, 13 genera) belong to Lycopodiidae. We identified 211 species of ferns and lycophytes that were not previously documented in the floristic studies conducted by [19] and [68] in the SMOR. Of the total names referenced in the studies, 56 were rejected or excluded because they were synonyms or did not represent species found in the study area or the country.

The heatmap (Fig. 2B) visually shows the record density and reveals spatial patterns in sampling effort. It displays the distribution of collection records, with yellow and red colours indicating areas with a high number of records, and blue indicating areas with fewer records, interpreted as well-sampled and under-sampled regions, respectively. The highest concentration of records is in the central part of the country.

Figure 2. A. Map of the SMOR showing georeferenced records of ferns and lycophytes. B. Heatmap showing the sampling effort of ferns and lycophytes. Map created by the authors using ArcGIS Pro. Physiographic regionalization (SMOR) and Political Division layers were sourced from INEGI (https://www.inegi.org.mx/), used under the Terms of Free Use of Information which permit commercial utilization.

We found that the most diverse families (20 or more species) in the SMOR are Pteridaceae (131 species; 23.1 %), Polypodiaceae (76; 13.4 %), Dryopteridaceae (68; 12.0 %), Aspleniaceae (45; 7.9 %), Selaginellaceae (41; 7.2 %), Thelypteridaceae (34; 6.0 %), and Hymenophyllaceae (28; 4.9 %) (Table 1). Nine families are represented by a single species each: Culcitaceae, Didymochlaenaceae, Lindsaeaceae, Lonchitidaceae, Lygodiaceae, Onocleaceae, Plagiogyraceae, Saccolomataceae, and Schizaeaceae.

Table 1. Families of ferns and lycophytes represented in the SMOR. We recorded the number of species and genera in each family and endemics.

**Families**	**Species (%)**	**Genera**	**Endemic species**	**Most diverse genera**
Anemiaceae	12 (2.1)	1	*Anemia* 1	
Aspleniaceae	45 (7.9)	2	*Asplenium* 6	*Asplenium* (42)
Athyriaceae	19 (3.4)	3		
Blechnaceae	12 (2.1)	5	*Woodwardia* 1	
Culcitaceae	1 (0.2)	1		
Cyatheaceae	10 (1.8)	3		
Cystopteridaceae	2 (0.4)	1		
Dennstaedtiaceae	13 (2.3)	6		
Dicksoniaceae	2 (0.4)	2		
Didymochlaenaceae	1 (0.2)	1		
Dryopteridaceae	68 (12.0)	12	*Ctenitis* 2, *Dryopteris* 1, *Elaphoglossum* 10, *Megalastrum* 2, *Phanerophlebia* 1	*Elaphoglossum* (30)
Equisetaceae	4 (0.7)	1		
Gleicheniaceae	7 (1.2)	4		
Hymenophyllaceae	28 (4.9)	**7**	*Didymoglossum* 1	

Families	Species (%)	Genera	Endemic species	Most diverse genera
Isoetaceae	2 (0.4)	1	*Isoetes* 2	
Lindsaeaceae	1 (0.2)	1		
Lomariopsidaceae	2 (0.4)	1	*Lomariopsis* 1	
Lonchitidaceae	1 (0.2)	1		
Lycopodiaceae	13 (2.3)	6	*Huperzia* 1	
Lygodiaceae	1 (0.2)	1		
Marattiaceae	4 (0.7)	2		
Marsileaceae	3 (0.5)	1		
Nephrolepidaceae	6 (1.1)	1		
Onocleaceae	1 (0.2)	1		
Ophioglossaceae	11 (1.9)	5		
Osmundaceae	2 (0.4)	2		
Plagiogyriaceae	1 (0.2)	1		
Polypodiaceae	76 (13.4)	18	*Campyloneurum* 1, *Pecluma* 1, *Pleopeltis* 10, *Polypodium* 3	*Pleopeltis* (24)
Psilotaceae	3 (0.5)	1		
Pteridaceae	131 (23.1)	26	*Adiantum* 1, *Argyrochosma* 2, *Aspidotis* 1, *Astrolepis* 1, *Bommeria* 2, *Gaga* 5, *Myriopteris* 4, *Notholaena* 6, Pellaea 2, Ynesmexia 1	*Myriopteris* (22)
Saccolomataceae	1 (0.2)	1		
Salviniaceae	2 (0.4)	1		
Schizaeaceae	1 (0.2)	1		
Selaginellaceae	41 (7.2)	6	*Bryodesma* 4, *Lepidoselaginella* 2, *Pulviniella* 1, *Selaginella* 7	*Selaginella* (21)
Tectariaceae	3 (0.5)	1		
Thelypteridaceae	34 (6.0)	8	*Amauropelta* 1, *Goniopteris* 1, *Pelazoneuron* 2	

Families	Species (%)	Genera	Endemic species	Most diverse genera
Woodsiaceae	3 (0.5)	1	*Physematium* 1	
**TOTAL 37**	**567** (100.0)	**137**	**89**	**139**

Table 2. Species with more records and recorded in more ~~SMOR~~ grid cells.

Species	Number of records	Grid cells
Pleopeltis polylepis (Roemer ex Kunze) T. Moore	57	25
Pleopeltis plebeia (Schltdl. & Cham.) A. R. Sm. & Tejero	56	20
Polypodium plesiosorum Kunze	53	27
Phlebodium pseudoaureum (Cav.) Lellinger	52	17
Tectaria heracleifolia (Willd.) Underw.	47	20
Campyloneurum angustifolium (Sw.) Fée	41	15
Adiantum capillus-veneris L.	40	26
Pelazoneuron ovatum (R.P. St. John) A.R.Sm. & S.E. Fawc.	40	21
Llavea cordifolia Lag.	36	19
Pleopeltis crassinervata (Fée) T. Moore	36	10
Adiantum tenerum Sw.	35	13
Asplenium monanthes L.	35	18
Astrolepis sinuata (Lag. ex Sw.) D.M. Benham & Windham	35	26
Blechnum appendiculatum Willd.	34	15
Adiantum poiretii Wikstr.	33	15
Pleopeltis acicularis (Weath.) A.R. Sm. & T. Krömer	33	11
Pteridium aquilinum (L.) Kuhn	33	13
Anemia mexicana Klotzsch	31	20
Notholaena candida (M. Martens & Galeotti) Hook.	31	20
Pteris cretica L.	31	19
Astrolepis integerrima (Hook.) D.M. Benham & Windham	30	23

**Species**	**Number of records**	**Grid cells**
Pleopeltis polypodioides (L.) E.G. Andrews & Windham	30	20
Anemia adiantifolia (L.) Sw.	29	19
Pleopeltis guttata (Maxon) E. G. Andrews & Windham	29	21
Polypodium arcanum Maxon	29	15
Remaining taxa	3,543	2,289
**Total**	**4,479**	**2,757**

State diversity in the SMOR

Our analysis indicates that Puebla has the highest number of fern and lycophyte species, with 423 species (31.9 species/km^2), followed by Hidalgo and Querétaro, which have 321 (23.3 species/km^2) and 227 (17.6 species/km^2), respectively. Tamaulipas and Veracruz have 181 (9.8 species/km^2) and 117 (8.3 species/km^2), respectively, while the other states of the SMOR have fewer than 100 species each. Endemic ferns or lycophytes confined to a single Mexican state were recorded in Puebla and Tamaulipas, with three taxa, and in Nuevo León and San Luis Potosí, with two species.

From the total identified pteridoflora of the SMOR area as defined by [26], we identified an essential subset of 123 species representing new records for the SMOR. A subset of 40 species has an updated distributional range, previously recorded in the SMOR, and is now documented in other states not previously reported in the literature (Fig. 3).

Figure 3. New pteridophyte records for the ~~SMOR~~ (123 species) and species with an extended distribution in other states from the SMOR (40 ~~spp.~~).

Eleven of the new records belong to *Elaphoglossum*, seven to *Selaginella*, and six are filmy ferns of the genus *Hymenophyllum*. A total of 41 genera are represented by a single species within the new records in the SMOR. The others have between two and five species. The genera with the most species showing range extension into the SMOR are *Notholaena* and *Amauropelta*, with five and four taxa, respectively. Overall, 16 genera have one species extending its range in the SMOR, while the rest are represented by two or three species.

Common vs. rare species

As is common in biogeographic studies based on herbarium data, few fern species are represented by a disproportionate number of specimens, while many are represented by only a few (Fig. 4). In our study, only four species have more than 50 records (*Phlebodium pseudoaureum, Pleopeltis polylepis, Pleopeltis plebeia*, and *Polypodium plesiosorum*), which contrasts with several other species limited to just one or three localities (252 taxa). These latter data account for approximately 50% of the pteridoflora of this mountain range.

Figure 4. Record frequency for each species represented in the SMOR.

Grid cell species richness and endemism indices

We analyzed 86 grid cells from the SMOR (Fig. 5A), each containing at least one record. Species richness values ranged from 1 to 307 species per grid cell (Fig. 5B). The main center of pteridoflora diversity in the southern part of the SMOR (states of Puebla, Hidalgo,

Querétaro, and Veracruz) consisted of nine neighbouring grid cells with the highest species richness scores (>90), ranging from 96 to 307 species (Fig. 5B). The tenth grid cell with the highest richness score (95) is located in Nuevo León (BB). Among the top-scoring grid cells, BX, CC, CD, and CG have the highest number of species (>200). These high-richness grid cells contrast with other parts of the SMOR, where collections are sparse or absent, as in 13 grid cells with only one fern or lycophyte species and eight grid cells with just two taxa recorded (Fig. 5B).

Figure 5. A. Division of the SMOR into grid cells of 30 minutes by side and latitudinal belts of one geographical degree. B. Species richness of the pteridoflora in the SMOR. Map created by the authors using ArcGIS Pro. Physiographic regionalization (SMOR) and Political Division layers were sourced from INEGI (https://www.inegi.org.mx/), used under the Terms of Free Use of Information which permit commercial utilization.

Polypodium plesiosorum (27), *Adiantum capillus-veneris* and *Astrolepis sinuata* (26 each), *Pleopeltis polylepis* (25), and *Astrolepis integerrima* (23) were found in more grid cells, followed by *Pelazoneuron ovatum* and *Pleopeltis guttata* (21) (Table 2). Conversely, 150 species are only represented in one grid cell. Ferns and lycophytes are mainly distributed in temperate forests, such as oak and pine-oak forests, and in tropical montane cloud forests. However, some species prefer other types of Mexican vegetation that receive less rainfall, such as the arid scrub and tropical deciduous forest. Temperate landscapes mainly influence the key distribution patterns observed in this study.

We undertook an effort-weighted sampling method for our manuscript. In our study using the Chao1 richness estimator, we identified 567 species. According to the estimator, the average estimated richness at this sampling level was about 670 species, with a 95% confidence interval of approximately 632-729 species (Fig. 6). The difference between the lower and upper confidence limits is roughly 100 species, indicating moderate uncertainty around the estimate, as expected in communities with many rare or low-detectability taxa.

Figure 6. Species accumulation curve for ferns and lycophytes of the Sierra Madre Oriental (SMOR) using the Chao1 richness estimator. The observed species richness is shown as a solid line. The Chao1 estimator is represented by three curves: the mean estimated richness and its lower and upper confidence limits (95%).

Comparing the observed richness (567 species) to the Chao1 mean estimate (≈670 species), our data falls short by about 100 species from the estimated total richness. This

suggests that our sampling completeness is roughly 85%, which is relatively high, because Chao1 uses the number of very infrequently observed species to infer unseen diversity, tending to produce higher richness estimates when rare species are relatively common in the sample [89, 90]. These curves show that we've captured more than 85% of the expected species at a sampling site, indicating that this type of analysis is feasible [91].

Overall, these results suggest that although the sampling did not fully capture the total estimated richness, it still documented a significant portion of the community. Using Chao1, the accumulation curves for fern and lycophyte species in the SMOR did not reach a defined asymptote, remaining above the observed richness.

Endemism indexes

Figures 7A and 7B display the values of weighted endemism (WE) and corrected weighted endemism (CWE) for ferns and lycophytes of the SMOR. For WE, values ranged from 0.04 to 115.8 across the grid cells analyzed (Fig. 7A). The WE values showed a pattern similar to that of species richness. We found that nearly all grid cells with the highest species richness scores also recorded the highest WE values (>16), except for the grid cell BW.

The corrected weighted endemism values ranged from 0.05 to 0.48 (Fig. 7B), excluding grid cells with only one species, as previously established. The grid cells with the highest CWE values (>0.25) are spread throughout the SMOR, and only one grid cell (CD) with the highest species richness is included.

Figure 7. A. Weighted endemism (WE). B. Corrected weighted endemism (CWE). Map created by the authors using ArcGIS Pro. Political Division layers were sourced from INEGI (https://www.inegi.org.mx/), used under the Terms of Free Use of Information which permit commercial utilization.

Elevation patterns

The pteridoflora in the SMOR is found across the entire elevation range of this mountain range (100 to 3700 m). As is typical in Neotropical mountains, species richness in the SMOR exhibits a hump-shaped elevational pattern, with the highest diversity at mid-elevations (Fig. 8). The greatest number of species occurs between 1000 and 1500 m (455 species), decreasing at both higher and lower elevations.

Figure 8. Elevational distribution of the pteridoflora in the SMOR.

Latitudinal patterns

We believe that the pteridoflora of the SMOR did not strictly follow a latitudinal pattern. Species diversity does not consistently decrease or increase with latitude (Fig. 9). Our analysis of latitude within 1 ° geographic belts (Fig. 5A) revealed low richness at the northern extreme (28°-29° N, 11 species) and high richness at the southern extreme (19°-20° N, 469 species). Another peak in richness occurs at the 24 °- 25 ° N belt (with 350 species). The decline in species richness is more apparent from the 24°-25° N belt to the northernmost point (28°-29° N).

Figure 9. Latitudinal belts of one geographic degree and their values of the taxonomic biodiversity index. The numbers over each column represent the species recorded in each latitudinal belt.

Geographic distribution patterns

Regarding the geographic distribution of ferns and lycophytes inhabiting the SMOR, the Neotropical and Mesoamerican components are the best represented, with 238 and 119 species, respectively (Fig. 10) (see Appendix). They are followed by the endemic component to Mexico, which has 64 species ~~(Fig. 10)~~. Other significant components include the Nearctic and American, represented by 44 and 41 species. The high proportion of Neotropical and Mesoamerican components in the SMOR enhances the significance of this mountain range within the Mexican Transition Zone. Each remaining geographic component has fewer than 30 species (Fig. 10).

We recorded 21 species that are endemic to the SMOR: *Asplenium dianae*, *Asplenium semipinnatum*, *Asplenium ultimum*, *Bryodesma carnerosanum*, *Gaga apiacea*, *Gaga hintoniorum*, *Goniopteris schaffneri*, *Isoetes tamaulipana*, *Myriopteris chipinquensis*, *Myriopteris maxoniana*, *Notholaena brachycaulis*, *Notholaena brevistipes*, *Notholaena jacalensis*, *Notholaena leonina*, *Pellaea notabilis*, *Pellaea ribae*, *Pleopeltis ×gracilis*, *Pleopeltis ×pueblensis*, *Pleopeltis fallacissima*, *Pulviniella gypsophila*, and *Woodwardia martinezii*. At least five other species are closely associated with the SMOR and have geographic distributions almost restricted to this mountain chain (Appendix).

Figure 10. Geographic distribution patterns of the pteridoflora of the SMOR. The abbreviations are: (Cos) Cosmopolitan; (A) American; (Na) Nearctic; (Nt) Neotropical; (M) Mesoamerican; (Mx) Endemic to Mexico; (End) Endemic to the SMOR; (Reg) Regional endemic; (I) Introduced.

Risk categories

There are 20 fern species and two lycophytes listed in the Mexican Official Norm NOM-059 [85]. These species are shown in Table 3. Among them, 12 are classified as special protection category (Pr), 7 as threatened (A), and 3 as at risk of extinction (P). The three species are *Cyathea costaricensis*, *Cyathea godmanii*, and *Selaginella porphyrospora*. According to the Red Lists of Threatened Species by the IUCN [86], seven fern taxa are categorized as least concern (LC), indicating a low risk of extinction (see Table 3). Only members of Cyatheales are included in Appendix II of CITES [87], comprising eight Cyatheaceae and two Dicksoniaceae (see Table 3).

Table 3. Threatened species of ferns and lycophytes of the SMOR and their risk categories.

Family	Species	NOM-059	IUCN	CITES
Aspleniaceae	*Asplenium auritum* Sw.	A		
	Asplenium formosum Willd.		LC	
	Asplenium serratum L.	A		

Family	Species	NOM-059	IUCN	CITES
Culcitaceae	*Culcita coniifolia* (Hook.) Maxon	Pr		
Cyatheaceae	*Alsophila firma* (Baker) D. S. Conant	Pr		Appendix II
	Cyathea bicrenata Liebm.	Pr		Appendix II
	Cyathea costaricensis (Mett. ex Kuhn) Domin	P		Appendix II
	Cyathea fulva (M. Martens & Galeotti) Fée	Pr		Appendix II
	Cyathea godmanii (Hook.) Domin	P		
	Cyathea microdonta (Desv.) Domin	Pr		Appendix II
	Cyathea myosuroides (Liebm.) Domin	Pr		Appendix II
	Cyathea schiedeana (C. Presl) Domin	Pr		Appendix II
	Cyathea tuerckheimii R. M. Tryon	Pr		
	Sphaeropteris horrida (Liebm.) R. M. Tryon	Pr		Appendix II
Dicksoniaceae	*Dicksonia navarrensis* Christ	Pr		Appendix II

Family	Species	NOM-059	IUCN	CITES
	Lophosoria quadripinnata (J. F. Gmel.) C. Chr.			Appendix II
Equisetaceae	*Equisetum hyemale* L. var. *affine* (Engelm.) A. A. Eaton		LC	
Lycopodiaceae	*Phlegmariurus dichotomus* (Jacq.) W.H. Wagner	A		
Marattiaceae	*Marattia laxa* Kunze	Pr		
	Marattia weinmanniifolia Liebm.	Pr		
Osmundaceae	*Osmunda spectabilis* Willd.		LC	
Polypodiaceae	*Campyloneurum phyllitidis* (L.) C. Presl	A		
	Serpocaulon triseriale (Sw.) A. R. Sm.	A		
Psilotaceae	*Psilotum ×complanatum* Sw.	A		
	Psilotum flaccidum Wall.	A		
Pteridaceae	*Adiantum capillus-veneris* L.		LC	
	Pteris vittata L.		LC	
Selaginellaceae	*Selaginella porphyrospora* A. Braun	P		
Thelypteridaceae	*Cyclosorus interruptus* (Willd.) H. Itô		LC	

Family	Species	NOM-059	IUCN	CITES
	Goniopteris tetragona (Sw.) C. Presl		LC	

Abbreviations. Risk categories of the NOM-059: (A) Threatened; (Pr) Special protection; (P) Endangered. IUCN: (LC) Least concern category.

Discussion

Our study increased the number of ferns and lycophytes recorded in the SMOR compared to earlier floristic surveys conducted in this Mexican mountain range [18, 19]. We documented 567 species, including 511 ferns and 56 lycophytes, thereby expanding our understanding of these plant groups and the Mexican pteridoflora. Our findings reflect an increase of 211 species over the 356 species in the SMOR, as documented by [12, 19], which reported 1,055 species in Mexico. Tejero's personal database (2025) includes 1,124 taxa, encompassing subspecies, varieties, and hybrids. Given this diversity, the new total of species recorded here in the SMOR represents approximately 50-54% of the Mexican pteridoflora. This study also documents at least 56 names and/or species that were excluded following taxonomic refinement and review.

Our study, mainly based on specimen revisions from herbaria and fieldwork, uncovers new records and range extensions for over 100 species of pteridoflora in the SMOR. Our revision identifies 123 species in 62 genera as new for the area and 40 species in 25 genera showing range extensions within this mountain chain. *Elaphoglossum* and *Hymenophyllum* are the fern genera with the most recent records in the SMOR; a similar

situation occurs in an inventory of the pteridoflora in Veracruz [43], where *Elaphoglossum* and filmy ferns were also reported with the highest number of new records among ferns. Our findings confirm that ongoing fieldwork in the mountains of the Mexican Transition Zone could uncover new records and range extensions of the Mexican pteridoflora [43], even in regions that have already been floristically studied, such as the SMOR (e.g., [18, 19].

The geographic position, its alignment with the oceanic edge, and the size of the SMOR influence the observed patterns of differential richness. A common pattern of species richness along the latitudinal gradient, which shows an increase from the north to the tropics, was disrupted. Despite over 200 years of research, the factors driving geographic patterns of species richness remain largely misunderstood [92]. In the case of the SMOr, an intermediate zone of the gradient (24°–25° N) was found to have a relatively high proportion of pteridoflora compared to the southern latitudinal zones (19°–20° N), with 469 species (especially Puebla; 31.9 species/km²) and the northern zone with the lowest (Coahuila 2.7 species/km²).

Most likely, the ecological factor responsible for this intermediate peak in species richness can be attributed to the combination of the broader SMOR band with the effect of orographic shadow, which creates an unparalleled environmental heterogeneity in the mountain range. Additionally, the states on the oceanic slope are more diverse (Querétaro 17.6) than those on the interior (Guanajuato 1.2 species/km²). By analyzing both the latitude and orographic shadow patterns, we can explain the high richness at southern latitudes (20-21°; HGO, PUE, and VER) and the intermediate richness at latitudes 25-26°,

where richness increases from west to east across the mountain (DGO, COAH, and ZAC; and NL, TAMS) (Fig. 9).

As observed in the mountainous regions of southeastern Mexico [6], the SMOR shows a strong Neotropical influence. Many Neotropical and Mesoamerican elements are well-represented in the Sierra Madre del Sur and the Chiapas Highlands in southeastern Mexico. In the SMOR, the flowering plants display a marked Nearctic influence [18], contrasting with the predominant Neotropical affinities of the pteridoflora reported here. Compared to the Sierra Madre Occidental [14], the SMOR showcases the typical association of Neotropical ferns in montane ecosystems due to its high humidity levels, like those found on the Gulf of Mexico slope, and hosts several of the most diverse epiphytic fern clades, including *Asplenium*, *Elaphoglossum*, Hymenophyllaceae, and Polypodiaceae [3]. The diversity of pteridoflora in the SMOR and the presence of endemic species support the idea of a tropical Mexican fern hotspot, with the SMOR forming part of one of the main regional hotspots in the Neotropics recognized by [93].

Pteridaceae, Dryopteridaceae, and Polypodiaceae are regarded as some of the most diverse fern families worldwide. Some genera and subfamilies reflect the dominant ecological zones in the SMOR. Pteridaceae is a successful group represented there by 131 species and is classified into three major bioclimatic groups: adiantoids, pteroids, and cheilanthoids. The cheilanthoid ferns (*Argyrochosma, Astrolepis, Gaga, Myriopteris, Notholaena*, *Pellaea*) are primarily found on the north and western slopes facing the SMOR, which features xerophytic and seasonal temperate vegetation. Conversely, the adiantoid ferns are mainly present in the eastern part of the SMOR, characterized by a

warm, humid climate. Lastly, the remnant pteroid group (*Pteris*, *Vittaria*) is predominantly located in the temperate, humid montane region [94].

The other two fern families with high species richness in the SMOR are Dryopteridaceae and Polypodiaceae, mainly inhabiting the TMCF on oceanic slopes [19, 95]. In the SMOR, Polypodiaceae includes 18 genera and 76 species, exhibiting the greatest variation in form among ferns, including epiphytic species [96]. Some genera, such as *Campyloneurum, Pecluma* (both with 10 species), *Polypodium* (11 species), and *Pleopeltis* (24 taxa), are mainly represented by epiphytic forms in the SMOR [96]. Dryopteridaceae are primarily found in temperate and tropical montane habitats [95]; they comprise 12 genera and 68 species, mostly terrestrial, except for *Elaphoglossum*, a diverse genus with 30 species, some terrestrial and some epiphytic [97]. *Elaphoglossum, Pleopeltis*, and *Polypodium* occupy the vertical space of trees and contribute to the high diversity of these forests compared to other regions of the warm zone. Among lycophytes, the richest family in the SMOR is Selaginellaceae, represented solely by *Selaginella*, with 41 species [12]. The list of lycophytes presented in this study is based on [65]. Members of Selaginellaceae thrive under a wide range of climates and soil types. Species with homophyllous leaves (*Bryodesma*) and heterophyllous, rosette-forming leaves (*Lepidoselaginella*) are poikilohydric, drought-adapted xerophytes [98, 99]. The heterophyllous species are diverse in tropical forests worldwide, predominantly in secondary environments [100]. Both mesic and tropical-xeric ecological zones are well represented in the SMOR. The same is true for other Mexican montane ranges, such as the Sierra Madre Occidental [14] and the Sierra Madre del Sur [6], which, along with the Trans-Mexican Volcanic Belt and SMOR, form the Mexican Transition Zone sensu [101]. Conversely, in the southern Mexican mountains

with homogeneous mesic environments, such as the Chiapas Highlands, there are only 24 species [22].

In the heatmap, the concentration of collection records is located in the central part of the country, corresponding to the southern part of the SMOR. This map shows that collecting effort is not randomly distributed, as is often the case in biodiversity studies [102], and the disproportion of records in the southern part of the SMOR may be linked to accessible geographic locations such as roadsides and urban areas, as well as proximity to research institutions in central Mexico with active botanical research. This pattern has been previously suggested for biodiversity studies based on herbarium specimens [102, 103, 104]. The higher number of collection records and species of ferns and lycophytes in the southern SMOR can also be explained by the higher rainfall and warmer temperatures in this region [28]. These climatic conditions support the presence and abundance of these vascular plants in the tropical montane cloud forests, which are preferred by many botanists for the study and field collection of Mexican ferns and lycophytes, as in other parts of the country [5, 95].

The grid cells with high species richness scores include families and genera that typically inhabit humid environments [4]. The convergence of certain families, such as Lycopodiaceae, Selaginellaceae, Aspleniaceae, Athyriaceae, Cyatheaceae, Dryopteridaceae, Hymenophyllaceae, Polypodiaceae, Pteridaceae, and Thelypteridaceae in these highly diverse grid cells reflects complex plant assemblages in these areas, due to the presence of different phylogenetic lineages of the Mexican pteridoflora, linked to epiphytic and terrestrial lifestyles [92].

The estimator values used showed similar behavior and remained close to the observed values, indicating that a good sampling of the pteridophyte flora was achieved [90]. Although the number of pteridophyte species reported in this study is much higher than that recorded in previous research (e.g., [19]), the analysis of the species accumulation curves suggests that more species can still be recorded to complete the list of ferns and lycophytes of the SMOR.

The WE values obtained for ferns and lycophytes in the SMOR are very similar to the species richness. Nearly all grid cells with high species richness scores (except BW) also showed high WE index values. This correlation between species richness and WE values has been suggested in previous studies [27, 73, 74]. The CWE index emphasizes species with limited distribution, so only one grid cell (CD) is represented in the WE with the highest values. The grid cells with high CWE values (≥0.25 and containing two or more recorded species) are spread across different zones of the SMOR and are influenced by the geographic distribution of the species present in these grid cells, some of which are endemic to the SMOR, such as *Asplenium dianae, A. semipinnatum, A. ultimum, Notholaena brachycaulis,* and *Pellaea ribae*.

We regard the CWE index as an essential measure of endemism because it is independent of species richness [69, 73, 74], as it includes species endemic to the SMOR and Mexico. We recommend that the grid cell CD, which appears in both WE and CWE indexes and is recognized as having the highest diversity (307 species), should be a priority area for conserving the Mexican pteridoflora. Further research and fieldwork are needed in grid cells with the highest CWE scores to boost the number of records and species in these areas.

Following the “World Ferns” sensu [12], some species are found in only one Mexican state. Our study revealed that many of these species (e.g., *Pecluma liebmannii* and *Notholaena brevistipes*), previously recorded as endemic to just one state, are also found in another Mexican state. These two species, along with *Ctenitis bullata*, ~~*Pecluma liebmannii*,~~ *Pleopeltis* ×*melanoneuron*, and *Selaginella subrugosa*, are documented in our study for the first time in one to three Mexican states, changing their endemism status from local endemic to regional endemic.

Considering the high diversity of the pteridoflora in the SMOR (567 species), we observed that only a small percentage of these vascular plants are classified as threatened by international and national agencies. Regrettably, only 22 species are listed under some risk category in the NOM-059 [85] by the Mexican government, and seven are in the least concern (LC) category of the IUCN [86]. This low number of threatened species suggests that other fern and lycophyte taxa need to be assessed according to the criteria of the IUCN [86] and the NOM-059 [85], such as regional endemics and those species limited to the tropical montane cloud forests, to determine their potential inclusion in the National and International Red Lists.

We suggest that the 19 endemic species be assessed for inclusion in NOM-059, as they are regionally endemic and have few documented instances. None of these species is listed on the Red List, and their traits exceed those considered for some species already included. Candidate species include taxa from Pteridaceae (genus *Notholaena*, with five species) from semiarid zones with xerophytic vegetation in northern SMOR.

The *Phlegmariurus* species (Lycopodiaceae) are notable and are not necessarily restricted to SMOR. [105] proposed that all Mexican *Phlegmariurus* species be regarded as

threatened or endangered. These species inhabit undisturbed areas of mature pine-oak and humid montane forests, often represented by only a few individuals at each site [106]. The NOM-059 includes only one species (*P. dichotomus*), categorized as threatened. The genus *Phlegmariurus* in Mexico comprises 13 species, seven of which are found in SMOR. These lycophytes are rare or absent in cleared areas because they are epiphytes of mature host canopy trees; they are located in limited localities and collections [106], such as *P. linifolius* (5 records), *P. pringlei* (5 ~~records~~), *P. pithyoides* (3 ~~records~~), and *P. orizabae*, documented from only a single specimen collected in Puebla.

The family Cyatheaceae is a threatened component in the SMOR. The ~~mountain~~ regions of the Neotropics host most of the species richness of American tree ferns [71]. These vascular plants are critically endangered, facing threats from deforestation, exploitation, and global climate change [71, 107, 108]. Most are listed in the NOM-059, but their risk categories need reassessment and updating [71, 109]. Unfortunately, deforestation and land-use change in the SMOR have reduced tropical mountain forest areas due to agriculture and livestock raising [17, 109], threatening the survival of these ferns. The extraction of tree fern stems for handicrafts and as growing medium or substratum for orchids and other plants poses a serious threat to these ferns [107, 108], and some initiatives have been proposed within the SMOR (e.g., [110]). According to Appendix II of CITES, tree ferns are not necessarily at risk of extinction but could become threatened if their trade is not properly regulated. Therefore, the Mexican government must establish trade regulations for Cyatheaceae species to prevent their exploitation and use that threaten their survival.

Conclusions

In the SMOR, [18] recorded 8,472 vascular plant species, suggesting that this mountain range has greater floristic richness than any other mountainous region in Mexico. Conversely, [19] documented 6,981 vascular plants in the SMOR, including 356 species of ferns and lycophytes; [18] identified at least 104 as ‘characteristic’ species of the SMOR without listing the total number of taxa. Our findings include 211 species of ~~pteridoflora~~, exceeding those reported by [19], and thus increasing the overall number of vascular plants in the SMOR.

Herbarium data are crucial for understanding plant biogeography; however, sampling biases and coverage gaps are common in biogeographic studies [104]. Our analysis, mainly based on herbarium specimens, is therefore influenced by these biases. Some areas within the SMOR boundaries lack records of ferns or lycophytes, and 13 grid cells contain only one species. These low-diversity areas may be related to unexplored zones and insufficient sampling, as observed in many sectors of Mexico [67, 69] and the Neotropics [1, 111]. Widespread species are more frequently collected than rare or microendemic species. Future collection efforts in poorly sampled areas of the SMOR are essential to enhance the fern and lycophyte inventory, emphasising the importance and ongoing need for fieldwork [1].

This study provides a detailed inventory of ferns and lycophytes from one of the world's most biodiverse regions, recognized as part of a biodiversity hotspot [93]. This mountain range serves as a transition zone between different biogeographic regions (Neotropical and Nearctic), with diverse climatic, edaphic, and geological conditions and variations in latitude and elevation. This results in high environmental heterogeneity,

leading to significant plant diversity [18]. Based on taxonomic dominance, the most prominent environments are the subhumid temperate (*Pleopeltis* zone) in the middle and high mountains, followed by the semiarid (*Notholaena* zone) on the northern and western slopes, the warm humid (*Adiantum* zone) on the eastern slope, and humid temperate (*Hymenophyllum* zone) in the southern part of the SMOR.

Our findings show that the greatest diversity of ferns and lycophytes is found in the southwestern part of the SMOr, corresponding to the northern mountains of Puebla. This is confirmed by climate data: the southern part of the SMOR receives the most rainfall (>1,200 mm, up to 4,000 mm in Cuetzalan, Puebla) and has the warmest temperatures (between 22 and 26 °C) [28]. Both climate conditions support the presence and growth of many fern and lycophyte species.

Another distinct region is the central part of the SMOR in the state of Tamaulipas (grid cells AY, BB, and BC), which includes the El Cielo Biosphere Reserve and the Sierra de San Carlos. The climate in this section of the SMOR, covering Tamaulipas, features temperate conditions (12-18 °C), with rainfall ranging from 600 to 1,200 mm [28], supporting species with Nearctic affinities. Due to the unique biology of lycophytes and ferns, involving two different phases in their life cycle- one sensitive to humidity [4, 5, 6] and their stomata's low reactivity [7, 8, 9], it is a taxocenosis that functions as an indicator of local environmental conditions. Appropriate vegetation cover and moisture enhance the richness and abundance of pteridoflora. As a result, the distribution patterns observed in this study can serve as a foundation for predicting future environmental impacts, whether from deforestation or climate change.

Note: In this study, we excluded *Microsorum grossum* (Langsd. & Fisch.) S.B. Andrews, an exotic species growing as an epiphyte in nearby zones of Gómez Farías, Tamaulipas [111]. At the final stage of this research, *Adiantum wilesianum* Hook. was recorded in three localities, two from San Luis Potosí and one from Hidalgo, which are not represented in our analyses.

Contributor roles

JDTD: conceptualization, data curation, writing – original draft, writing – review & editing. PVRS: conceptualization, data curation, writing – original draft. RCM: formal analysis, investigation, methodology, writing – original draft, writing – review & editing. JCRM: formal analysis, investigation, methodology. JDA: supervision, validation. LJGM: data curation. CSF: formal analysis, methodology; ILV: conceptualization, formal analysis, funding acquisition, investigation, methodology, project administration, resources, supervision, writing – original draft; writing – review & editing.

Acknowledgements

We are grateful to the curators and staff of the herbaria mentioned in the text for their courtesy during our review of specimens. The HUAP, through Amparo Cerón, helped develop a database from northern Puebla. The IEB, through Brenda Bedolla, helped develop the database from northeastern Querétaro related to the SMOR. CONABIO, through Susana Ocegueda, helped develop the database from Veracruz. Jonathan D. Amith,

co-author, contributed to this research effort as director of a long-term floristic and ethnobotanical project in the Sierra Nororiental de Puebla, during which > 600 ferns and allies were collected, principally in the municipalities of Cuetzalan del Progreso (182), Zacapoaxtla (157) Zautla (72), Zoquiapan (58), and Zongozotla (41). Fieldwork teams were led by Canek Ledesma Corral and Miriam Jiménez Chimil and included Ceferino Salgado Castañeda, Mariano Gorostiza Salazar, Eleuterio Gorostiza Salazar, and Osbel López Francisco.

References

1. Almeida TE, Salino A. State of the art and perspectives on Neotropical fern and lycophyte systematics. J Syst Evol. 2016;54(6):679–690. doi: 10.1111/jse.12223
2. Kessler M. Biogeography of ferns. In: Mehltreter K, Walker LR, Sharpe JM, editors. Fern ecology. Cambridge (UK): Cambridge University Press; 2010. p. 22–60. doi:10.1017/CBO9780511844898.003
3. Suissa JS, Sundue MA, Testo WL. Mountains, climate and niche heterogeneity explain global patterns of fern diversity. J Biogeogr. 2021;48:1296–308. doi:10.1111/jbi.14076
4. Hernández-Álvarez AG, Sánchez-González A, Tejero-Díez JD. Licofitas y helechos del bosque mesófilo de montaña del estado de Hidalgo. Bot Sci. 2019;97:236–49. doi:10.17129/botsci.2093
5. Tejero-Díez JD, Torres-Díaz AN, Gual-Díaz M. Licopodios y helechos en el bosque mesófilo de montaña de México. In: Gual-Díaz M, Rendón-Correa A, editors. Bosques mesófilos de montaña de México: diversidad, ecología y manejo. México: Comisión Nacional para el Conocimiento y Uso de la Biodiversidad; 2014.
6. Tejero-Díez JD, Torres-Díaz AN, Sánchez-González A. Helechos de la Sierra Madre del Sur. In: Luna-Vega I, Espinosa D, Contreras-Medina R, editors. Biodiversidad de la Sierra Madre del Sur: una síntesis preliminar. Ciudad de México: Universidad Nacional Autónoma de México; 2016. pp. 121–55.

7. Brodribb TJ, Holbrook NM. Stomatal protection against hydraulic failure: a comparison of coexisting ferns and angiosperms. New Phytol. 2004;162(3):663–670. doi:10.1111/j.1469-8137.2004.01060.x
8. Brodribb TJ, McAdam SAM, Jordan GJ, Feild TS. Evolution of stomatal responsiveness to CO_2 and optimization of water-use efficiency among land plants. New Phytol. 2009;183(3):839–847. doi:10.1111/j.1469-8137.2009.02844.x
9. Brodribb TJ, McAdam SAM. Passive origins of stomatal control in vascular plants. Science. 2011;331(6017):582–585. doi:10.1126/science.11979
10. Silva MM, Farias RP, Costa LEN, Barros ICLA. Leaf phenological traits of the tree fern *Cyathea praecincta* (Cyatheaceae) in a Brazilian lowland tropical forest. Aust J Bot. 2018;66(8):618–27. doi:10.1071/BT18084
11. Yang J, Wang D, Liu L, Zhou Y. Fernlike plants establishing the understory of the Late Devonian Xinhang lycopsid forest. Life. 2024;14(5):602. doi:10.3390/life14050602
12. Hassler M. World ferns. Synonymic checklist and distribution of ferns and lycophytes of the world. Version 19.3. 1994–2024 [cited 2024 Jun 18].
13. Villaseñor JL. Checklist of the native vascular plants of Mexico. Revista Mexicana de Biodiversidad. 2016 Sep;87(3):559–902. doi:10.1016/j.rmb.2016.06.017.
14. Tejero-Díez JD, Contreras-Medina R, Torres-Díaz AN, González-Elizondo MS, Sánchez-González A, Luna-Vega I. Diversity of the Pteridoflora of montane northwestern Mexico. Diversity. 2023;15:324. doi:10.3390/d15030324
15. Mickel JT, Smith AR. The pteridophytes of Mexico. Mem NY Bot Gard. 2004;88:1–1054.
16. Luna-Vega I, Alcántara-Ayala O, Contreras-Medina R. Patterns of diversity, endemism and conservation: an example with Mexican species of Ternstroemiaceae Mirb. ex DC. In: Luna-Vega I, Morrone JJ, Espinosa D, editors. Biodiversidad de la Sierra Madre Oriental. Ciudad de México: Universidad Nacional Autónoma de México CONABIO; 2004. pp. 2723–2739.
17. Rzedowski J. Catálogo preliminar de plantas vasculares de distribución restringida a la Sierra Madre Oriental. Flora del Bajío y de regiones adyacentes. Fascículo complementario XXXI; 2015. 36 p.

18. Villaseñor JL, Ortiz E. A phytogeographic assessment of the Sierra Madre Oriental physiographic province, Mexico. Bot Sci. 2022;100(4):1102–23. doi:10.17129/botsci.3086
19. Salinas-Rodríguez MM, Hernández-Sandoval L, Carrillo-Reyes P, Castillo-Gómez HA, Castro-Castro A, Estrada-Castillón E, et al. Diversidad de plantas vasculares de la provincia fisiográfica de la Sierra Madre Oriental, México. Bot Sci. 2022;100(2):469–92. doi:10.17129/botsci.2864
20. Sanginés-Franco C, Luna-Vega I, Alcántara-Ayala O, Contreras-Medina R. Distributional patterns and biogeographic analysis of ferns in the Sierra Madre Oriental, Mexico. Am Fern J. 2011;101(2):81–104. doi:10.1640/0002-8444-101.2.81
21. Hernández-Cárdenas RA, Serrato-Díaz A, López-Ferrari AR, Espejo-Serna A. Novelties in the genus *Viridantha* Espejo (Tillandsioideae, Bromeliaceae). PhytoKeys. 2019;132:99–110.
22. Smith AR. Pteridophytes. In: Breedlove DE, editor. Flora of Chiapas. Part 2. San Francisco: California Academy of Sciences; 1981. pp. 1–370.
23. Pteridophyte Phylogeny Group (PPG I). A community-derived classification for extant lycophytes and ferns. J Syst Evol. 2016;54:563–603. doi:10.1111/jse.12229
24. Morrone JJ. Neotropical biogeography: regionalization and evolution. Boca Raton (FL): CRC Press; 2017. doi:10.1201/b21824
25. Eguiluz de Antuñano S, Aranda-García M, Marrett R. Tectónica de la Sierra Madre Oriental, México. Bol Soc Geol Mex. 2000;53:1–26.
26. INEGI. Fisiografía. Conjunto de datos vectoriales Fisiográficos. Continuo Nacional. escala 1:1 000 000. Serie I. Aguascalientes, Mexico: Instituto Nacional de Estadística y Geografía; 2001. [cited 2025 Dec] Available from: https://www.inegi.org.mx/temas/fisiografia/
27. Santa Anna del Conde H, Contreras-Medina R, Luna-Vega I. Biogeographic analysis of endemic cacti of the Sierra Madre Oriental, Mexico. Biol J Linn Soc. 2009;97(2):373–89. doi:10.1111/j.1095-8312.2009.01212.x
28. Hernández-Cerda ME, Carrasco-Anaya G. Climatología. In: Luna-Vega I, Morrone JJ, Espinosa D, editors. Biodiversidad de la Sierra Madre Oriental. Mexico City: Universidad Nacional Autónoma de México; 2004. pp. 63–108.

29. Álvarez-Zúñiga E, Sánchez-González A, López-Mata L, Tejero-Díez JD. Composición y abundancia de las pteridofitas en el bosque mesófilo de montaña del municipio de Tlanchinol, Hidalgo, México. Bot Sci. 2012;90(2):163–177.
30. Andrés-García MF. Efecto de la perturbación sobre la pteridoflora de un bosque mesófilo de montaña en la Sierra Norte de Puebla [tesis de licenciatura]. Ciudad de México: FES-Iztacala, UNAM; 2022.
31. Cartujano S, Zamudio S, Alcántara O, Luna-Vega I. El bosque mesófilo de montaña en el municipio de Landa de Matamoros, Querétaro, México. Bol Soc Bot Mex. 2002;70:13–43.
32. Cerón-Carpio AB, Arreguín-Sánchez ML, Fernández-Nava R. Listado con anotaciones de las pteridofitas del municipio de Tlatlauquitepec, Puebla, México y su distribución de las especies en los diferentes tipos de vegetación. Polibotánica. 2006;21:45–60.
33. Cerón-Carpio AB, Rojas-Alvarado AF, Tejero-Díez JD, Caamaño-Onofre L. Los híbridos de *Pleopeltis* (Polypodiaceae, Polypodiophyta) en México: novedades y precisiones. Acta Bot Mex. 2017;119:101–14. doi:10.21829/abm119.2017.1234
34. Clausen RT. Ferns of the Monterrey Region, Mexico. Am Fern J. 1950;40(1):89–97. doi:10.2307/1544950
35. García-Morales LJ, Estrada-Castillón AE, García-Jiménez J, Villarreal-Quintanilla JA, Cantú-Ayala C, Jurado-Ybarra E, et al. Florística y vegetación del Área Natural Protegida Altas Cumbres, Tamaulipas, México. In: Barrientos-Lozano L, Correa Sandoval A, Horta Vega JV, García Jiménez J, editors. Biodiversidad de Tamaulipas. Vol. 2. Cd. Victoria: Dirección General de Educación Superior, Instituto Tecnológico de Ciudad Victoria; 2014.
36. Gómez-Noguez F, Mendoza-Ruiz A, Pérez-García A. Nota sobre *Onocleopsis hintonii* (Onocleaceae), un nuevo registro para el estado de Hidalgo (México). Acta Bot Mex. 2013;102:31–7. doi:10.21829/abm102.2013.23
37. Grajales-Tam KM. Azollaceae. Flora del Bajío y de regiones adyacentes, INECOL. 2014;185:1–7. doi:10.21829/fb.60.2014.185
38. Gutiérrez-Lozano M, Sánchez-González A, López-Mata L, Tejero-Díez JD. Taxonomic richness of lycophytes and ferns of the Mexican beech forest: Highest

ever recorded among *Fagus* forests worldwide? Flora. 2017;229:23–31. doi:10.1016/j.flora.2017.02.008

39. Guzmán-Cornejo L, Sánchez-Morales A, Pacheco L. Helechos y licofitas del municipio de Cardonal, Hidalgo, México. Rev Mex Biodivers. 2015;86(3):573–81. doi:10.1016/j.rmb.2015.06.004

40. Hernández-Mendoza AM, Arreguín-Sánchez ML, García-Jiménez J, Herrera-Monsivais MC. Composición taxonómica y datos ecológicos de las licofitas y monilofitas de la Sierra de Tamaulipas, Tamaulipas, México. Polibotánica. 2015;40:29–44.

41. Hernández-Rojas AC, Kluge J, Krömer T, Carvajal-Hernández C, Silva-Mijangos L, Miehe G, et al. Latitudinal patterns of species richness and range size of ferns along elevational gradients at the transition from tropics to subtropics. J Biogeogr. 2020;47(6):1383–97. doi:10.1111/jbi.13841

42. Juárez-Medina AK. Biodiversidad de la flora del bosque mesófilo de montaña del municipio de Huayacocotla, Veracruz, México [Bachelor dissertation]. Mexico City: Facultad de Ciencias, Universidad Nacional Autónoma de México; 2008.

43. Krömer T, Carvajal-Hernández CI, Acebey AR. A decade of new Pteridophyte records for the state of Veracruz, Mexico. Am Fern J. 2015;105(3):162–75.

44. Martínez-Cabrera D, Hernández-Hernández NN, Isidro-Hernández B, Hernández-Álvarez AG, Sánchez-González A. Diversidad de licopodios y helechos del bosque tropical subcaducifolio del estado de Hidalgo, México. Acta Bot Mex. 2019;126:e1434. doi:10.21829/abm126.2019.1434

45. Mendoza A, Windham M, Pérez-García B, Yatskievych G. Una nueva especie de *Pellaea* (Pteridaceae) del estado de San Luis Potosí, México. Acta Bot Mex. 2001;57:15–21. doi:10.21829/abm57.2001.881

46. Mendoza-Ruiz A, Ceja-Romero J. Nuevos registros de licofitas y helechos para el estado de Puebla, México. Acta Bot Mex. 2020;127:e1637. doi:10.21829/abm127.2020.1637

47. Moran RC, Prado J. *Megalastrum* (Dryopteridaceae) in Central America. Kew Bull. 2010;65(2):137–88. doi:10.1007/s12225-010-9214-3

48. Mora-Olivo A, Mendoza-Ruiz A, Martínez-Ávalos JG. *Isoetes tamaulipana* (Isoetaceae), a new species from Mexico. Phytotaxa. 2016;267(2):113–20. doi:10.11646/phytotaxa.267.2.3
49. Palacios-Ríos M. Las pteridofitas del estado de Veracruz, México [Master's thesis]. Mexico City: Facultad de Ciencias, Universidad Nacional Autónoma de México; 1992.
50. Pérez-Atilano Y, Sánchez-González A, Tejero-Díez JD. Species richness, distribution, and morphological variation of lycophytes and monilophytes in a semi-arid region of Mexico. Am Fern J. 2015;105(3):238–56. doi:10.1640/0002-8444-105.3.238
51. Pérez-Paredes MG, Sánchez-González A, Tejero-Díez JD. Listado de licopodios y helechos del municipio de Zacualtipán de Ángeles, Hidalgo, México. Polibotánica. 2012;33:57–73.
52. Ramírez-Cruz S, Sánchez-González A, Tejero-Díez D. La pteridoflora del Parque Nacional Los Mármoles, Hidalgo, México. Bol Soc Bot Mex. 2009;84:35–44. doi:10.17129/botsci.2293
53. Rojas-Alvarado AF. Notes on *Elaphoglossum* (Lomariopsidaceae) section *Polytrichia* subsection *Hybrida* in Mexico and Central America. Rev Biol Trop. 2003;51(1):33–48.
54. Romero-Santamaría A. Diversidad y estructura de especies leñosas en un fragmento de bosque mesófilo de montaña en Xicotepec, Puebla, México [Bachelor dissertation]. Mexico City: FES-Iztacala, Universidad Nacional Autónoma de México; 2017.
55. Sánchez-González A, Álvarez-Zúñiga E, Tejero-Díez JD. Richness and distribution patterns of ferns and lycopods in Los Mármoles National Park, Hidalgo, Mexico. J Torrey Bot Soc. 2010;137(4):373–9. doi:10.3159/10-RA-002.1
56. Tejero-Díez JD, Pacheco L. Notas taxonómicas y de distribución en *Polypodium plesiosorum* y *P. rhodopleuron* (Polypodiaceae: Pteridophyta). An Inst Biol Univ Nac Autón Méx Ser Bot. 2004;75(1):11–37.

57. Tejero-Díez JD, Pacheco L. Taxa nuevos, nomenclatura, redefinición y distribución de las especies relacionadas con *Polypodium colpodes* Kunze (Polypodiaceae: Pteridophyta). Acta Bot Mex. 2004;67:75–115. doi:10.21829/abm67.2004.976

58. Torres-Colín R, Parra JG, de la Cruz LA, Ramírez MP, Gómez-Hinostrosa C, Bárcenas RT, Hernández HM. Flora vascular del municipio de Guadalcázar y zonas adyacentes, San Luis Potosí, México. Rev Mex Biodivers. 2017;88(3):524–54. doi:10.1016/j.rmb.2017.07.003

59. Tzompa-Coatl R, Cerón-Carpio AB, Mendoza-Ruiz A, Ceja-Romero J. Riqueza específica y distribución de licofitas y helechos por tipos de vegetación, en la Sierra Norte de Puebla, México. Acta Bot Mex. 2022;129:e2063. doi:10.21829/abm129.2022.2063

60. Velazco-Macías CG, Salcedo-Martínez SM, Alanís-Flores GJ, González-Álvarez M, Alvarado-Vázquez MA. Helechos de Nuevo León. Una guía ilustrada. Monterrey: Universidad Autónoma de Nuevo León; 2012.

61. Velázquez-Montes E. Pteridaceae. Flora del Bajío y de Regiones Adyacentes, INECOL. 2019;210:1–254. doi:10.21829/fb.40.2019.210

62. Thiers B. Index Herbariorum: a global directory of public herbaria and associated staff [Internet]. New York: New York Botanical Garden's Virtual Herbarium; [cited 2024 Nov 3].

63. Environmental Systems Research Institute (Esri). ArcGIS Pro. Version 3.0.1. Redlands (CA): Esri; 2022.

64. Turland NJ, Wiersema JH, Barrie FR, Greuter W, Hawksworth DL, Herendeen, Pb et al., editors. International Code of Nomenclature for algae, fungi, and plants (Shenzhen Code) adopted by the Nineteenth International Botanical Congress Shenzhen, China, July 2017. Regnum Vegetabile 159. Glashütten: Koeltz Botanical Books; 2018. doi:10.12705/Code.2018

65. Zhou XM, Zhang LB. Phylogeny, character evolution, and classification of Selaginellaceae (lycophytes). Plant Divers. 2023;45:630–84. doi:10.1016/j.pld.2023.07.003

66. Ortiz-Bermúdez, E. Nomenclator de localidades. In: Biodiversidad de la Sierra Madre Oriental, Luna-Vega I, Morrone JJ, Espinosa D, editors. Mexico City: UNAM-CONABIO; 2004. pp. 25-62.

67. Rodríguez A, Castro-Castro A, Vargas-Amado G, Vargas-Ponce O, Zamora-Tavares P, González-Gallegos J, et al. Richness, geographic distribution patterns, and areas of endemism of selected angiosperm groups in México. J Syst Evol. 2018;56(5):537–49. doi:10.1111/jse.12457

68. Villaseñor JL, Ortiz E, Juárez D. Transition zones and biogeographic characterization of endemism in three biogeographic provinces of central Mexico. Bot Sci. 2021;99:938–54. doi:10.17129/botsci.2768

69. Contreras-Medina R, García-Martínez AI, Ramírez-Martínez JC, Espinosa D, Balam-Narváez R, Luna-Vega I. Biogeographic analysis of ferns and lycophytes in Oaxaca: a Mexican beta-diverse area. Bot Sci. 2022;100:204–22. doi:10.17129/botsci.2844

70. Colwell RK. EstimateS: Statistical estimation of species richness and shared species from samples. Version 9.1. User's Guide and application published at: http://purl.oclc.org/estimates; 2009.

71. Ramírez-Barahona S, Luna-Vega I, Tejero-Díez JD. Species richness, endemism and conservation of American tree ferns (Cyatheales). Biodivers Conserv [Internet]. 2011;20:59–72. doi:10.1007/s10531-010-9946-2.

72. Sanginés-Franco C, Luna-Vega I, Contreras-Medina R, Espinosa D, Tejero-Díez JD, Rivas G. Diversity, endemism and conservation of ferns (Polypodiales) in the Mexican Mountain Component. J Mt Sci. 2015;12:891–904. doi:10.1007/s11629-014-3070-9

73. Crisp MD, Laffan SW, Linder HP, Monro A. Endemism in the Australian flora. J Biogeogr 2001 Feb;28(2):183–98.

74. Linder HP. Plant diversity and endemism in subSaharan tropical Africa. J Biogeogr 2001 Feb;28(2):169–82. doi:10.1046/j.1365-2699.2001.00527.x

75. Laffan SW, Lubarsky E, Rosauer DF. Biodiverse, a tool for the spatial analysis of biological and related diversity. Ecography. 2010;33(4):643–47. doi:10.1111/j.1600-0587.2010.06237.xC

76. Aagesen L, Szumik C, Goloboff P. Consensus in the search for areas of endemism. J Biogeogr. 2013;40:2011–2016. doi:10.1111/jbi.12172
77. Morrone JJ. Evolutionary biogeography: An integrative approach with case studies. New York: Columbia University Press; 2009. doi:10.1093/sysbio/syq016
78. Vaghasiya PM, Dhamsaniya S, Paradva BR, Patel RM. Vegetation characteristics of Chapredi Reserve Forest of Bhuj-Kachchh, Gujarat. J Appl For Ecol. 2015;3(1):65–73.
79. Squeo F, Caviers L, Arancio G, Novoa J, Matthei O, Marticorena C, et al. Biodiversidad de la flora vascular en la Región de Antofagasta, Chile. Rev Chil Hist Nat. 1998;71:571–91.
80. Carvajal-Hernández CI, Krömer T. Riqueza y distribución de helechos y licófitos en el gradiente altitudinal del Cofre de Perote, centro de Veracruz, México. Bot Sci. 2015;93:601–14. doi:10.17129/botsci.165
81. Murillo J, Murillo MT. Diversidad de los helechos y licófitos de Colombia. Acta Bot Malacit. 2017;42:23–32. doi:10.24310/abm.v42i1.2654
82. Salazar L, Homeier J, Kessler M, Abrahamczyk S, Lehnert M, Krömer T, Kluge J. Diversity patterns of ferns along elevational gradients in Andean tropical forests. Plant Ecol Divers. 2013;8:13–24. doi:10.1080/17550874.2013.843036
83. Luna-Vega I, Tejero-Díez D, Contreras-Medina R, Heads M, Rivas G. Biogeographical analysis of two *Polypodium* species complexes (Polypodiaceae) in Mexico and Central America. Biol J Linn Soc. 2012;106:940–55. doi:10.1111/j.1095-8312.2012.01921.x
84. Salinas-Moreno Y, Mendoza MG, Barrios MA, Cisneros R, Macías-Sámano J, Zúñiga G. Areography of the genus *Dendroctonus* (Coleoptera: Curculionidae: Scolytinae) in Mexico. J Biogeogr. 2004;31:1163–77. doi:10.1111/j.1365-2699.2004.01110.x
85. SEMARNAT. Modificación del Anexo Normativo III, Lista de especies en riesgo de la Norma Oficial Mexicana NOM-059-SEMARNAT-2010. Protección ambiental-Especies nativas de México de flora y fauna silvestres-Categorías de riesgo y especificaciones para su inclusión, exclusión o cambio-Lista de especies en riesgo. DOF. 2019. https://www.dof.gob.mx/nota_detalle_popup.php?codigo=5578808

86. IUCN. 2025. The IUCN Red List of Threatened Species. Version 2025-1. Accessed on [18 June 2025]. https://www.iucnredlist.org.

87. CITES. Convention on International Trade in Endangered Species of Wild Fauna and Flora. Appendices I, II and III. 2025. Available from: https://cites.org/eng/app/appendices.php

88. Sánchez-Salas J, Muro G, Estrada-Castillón E, Alba-Ávila JA. El MER: un instrumento para evaluar el riesgo de extinción de especies en México. Rev Chapingo Ser Zonas Áridas. 2013;12:30–5. doi:10.5154/r.rchsza.2012.06.037

89. Chao A. Non-parametric estimation of the number of classes in a population. Scand J Stat 1984; 11: 265–270. https://doi.org/10.2307/4615964

90. Chao A. Estimating the population size for capture-recapture data with unequal catchability. Biometrics 1987;43: 783–791. https://doi.org/10.2307/2531532

91. Villarreal H, Álvarez M, Córdoba S, Escobar F. Manual de métodos para el desarrollo de inventarios de biodiversidad. Programa de Inventarios de Biodiversidad. Bogotá, Colombia: Instituto de Investigación de Recursos Biológicos Alexander von Humboldt. 2006.

92. Hernández-Rojas AC, Kluge J, Noben S, Reyes-Chávez JD, Krömer T, Carvajal-Hernández CI, et al. Phylogenetic diversity of ferns reveals different patterns of niche conservatism and habitat filtering between epiphytic and terrestrial assemblages. Front Biogeogr. 2021;13(3). doi:10.21425/F5FBG50023

93. Suissa JS, Sundue MA. Diversity patterns of Neotropical ferns: revisiting Tryon's centers of richness and endemism. Am Fern J. 2020;110:211–32. doi:10.1640/0002-8444-110.4.211

94. Grajales-Tam KM, Tejero-Díez JD. Pteridaceae I: Subfamilia Cryptogrammoideae. Flora de Veracruz. 2024; fasc. 24. doi:10.21829/fv.620.2024.204

95. Sundue MA, Testo WL, Ranker TA. Morphological innovation, ecological opportunity, and the radiation of a major vascular epiphyte lineage. Evolution. 2015;69:2482–95. doi:10.1111/evo.12749

96. Sessa ES, Zimmer EA, Givnish TJ. Phylogeny, divergence times, and historical biogeography of New World *Dryopteris* (Dryopteridaceae). Am J Bot. 2012;99:730–50. doi:10.3732/ajb.1100294

97. Rouhan G, Dubuisson JY, Rakotondrainibe F, Motley TJ, Mickel JT, Labat JN, Moran RC. Molecular phylogeny of the fern genus *Elaphoglossum* (Elaphoglossaceae) based on chloroplast non-coding DNA sequences: contributions of species from the Indian Ocean area. Mol Phylogenet Evol. 2004;33(3):745–63. doi:10.1016/j.ympev.2004.08.006

98. Alejo-Jacuinde G, Kean-Galeno T, Martínez-Gallardo N, Tejero-Díez D, Mehltreter K, Délano-Frier JP, et al. Viability markers for determination of desiccation tolerance and critical stages during dehydration in *Selaginella* species. J Exp Bot. 2022;73(12):3898–3912. doi:10.1093/jxb/erac121

99. Korall P, Kenrick P. Phylogenetic relationships in Selaginellaceae based on Rbcl sequences. Am J Bot. 2002;89(3):506–17. doi:10.3732/ajb.89.3.506. PMID: 21665649

100. Schneider EL, Carlquist S. SEM studies on the vessels of heterophyllous species of *Selaginella*. J Torrey Bot Soc. 2000;127(4):263–70. doi:10.2307/3088644

101. Morrone JJ. The Mexican Transition Zone: A natural biogeographic laboratory to study biotic assembly. Cham: Springer; 2020. doi:10.1007/978-3-030-47917-6

102. James SA, Soltis PS, Belbin L, Chapman AD, Nelson G, Paul DL, Collins M. Herbarium data: Global biodiversity and societal botanical needs for novel research. Appl. Plant Sci. 2018;6(2):e1024. doi:10.1002/aps3.1024

103. Daru BH, Rodriguez J. Mass production of unvouchered records fails to represent global biodiversity patterns. Nat Ecol Evol, 2023;7:816-831. doi:10.1038/s41559-023-02047-3

104. Daru BH. Tracking hidden dimensions of plant biogeography from herbaria. New Phytol. 2025;246(1):61-77. doi:10.1111/nph.70002

105. Tejero-Díez JD, Torres-Díaz AN, Mickel JT, Mehltreter K, Krömer T. Helechos y licopodios. In: Comisión Nacional para el Conocimiento y Uso de la Biodiversidad (CONABIO). La biodiversidad en Veracruz: estudio de estado. México: CONABIO, Gobierno del Estado de Veracruz, Universidad Veracruzana, Instituto de Ecología A.C. 2011. Vol. II. p. 97–115 + apéndice VIII.6.

106. Armenta-Montero S, Carvajal-Hernández CI, Ellis EA, Krömer T. Distribution and conservation status of *Phlegmariurus* (Lycopodiaceae) in the state of Veracruz, Mexico. Trop Conserv Sci. 2015;8:114–137. doi:10.1177/194008291500800
107. Dadang RJ, Casinillo NG, Coritico FP, Simborio LT, Amoroso VB. Living with endangered species: collection of tree ferns in the forest reserve of Marilog district, southern Philippines. Trees For Peop. 2020;2:100041. doi: 10.1016/j.tfp.2020.100041
108. Luna-Vega I, Alcántara-Ayala O, Contreras-Medina R, Ponce-Vargas A. Biogeography, current knowledge and conservation of threatened vascular plants characteristic of Mexican temperate forests. Biodivers Conserv. 2006;15(12):3773–99. doi:10.1007/s10531-005-5401-1
109. Luna-Vega I, Alcántara-Ayala O, García-Morales LJ, Espinosa D, Ramírez-Martínez JC, Contreras-Medina R. Threatened trees characteristic of Mexican tropical montane cloud forests. Diversity. 2023;15(1):42. doi:10.3390/d15010042
110. Eleutério AA, Pérez-Salicrup D. Management of tree ferns (*Cyathea* spp.) for handicraft production in Cuetzalan, Mexico. Econ Bot. 2006;60(2):182–6. doi:0.1663/0013-0001(2006)60[182:MOTFCS]2.0.CO;2
111. Tejero-Díez J D, Torres-Díaz AN. *Phymatosorus grossus* (Polypodiaceae) en México y comentarios sobre otros pteridobiontes no-nativos. Acta Bot Mex. 2012;98:111–124. doi:10.21829/abm98.2012.1143

Figure 3

Click here to access/download;Figure;Figure 3.tif

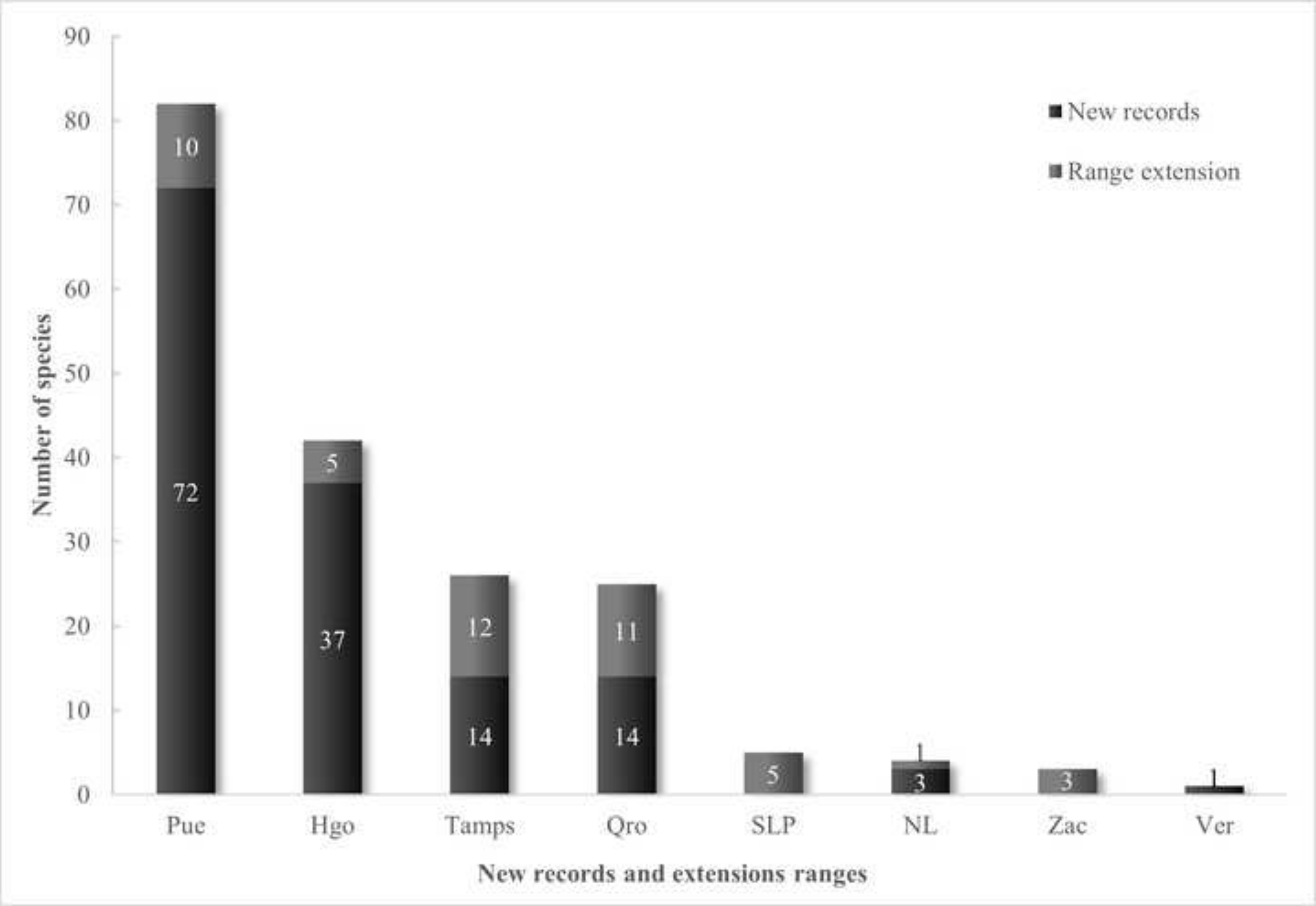

Figure 4

Click here to access/download;Figure;Figure 4.tif

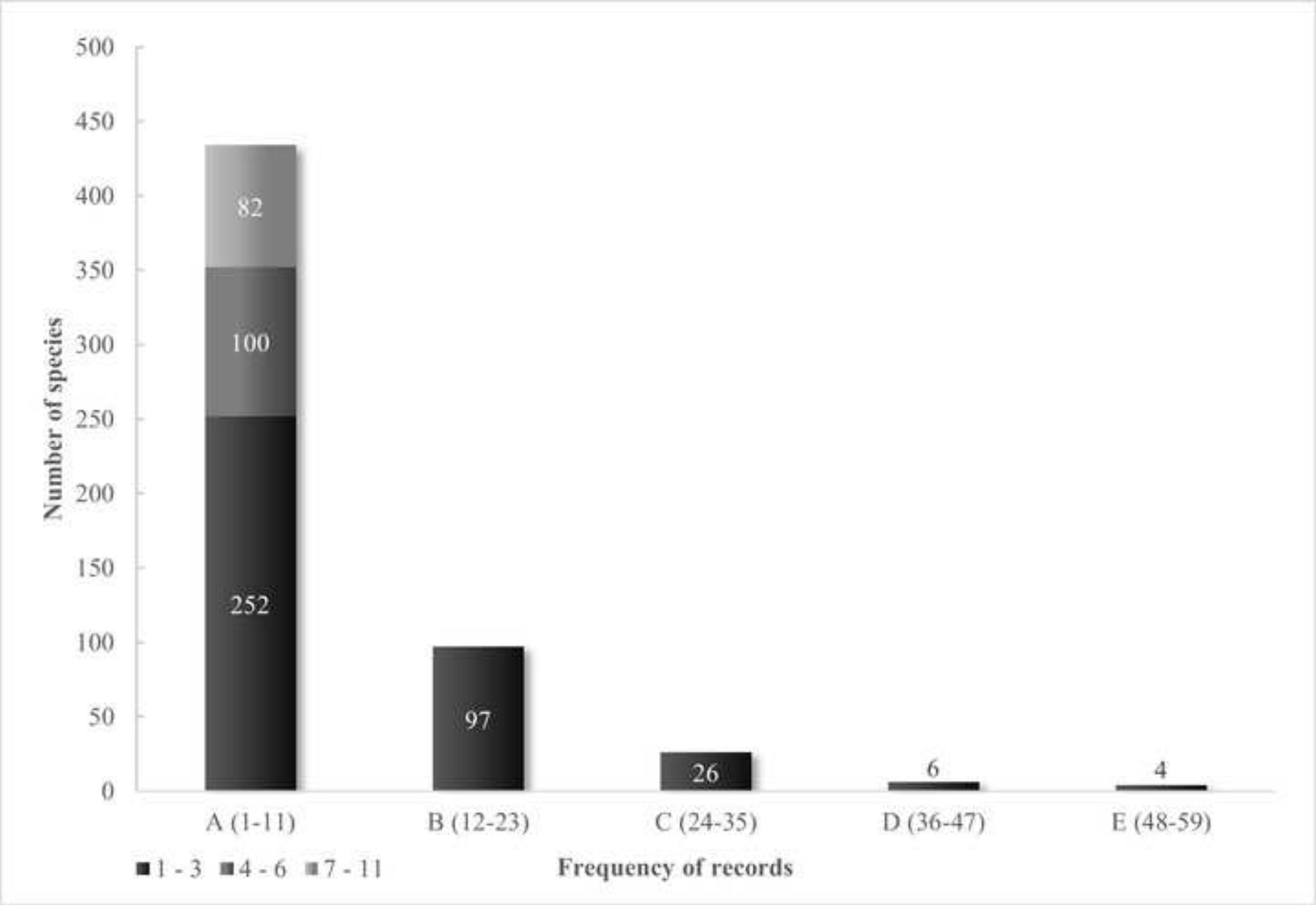

Figure 2

Click here to access/download;Figure;Figure 2 new.tif

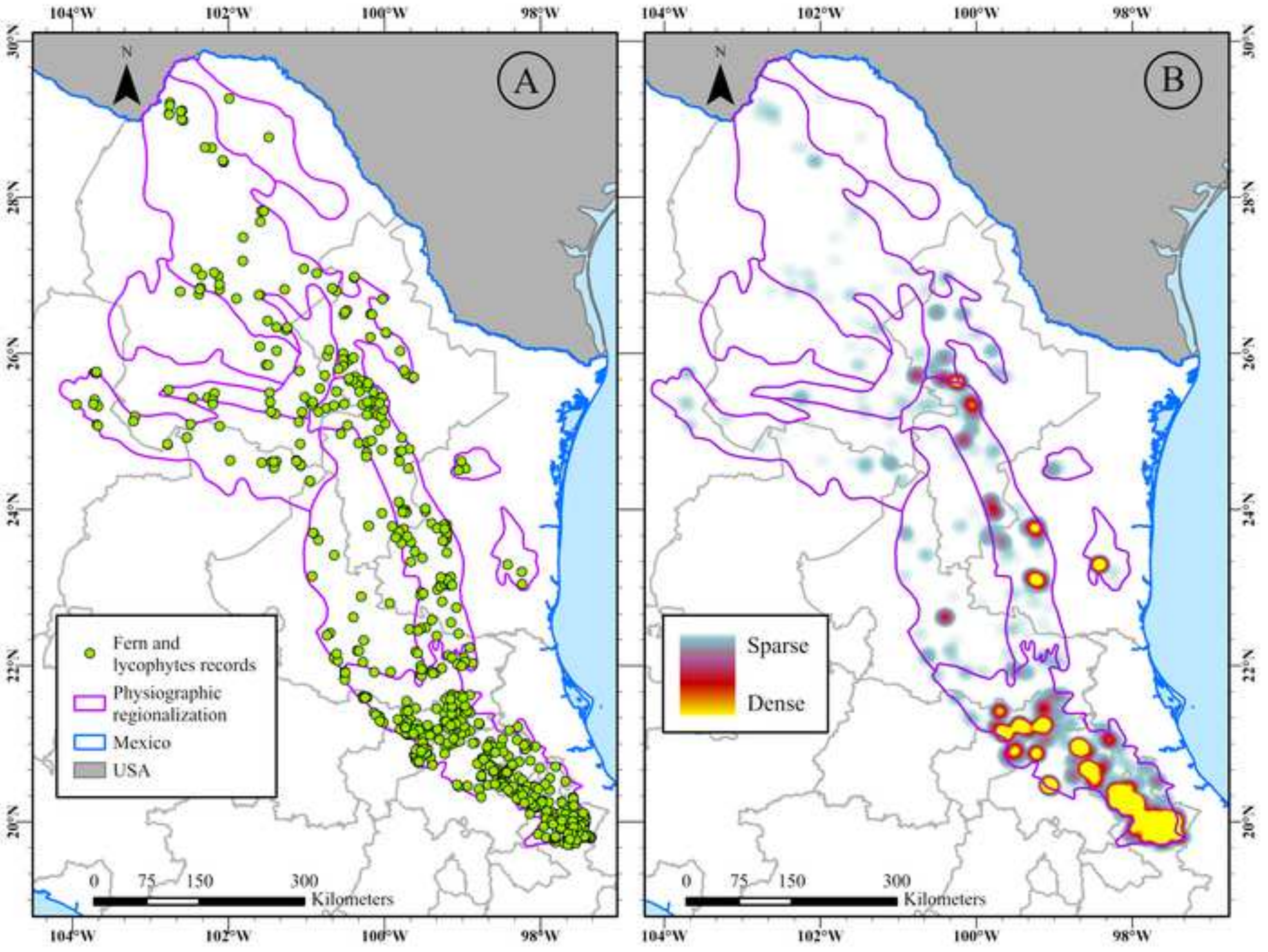

Figure 5

Click here to access/download;Figure;Figure 5 new.tif

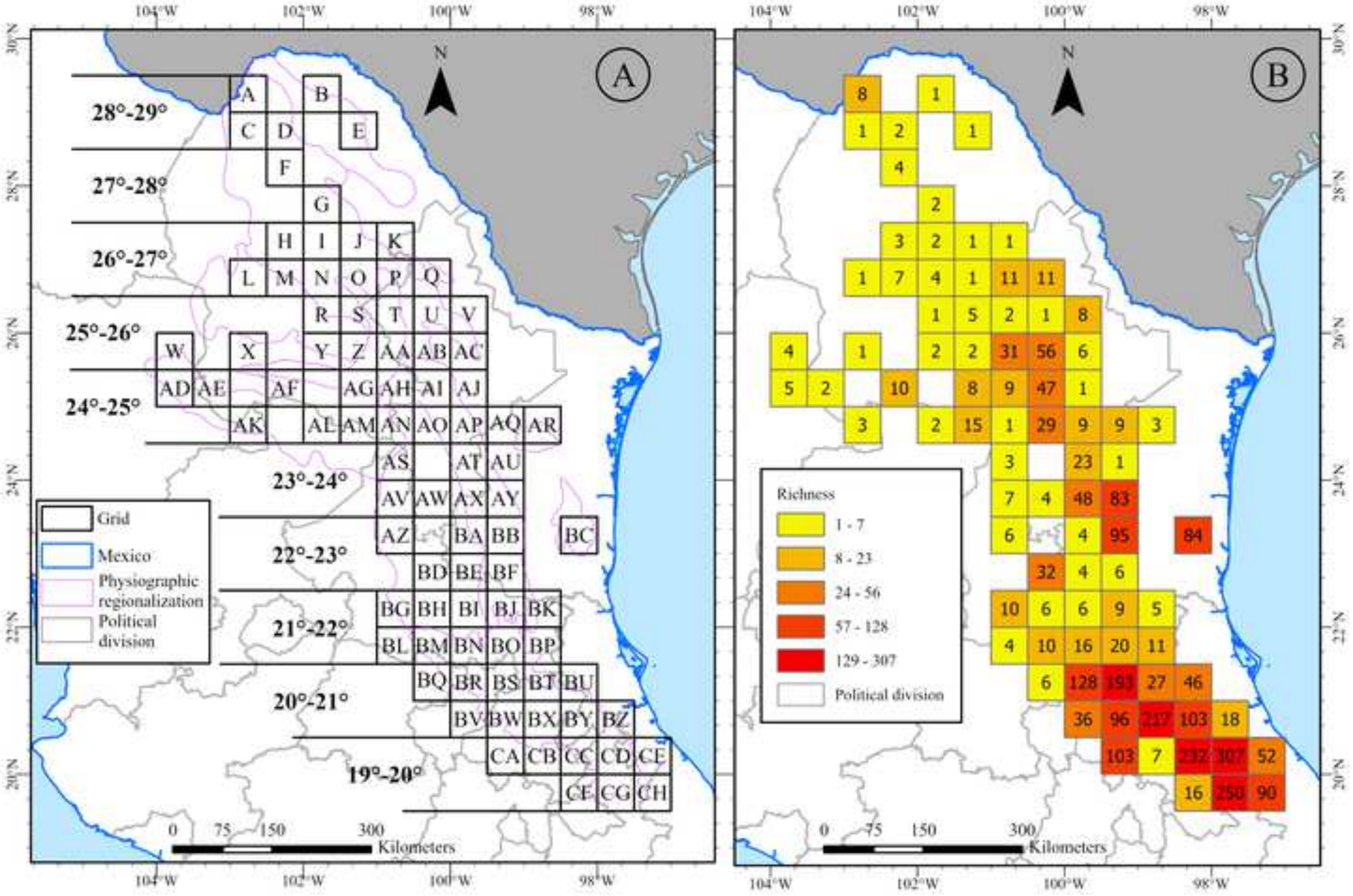

Figure 6

Click here to access/download;Figure;Figure 6.tif

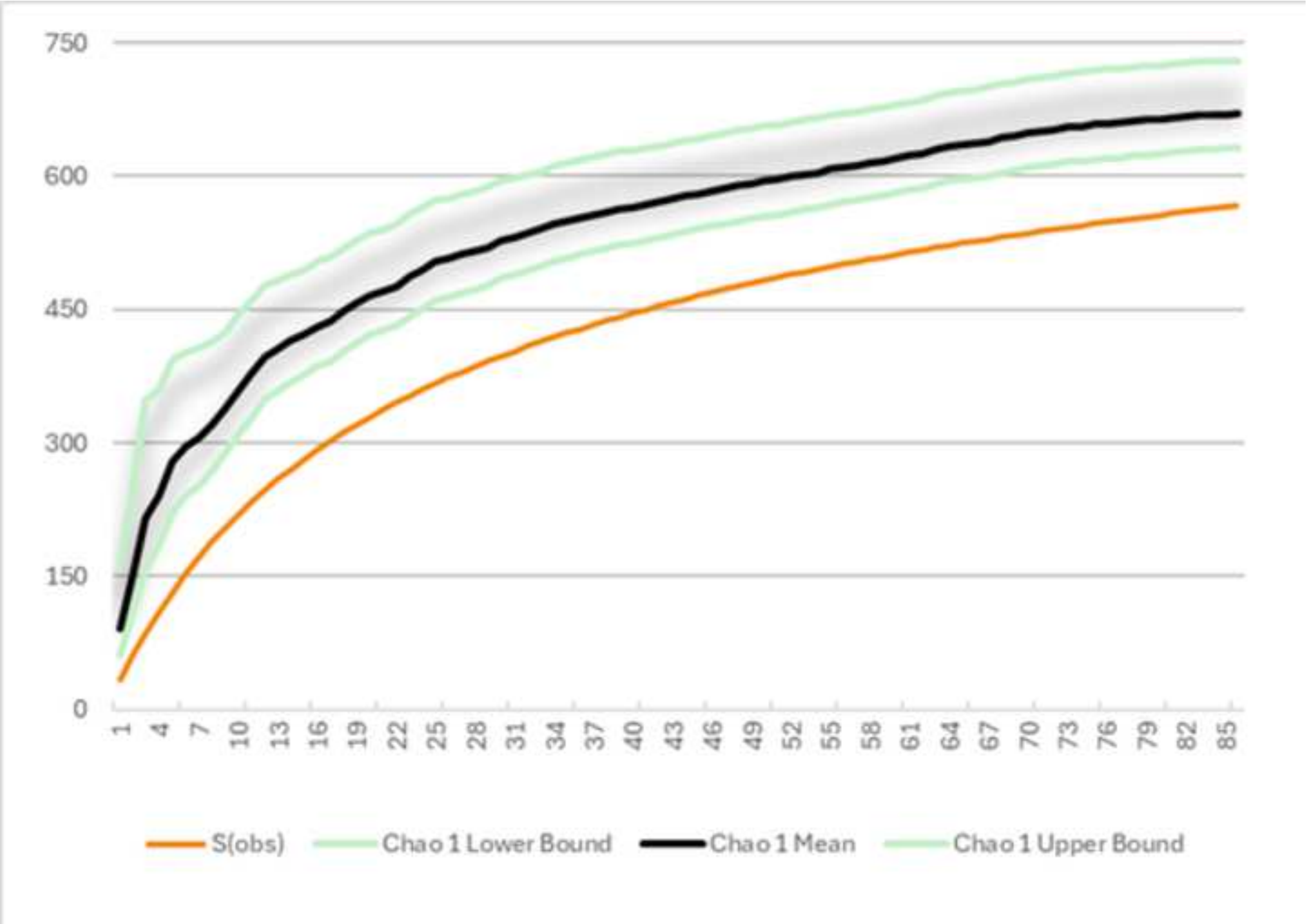

Figure 7

Click here to access/download;Figure;Figure 7 new.tif

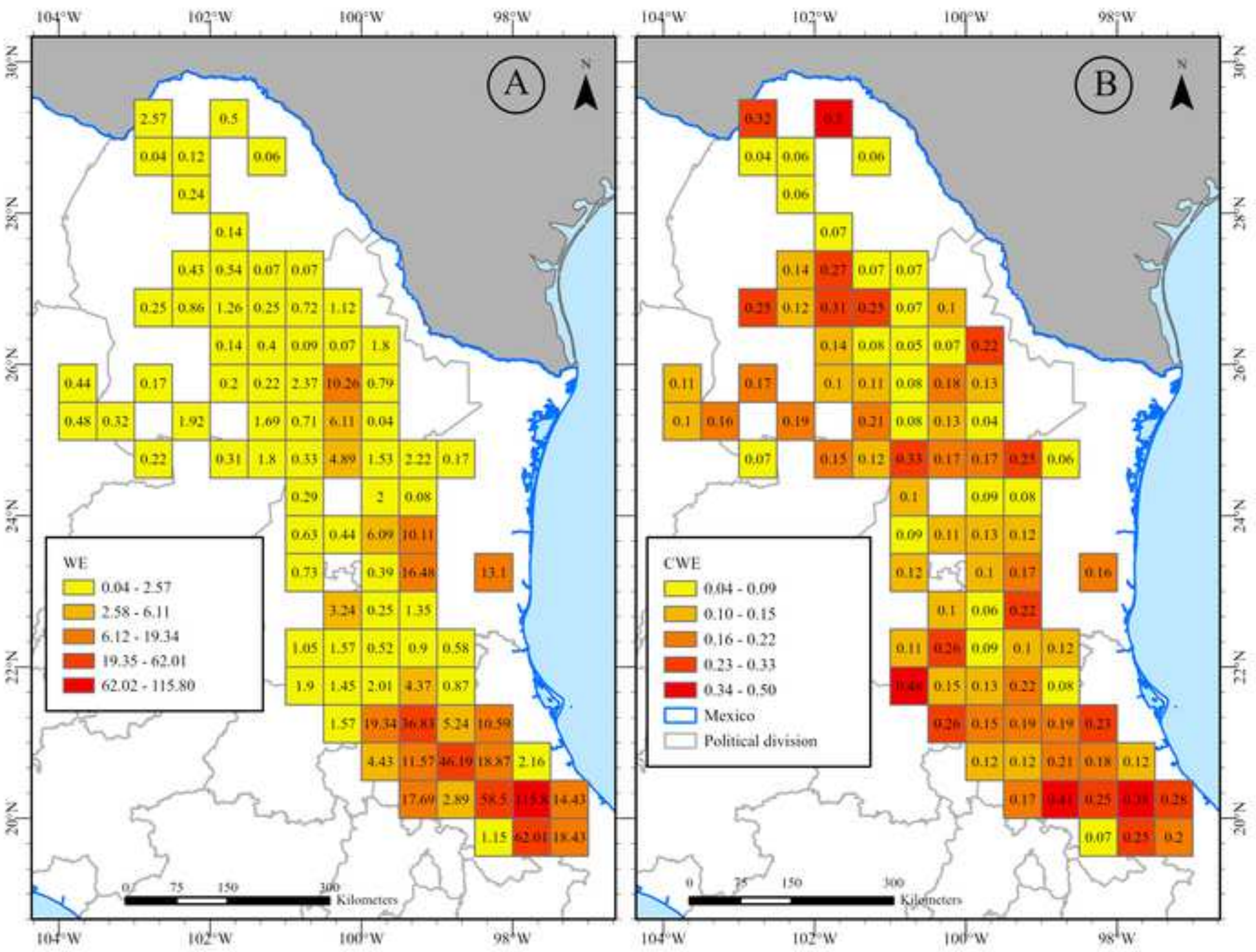

Figure 8

Click here to access/download;Figure;Figure 8.tif

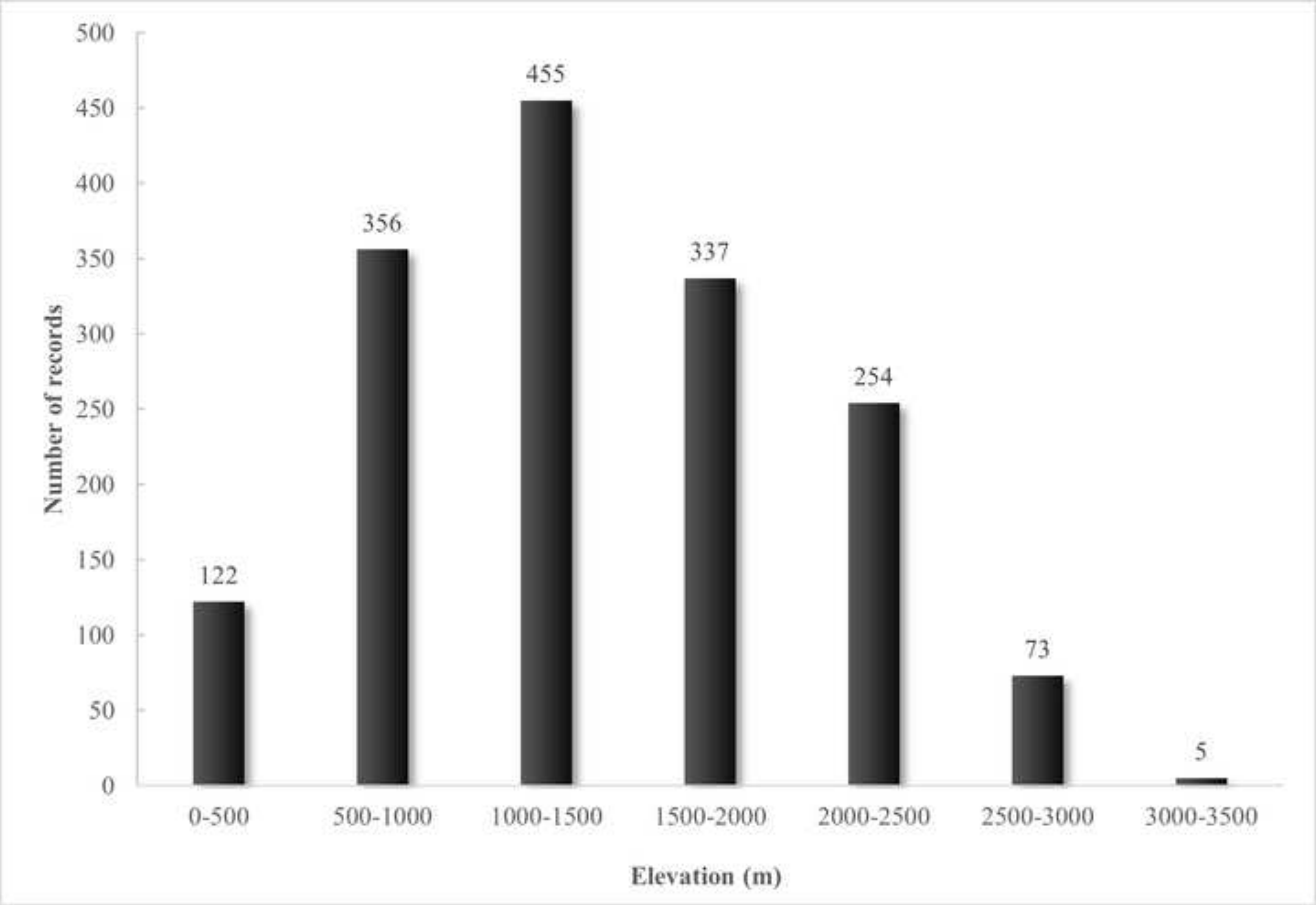

Figure 9

Click here to access/download;Figure;Figure 9.tif

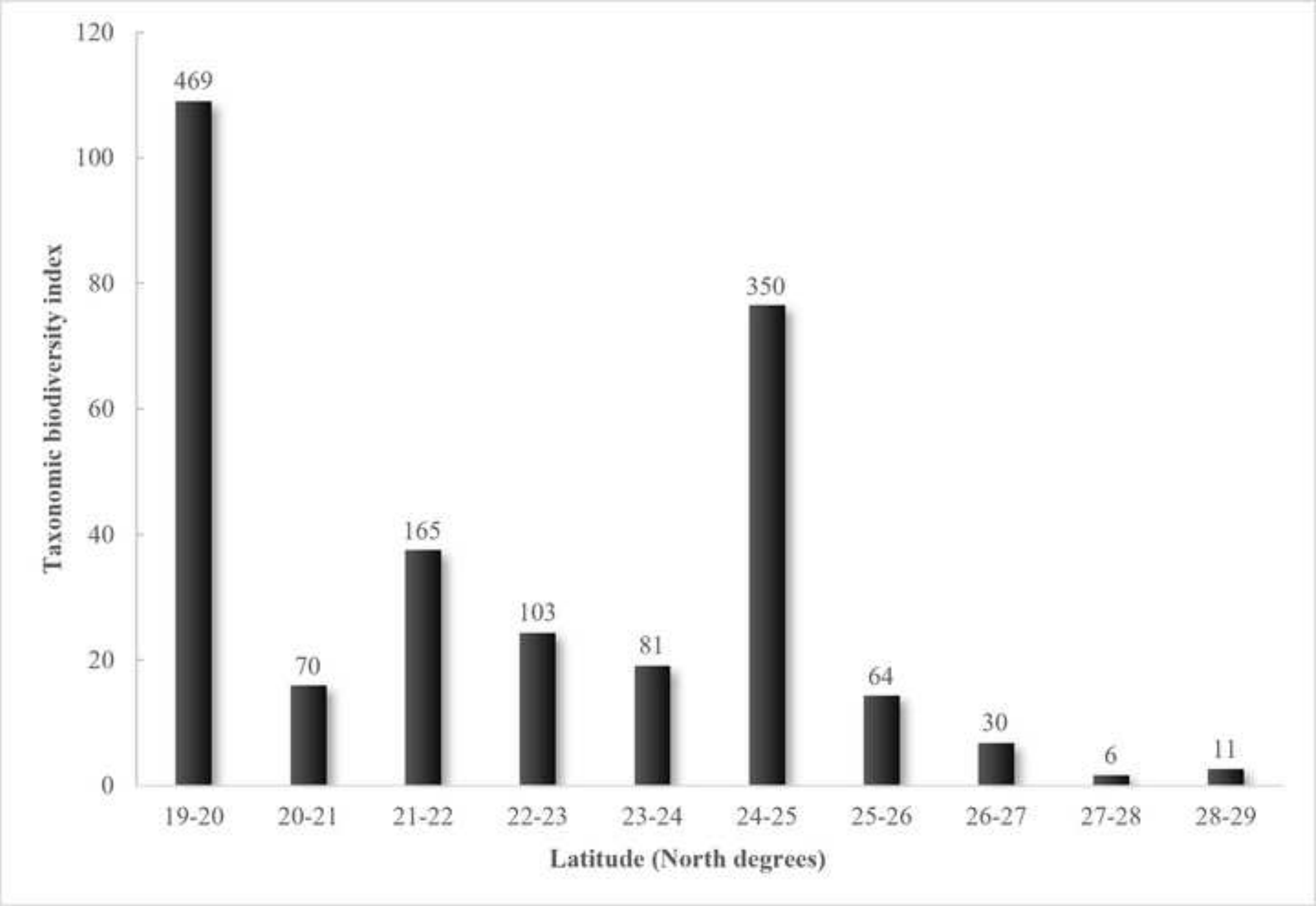

Figure 10

Click here to access/download;Figure;Figure 10.tif

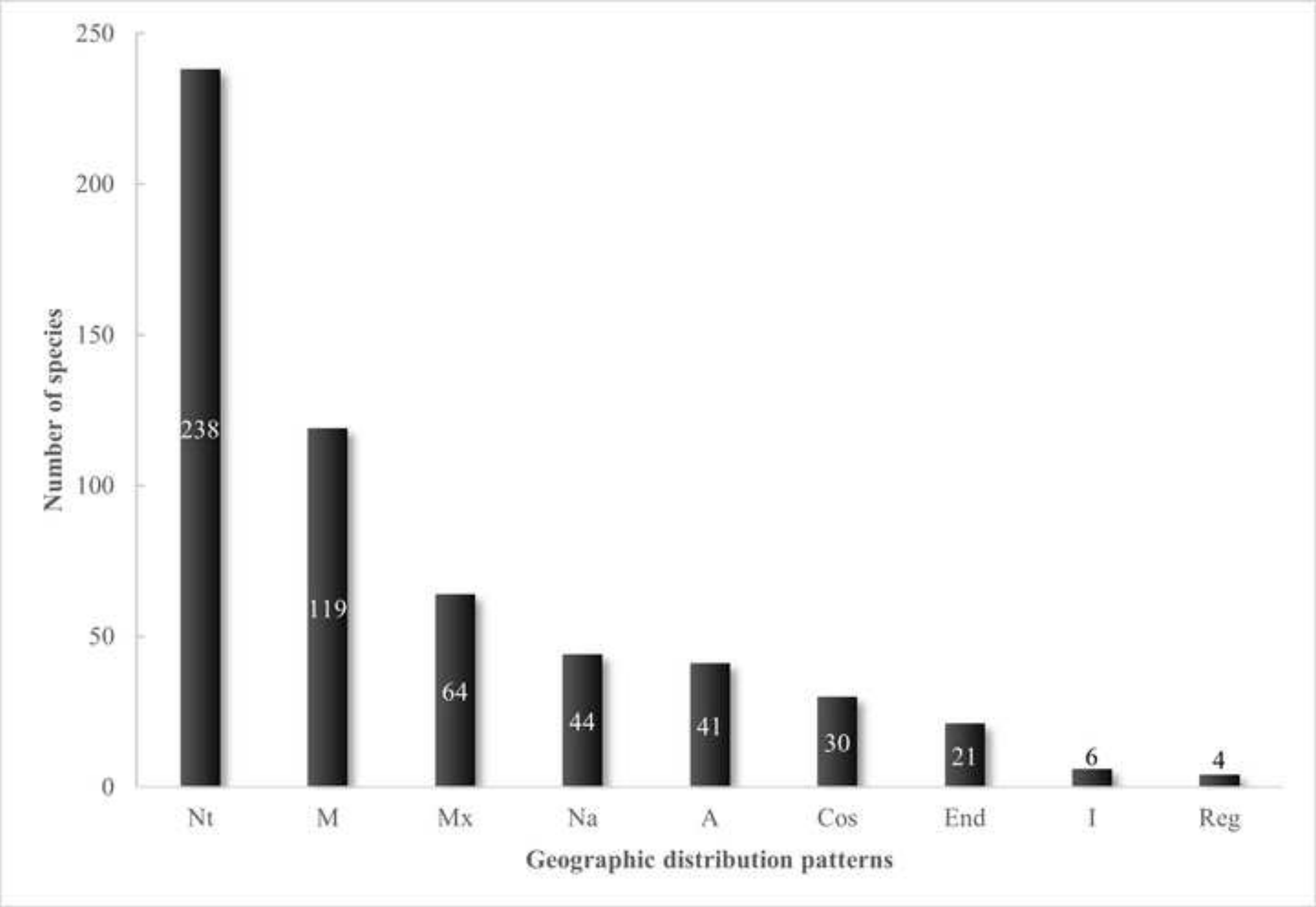

Figure 1

Click here to access/download;Figure;Figure 1 new.tif

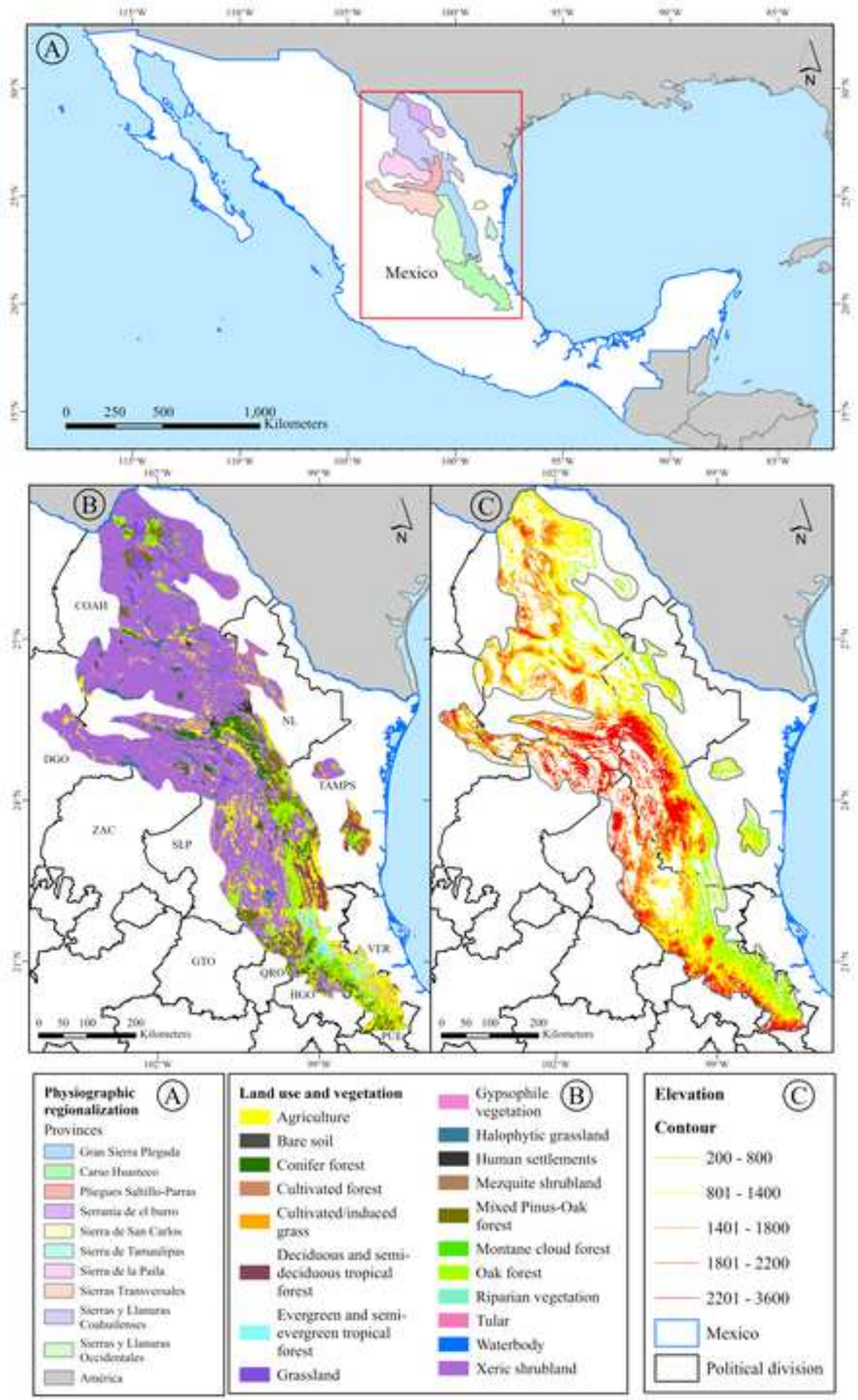

Click here to access/download
Supporting Information
Appendix.xlsx

Richness and biogeography of Pteridoflora in montane forests of eastern Mexico

J. Daniel Tejero-Díez[1], Perla V. Rodríguez-Sánchez[1], Raúl Contreras-Medina[2], Julio Cesar Ramírez-Martínez[3], Jonathan D. Amith[4], Leccinum J. García-Morales[5], Celia Sanginés-Franco[3], and Isolda Luna-Vega[3]*

[1] Laboratorio de Botánica Estructural, Carrera de Biología, Facultad de Estudios Superiores Iztacala, Universidad Nacional Autónoma de México (UNAM), Tlalnepantla, Estado de México, México

[2] Laboratorio de Biodiversidad, Facultad de Ciencias, Universidad Autónoma Benito Juárez de Oaxaca (UABJO), Oaxaca de Juárez, Oaxaca, México

[3] Laboratorio de Biogeografía y Sistemática, Departamento de Biología Evolutiva, Facultad de Ciencias, Universidad Nacional Autónoma de México (UNAM), Coyoacán, México City, México

[4] Department of Botany, National Museum of Natural History, Smithsonian Institution, Washington DC, United States of America

[5] Departamento de Posgrado e Investigación, Instituto Tecnológico de Ciudad Victoria (ITCV), Victoria, Tamaulipas, México

*Corresponding author

E-mail: luna.isolda@gmail.com (ILV)

Abstract

This study compiles and updates the checklist of ferns and lycophytes from the Sierra Madre Oriental (SMOR), Mexico. We analyzed the distribution and richness of these groups of seedless vascular plants to ensure the accuracy and reliability of the data. We reviewed information on these taxa from regional floristic studies, field explorations, and herbarium specimens. Our updated list includes 567 species, of which 511 are ferns and 56 lycophytes, organized into 37 families and 137 genera. The five richest families are Pteridaceae (131), Polypodiaceae (76), Dryopteridaceae (68), Aspleniaceae (45), and Selaginellaceae (41). The five most diverse genera are *Asplenium* (42), *Elaphoglossum* (30), *Pleopeltis* (24), *Myriopteris* (22), and *Selaginella* (21). The Mexican states with the greatest species richness within the SMOR are Puebla (423 species), Hidalgo (322), Querétaro (227), and Tamaulipas (181). The five grid cells with the highest numbers, each containing more than 190 species, are situated in the southern part of the SMOR (Puebla and Veracruz). The physiographic provinces with the greatest diversity are Carso Huasteco and the Gran Sierra Plegada, with 523 and 175 species, respectively. Latitudinal analysis indicated low richness at the northern extreme (28 °- 29 ° N, 11 species) and high richness at the southern extreme (19 °- 20 ° N, 469 species), with a notable peak at 24 °- 25 ° N (350 species). The highest diversity within elevation intervals occurs at 1,000-1,500 m (455 species). The SMOR contains 22 species of the pteridoflora classified under various risk categories according to the Mexican Official Norm NOM-059-SEMARNAT-2010, including seven listed on the IUCN Red List and ten protected under CITES. The taxonomic diversity index shows that the SMOR is among Mexico's most species-rich mountain ranges. The species richness documented here in the SMOR accounts for 50-54%

of the pteridoflora diversity recorded in Mexico, increasing to 59.3% based on recent floristic studies conducted in the region.

Keywords: conservation, diversity, ferns, floristics, lycophytes, montane environments.

Introduction

In the montane areas of tropical regions, the lycophytes and ferns are two lineages of vascular plants that are highly diverse worldwide [1, 2, 3]. These two seedless plant groups are known as pteridobionts because they share the same double-phase life cycle [4, 5, 6]. This taxonomic success and abundance in environments with high atmospheric humidity is due to both the sexual phase (zygote formation) and the sporophyte phase (low stomatal reactivity) being sensitive to water deficit [7, 8, 9]. Make them ideal indicators of the conservation quality of tropical montane cloud forests [10, 11]. Therefore, biological studies in biogeography, ecology, or taxonomy are crucial to our understanding of various aspects of ecosystem management.

In the Neotropics, Mexico is one of the countries with a highly diverse pteridoflora [1] according to Hassler [12] with 1,057 species, including 99 endemics. If hybrids and infraspecies are included, the number of taxa increases to 1,123 (sensu Tejero-Díez database). These taxa account for almost 5% of the country's total vascular flora, which includes 23,314 species (sensu [13]), of which 20% are endemic [14]. The Mexican pteridoflora has been extensively studied from a taxonomic perspective [15], and recent nomenclatural updates have been incorporated into the Mexican checklists of ferns and lycophytes [12]. Nonetheless, investigating certain regions —where information remains scattered or incomplete— remains necessary. Ultimately, understanding the historical

processes behind distribution and the ecological and environmental relationships within species is crucial.

Knowledge about the spatial patterns of richness and endemism in Mexican pteridoflora is limited, especially in highly biodiverse areas. The Sierra Madre Oriental (SMOR) is the second largest mountain range and is regarded as a highly biodiverse Mexican biogeographic province [16, 17, 18], where ferns and lycophytes contribute significantly to this biodiversity. The SMOR boasts a higher floristic richness than any other mountainous region in Mexico, with 8,472 species [18]. This richness is attributed to several factors, such as its geographic location on both sides of the Cancer tropic, complex topography, diverse climate types, the presence of nearly all Mexican vegetation types, and a broad elevational gradient ranging from 100 to over 3,600 meters [17].

Previous floristic checklists and biogeographic research related to ferns and lycophytes have been conducted in the main mountain ranges of Mexico, such as the Sierra Madre del Sur [6], the Sierra Madre Oriental [19, 20], the Trans-Mexican Volcanic Belt [21], and the Sierra Madre Occidental [14], as well as the Chiapas Highlands [22]. Recently, floristic checklists of the SMOR covering all vascular plants have been published [18, 19], but our review of the pteridoflora has identified issues like synonymies, incorrect geographic coordinates, mistaken taxonomic identifications or confusion of particular species, as well as species not recorded in the cited studies but present in the SMOR. Additionally, the study by [20], conducted in the SMOR, includes only a subset of leptosporangiate ferns, the most diverse group of ferns, with an estimated 10,323 species worldwide [23].

The aims of this study were: (1) to carry out an updated and revised floristic inventory of the pteridoflora of the SMOR and (2) to analyze the distributional patterns of ferns and lycophytes using three different area units (states, physiographic provinces and grid cells of 30 × 30 minutes latitude/longitude) and to identify elevational and latitudinal richness patterns, (3) to compare the species richness of the SMOR with other Mexican mountain chains and among the physiographic provinces of the SMOR using a taxonomic diversity index, and (4) to revise the categories of threatened taxa of ferns and lycophytes based on the IUCN Red Lists, the Convention on International Trade in Endangered Species of Wild Fauna and Flora (CITES), and the federal regulatory framework established by the Mexican government (NOM-059-SEMARNAT-2010).

Materials and Methods

Study area

The Sierra Madre Oriental (SMOR) is in northeastern Mexico (Fig. 1A) and is the country's second most important and extensive mountainous province system [16, 24]. This montane region is regarded as part of the Mesoamerican hotspot proposed for ferns and lycophytes by [3]. Geologically, the SMOR resulted from the deformation of Cenozoic and Mesozoic marine sedimentary rocks that were uplifted, shortened, and transported north-eastward, creating a fold-and-thrust belt during the Laramide orogeny [25].

The SMOR extends 800 km in length, with an amplitude of 80-100 km [25]. Although various biotic and abiotic criteria have led to different proposed boundaries for the SMOR [16], we adopted the regionalisation framework of [26], which is based on

physiographic regionalisation (Fig. 1A). This regionalisation scheme of the SMOR has also been applied in studies examining the distribution of Cactaceae [27], leptosporangiate ferns [20], and vascular plants [18]. Within this mountain system, 10 physiographic subprovinces are recognized: Sierras Transversales, Pliegues de Saltillo-Parras, Sierras y Llanuras Occidentales, Gran Sierra Plegada, Sierra de San Carlos, Sierra de Tamaulipas, Carso Huasteco, Serranía de El Burro, Sierra de la Paila, and Sierras y Llanuras Coahuilenses. The SMOR constitutes a biogeographic province of Mexico [16, 24], and together with the Trans-Mexican Volcanic Belt, the Sierra Madre del Sur, and the Sierra Madre Occidental, forms part of the Mexican Transition Zone [24]. The SMOR covers parts of the Mexican states of Coahuila, Durango, Guanajuato, Hidalgo, Nuevo León, Puebla, Querétaro, San Luis Potosí, Tamaulipas, Veracruz, and Zacatecas (Figs. 1B, C). Its latitudinal extent spans from the 20th to the 30th parallel, bounded to the north by the Grande (Bravo) River and to the south by the Trans-Mexican Volcanic Belt [16]. Its location places it in contact with key atmospheric systems, including the subtropical high-pressure belt, trade winds, winter cold fronts, and summer tropical storms linked to hurricanes. This, combined with a latitudinal range of ten degrees, and elevational variations from around 100 m in the lowlands of Tamaulipas and San Luis Potosí [17] to 3,713 m above sea level at Cerro El Potosí in Nuevo León (with an average of 1,000 to 1,500 m, Fig. 1C), and the mountain chain running parallel to the Gulf of Mexico opposite the entry of humid winds, produces a significant orographic shadow effect. This results in a complex climate mosaic, with sites experiencing high rainfall, others experiencing dry seasons, and warm- and temperate-climate conditions, and gradients between these conditions [28]. Furthermore, there are isothermal regimes and extremes, with temperature fluctuations of up to 14°C between the hottest and coldest months. Generally, the climate becomes drier and more extreme towards

the north of the SMOR, with its western slope facing the Mexican Plateau. Nearly all recognized Mexican climatic types are present within this area. Vegetation in the SMOR includes xeric shrubland, conifer forests, oak forests, montane cloud forests, tropical forests, and mixed forests [16, 17] (Fig. 1B).

Figure 1. Maps of northeastern Mexico covering the Sierra Madre Oriental (SMOR). A. Physiographic regionalization. B. Land use and vegetation types. C. Elevation. State abbreviations: Coahuila = COAH, Durango = DGO, Nuevo León = NL, Zacatecas = ZAC, Tamaulipas = TAMPS, Querétaro = QRO, Veracruz = VER, Guanajuato = GTO, Puebla = PUE, San Luis Potosí = SLP, Hidalgo = HGO. Map created by the authors using ArcGIS Pro. All vector layers (Physiography, Land Use and Vegetation Series VII, Hypsometry/Topography, and Political Division) were sourced from INEGI (https://www.inegi.org.mx/), used under the Terms of Free Use of Information which permit commercial utilization.

Formatted: Font color: Text 1

Formatted: Left

Data sources

We obtained distributional data for ferns and lycophytes from three main sources: 1) the review of floristic studies conducted in the SMOR [4, 18, 19, 29, 30, 31, 32, 33, 34, 35, 36, 37, 38, 39, 40, 41, 42, 43, 44, 45, 46, 47, 48, 49, 50, 51, 52, 53, 54, 55, 56, 57, 58, 59, 60, 61], 2) the revision of herbarium vouchers deposited in herbaria such as ARIZ, ASU, BH, BM, BRIT, BRY, CIB, CNS, DUKE, ENCB, F, FCME, GBH, HGOM, HPC, HUAP, HZAC, IEB, IND, ITCV, LAM, LL, MEXU, MICH, MIN, MMNS, MO, MSC, MU, NCU, NY, P, PH, QMEX, SJNM, TAES, TEX, UAMIZ, US, UAT, UC, UCR-BPS, USF, UT,

UTEP, VDB, WIS, WLM, WTU, XAL, and YU (acronyms following [62], 3) field surveys in some Mexican states, such as Hidalgo, Querétaro, Puebla, and Tamaulipas, to collect botanical specimens. Voucher specimens collected during the fieldwork were deposited at the National Herbarium of Mexico (MEXU), herbarium of the Benemérita Universidad Autónoma de Puebla (HUAP), the herbarium of the Instituto Tecnológico de Ciudad Victoria (ITCV), and the United States National Herbarium (US). We revised all taxonomic names and excluded synonyms and misidentifications, as described in [12]. We reviewed the known distribution as described in [15]. We verified the accurate identification of endemic species and those considered rare (with 1 to 3 specimens in the total number of herbaria reviewed or field collections). The accurate identification of names that did not match expert reports in the literature was also reviewed. We compiled a database from all these information sources, including 4,479 georeferenced records of lycophytes and ferns in the SMOR. Using this information, we created geographic distribution maps of the species with ArcGIS Pro [63].

Since the publication of 'The Pteridophytes of Mexico' by [15], new combinations, genera, species, and records of ferns and lycophytes have been published and reported. For these reasons, and in accordance with the International Code of Nomenclature for algae, fungi, and plants (Shenzhen Code) [64], we verified the correct names of each species. The nomenclature and author citations were standardized following the [12] database (except in *Selaginella* sensu [65] and updated to align with the latest integrative and circumscription taxa systematics based on the proposal of the Pteridophyte Phylogeny Group [23].In our database, as is common with herbarium specimens, duplicate samples are found across different botanical collections. In our study, we removed duplicate records from the

biogeographic analysis to ensure unique locations. Additionally, we identified and corrected records with swapped latitude and longitude values. We also excluded specimens with vague localities and inconsistencies. For herbarium specimens lacking geographic coordinates, we used topographic maps at a 1:50,000 scale from the Instituto Nacional de Estadística y Geografía (INEGI) and the previously published locality list for the Sierra Madre Oriental [66] to obtain the corresponding coordinates. Ultimately, we aimed to include only georeferenced and verified data in our study.

To visualize the spatial distribution of collection records and identify potential sampling biases across the SMOR, we created a heatmap from all georeferenced occurrences included in the database. The heatmap was generated in ArcGIS Pro [63] using the Point Density tool, which measures the spatial intensity of records. This method allowed us to highlight areas with high densities of fern and lycophyte collections, enabling the detection of well-sampled regions and zones with limited survey effort. The resulting layer served as a valuable tool for evaluating the dataset's spatial completeness and providing context for subsequent analyses of species richness and distribution patterns.

Biogeographic and diversity analyses

Mexican states were included in the SMOR, physiographic provinces were used to perform the biodiversity analyses, and 30 × 30 minute grid cells were used as units of analysis in ArcGIS Pro [63]. In the grid analysis, we recorded 86 grid cells containing at least one fern or lycophyte species from this framework. The spatial size of grid cells provides a reliable spatial resolution for the distribution compared with other studies using

plants in the SMOR [18, 20, 27] regarding biodiversity data and biological conservation. We selected this scale size because similar scales have been tested in previous studies on biogeography and the diversity of different biological groups of the Mexican biota [67, 68], including ferns and lycophytes [69].

The total expected richness of ferns and lycophytes in the SMOR was estimated using a nonparametric abundance model (Chao1) in EstimateS [70] to evaluate its performance and determine whether it fits the data.

In the grid cell analysis, we used three standard biodiversity metrics (species richness, weighted endemism, and corrected weighted endemism) to measure the biodiversity patterns previously used for different groups of the Mexican pteridoflora [20, 69, 71, 72]. These metrics are more than just theoretical concepts; they are practical tools that have been effectively used in the study of the Mexican pteridoflora [73]. We measured species richness as the total number of species present within each grid cell. Each fern or lycophyte species was counted as present in a grid cell, regardless of whether it was recorded once or multiple times in that cell [27, 74]. The weighted endemism (WE) involves several steps [73]. First, we divided each grid cell occurrence by the number of grid cells in which one species occurs. For example, a fern species limited to a single grid cell was scored as ‘1’ for that grid cell and ‘0’ for all remaining grid cells, whereas a fern species found in five grid cells was scored as ‘0.2’ for each of the five grid cells and ‘0’ for all other grid cells; then we obtained the sum of all score species values for each grid cell. We calculated the corrected weighted endemism (CWE) by dividing the WE value by the number of species in a grid cell [73, 74]. The WE index was considered a biodiversity measure sensitive to species richness, suggesting that richness patterns partially mirror the

WE value [27, 74]. In contrast, the CWE index emphasizes grid cells that contain a proportion of species with limited ranges, which are not necessarily linked to species-rich grid cells [27, 74].

The biodiversity metrics mentioned above were calculated using the software Biodiverse, version 3.0 [75], and mapped in ArcGIS Pro [63]. Grid cells containing only one species were excluded from the CWE analysis because they lack overlapping distributions, which is a key criterion for identifying areas of endemism [76, 77]. Grid cells with the highest scores in the WE index were considered centers of richness, while those with the highest values in the CWE index were regarded as centers of endemism [73, 74].

The geographic distribution of ferns and lycophytes inhabiting the SMOR was established based on the study of [15] as a general framework. We defined nine categories related to the geographic distribution of each species as follows: 1) Cosmopolitan, taxa distributed worldwide; 2) American, taxa distributed across the American continent; 3) Nearctic, taxa found in Mexico (and sometimes in Mesoamerica) and North America; 4) Neotropical, taxa found in Mexico, Central America, the Antilles, and South America; 5) Mesoamerican, taxa distributed in Mexico and Central America; 6) Endemic to the Mexican territory; 7) Endemic to the SMOR, taxa with a known distribution limited to the boundaries of the SMOR; 8) Regional endemic, in adjacent areas to the SMOR; and 9) Introduced taxa.

We determined the frequency of each fern and lycophyte species following the method recommended by [78], based on the number of records for each species and assigned them to their respective frequency classes. This reflects that some species will thrive at the expense of others, leading to differences in species frequency, with a few

species having many individuals and others being rare. We divided the species into five classes of record frequency according to the following ranges: A, 1-11 records; B, 12-23; C, 24-35; D, 36-47; and E, 48-59.

Typically, the species richness of a specific flora is expressed as the number of species or as a biodiversity index that accounts for the number of species per area on a logarithmic scale. To compare the richness of lycophytes and ferns among the main Mexican mountain ranges and the physiographic provinces of the SMOR, we used the taxonomic diversity index (IB), which accounts for the number of species per area on a logarithmic scale, following the formula IB = S/Ln A, where S is the number of recorded species and A is the size of the area [79]. Therefore, using biodiversity indices is crucial for comparing sampled areas of different sizes. Using this formula, we assessed species richness across geographic regions, specifically the main Mexican mountain ranges and the physiographic provinces of the SMOR.

Finally, we assessed the species richness of lycophytes and ferns across different latitudes and elevation gradients. The SMOR covers a wide elevation range from 100 m to 3,713 m above sea level. Based on this, we divided the elevation into eight intervals of 500 m each to illustrate species distribution along this gradient in a graph. These intervals have been used in previous studies on ferns and lycophytes in other Neotropical areas [14, 80, 81, 82], so we applied them to our data for comparison.

We divided the SMOR into 1° latitudinal belts to assess the relationship between species richness and latitude. Additionally, species richness was calculated for all grid cells within each 1° latitude belt from 19°-20° N to 28°-29° N. The species richness values for

each latitudinal belt were determined using the taxonomic diversity index (IB) for that belt [83, 84]. We presented the results of this analysis on a graph showing the taxonomic diversity index values against latitude.

Conservation status

We revised the threatened taxa categories for ferns and lycophytes inhabiting the SMOR in accordance with the Mexican federal regulatory framework [85], the IUCN Red Lists [86], and the Convention on International Trade in Endangered Species of Wild Fauna and Flora (CITES) [87]. Regarding the Mexican risk categories, we reviewed the Mexican Official Norm NOM-059-SEMARNAT-2010 [85] (hereafter, NOM-059) to determine how many ferns or lycophytes are listed and to confirm the assigned risk category for each species. The NOM-059 is the official document issued by the Mexican government, and lists threatened species of plants, fungi, and animals [85]. This document outlines the federal regulatory framework, including the specifications for inclusion and analysis to assess the risk of threatened species. Similarly, the risk categories were assessed according to the IUCN Red List of Threatened Species [86] and the CITES [87] lists. Comparing the IUCN Red List with the Mexican Official Norm NOM-059, some categories can be considered equivalent, such as the threatened category in the Mexican Official Norm to the vulnerable category of the IUCN. In contrast, the special protection category of the NOM-059 includes some minor categories from the IUCN [88].

Results

Based on examined herbarium specimens, web databases, specialized literature, and specimens collected during field expeditions, we compiled a database of 4,479 georeferenced accession records (Fig. 2A). These records correspond to 567 species from the SMOR, belonging to 137 genera and 37 families. From this checklist, 511 species (34 families, 123 genera) are from Polypodiidae, while 56 (3 families, 13 genera) belong to Lycopodiidae. We identified 211 species of ferns and lycophytes that were not previously documented in the floristic studies conducted by [19] and [68] in the SMOR. Of the total names referenced in the studies, 56 were rejected or excluded because they were synonyms or did not represent species found in the study area or the country.

The heatmap (Fig. 2B) visually shows the record density and reveals spatial patterns in sampling effort. It displays the distribution of collection records, with yellow and red colours indicating areas with a high number of records, and blue indicating areas with fewer records, interpreted as well-sampled and under-sampled regions, respectively. The highest concentration of records is in the central part of the country.

Figure 2. A. Map of the SMOR showing georeferenced records of ferns and lycophytes. B. Heatmap showing the sampling effort of ferns and lycophytes. Map created by the authors using ArcGIS Pro. Physiographic regionalization (SMOR) and Political Division layers were sourced from INEGI (https://www.inegi.org.mx/), used under the Terms of Free Use of Information which permit commercial utilization.

We found that the most diverse families (20 or more species) in the SMOR are Pteridaceae (131 species; 23.1 %), Polypodiaceae (76; 13.4 %), Dryopteridaceae (68; 12.0 %), Aspleniaceae (45; 7.9 %), Selaginellaceae (41; 7.2 %), Thelypteridaceae (34; 6.0 %), and Hymenophyllaceae (28; 4.9 %) (Table 1). Nine families are represented by a single species each: Culcitaceae, Didymochlaenaceae, Lindsaeaceae, Lonchitidaceae, Lygodiaceae, Onocleaceae, Plagiogyraceae, Saccolomataceae, and Schizaeaceae.

Table 1. Families of ferns and lycophytes represented in the SMOR. We recorded the number of species and genera in each family and endemics.

**Families**	**Species (%)**	**Genera**	**Endemic species**	**Most diverse genera**
Anemiaceae	12 (2.1)	1	*Anemia* 1	
Aspleniaceae	45 (7.9)	2	*Asplenium* 6	*Asplenium* (42)
Athyriaceae	19 (3.4)	3		
Blechnaceae	12 (2.1)	5	*Woodwardia* 1	
Culcitaceae	1 (0.2)	1		
Cyatheaceae	10 (1.8)	3		
Cystopteridaceae	2 (0.4)	1		
Dennstaedtiaceae	13 (2.3)	6		
Dicksoniaceae	2 (0.4)	2		
Didymochlaenaceae	1 (0.2)	1		
Dryopteridaceae	68 (12.0)	12	*Ctenitis* 2, *Dryopteris* 1, *Elaphoglossum* 10, *Megalastrum* 2, *Phanerophlebia* 1	*Elaphoglossum* (30)
Equisetaceae	4 (0.7)	1		
Gleicheniaceae	7 (1.2)	4		
Hymenophyllaceae	28 (4.9)	**7**	*Didymoglossum* 1	

Families	Species (%)	Genera	Endemic species	Most diverse genera
Isoetaceae	2 (0.4)	1	*Isoetes* 2	
Lindsaeaceae	1 (0.2)	1		
Lomariopsidaceae	2 (0.4)	1	*Lomariopsis* 1	
Lonchitidaceae	1 (0.2)	1		
Lycopodiaceae	13 (2.3)	6	*Huperzia* 1	
Lygodiaceae	1 (0.2)	1		
Marattiaceae	4 (0.7)	2		
Marsileaceae	3 (0.5)	1		
Nephrolepidaceae	6 (1.1)	1		
Onocleaceae	1 (0.2)	1		
Ophioglossaceae	11 (1.9)	5		
Osmundaceae	2 (0.4)	2		
Plagiogyriaceae	1 (0.2)	1		
Polypodiaceae	76 (13.4)	18	*Campyloneurum* 1, *Pecluma* 1, *Pleopeltis* 10, *Polypodium* 3	*Pleopeltis* (24)
Psilotaceae	3 (0.5)	1		
Pteridaceae	131 (23.1)	26	*Adiantum* 1, *Argyrochosma* 2, *Aspidotis* 1, *Astrolepis* 1, *Bommeria* 2, *Gaga* 5, *Myriopteris* 4, *Notholaena* 6, Pellaea 2, Ynesmexia 1	*Myriopteris* (22)
Saccolomataceae	1 (0.2)	1		
Salviniaceae	2 (0.4)	1		
Schizaeaceae	1 (0.2)	1		
Selaginellaceae	41 (7.2)	6	*Bryodesma* 4, *Lepidoselaginella* 2, *Pulviniella* 1, *Selaginella* 7	*Selaginella* (21)
Tectariaceae	3 (0.5)	1		
Thelypteridaceae	34 (6.0)	8	*Amauropelta* 1, *Goniopteris* 1, *Pelazoneuron* 2	

Families	Species (%)	Genera	Endemic species	Most diverse genera
Woodsiaceae	3 (0.5)	1	*Physematium* 1	
**TOTAL 37**	**567** (100.0)	**137**	**89**	**139**

Table 2. Species with more records and recorded in more SMOR grid cells.

Species	Number of records	Grid cells
Pleopeltis polylepis (Roemer ex Kunze) T. Moore	57	25
Pleopeltis plebeia (Schltdl. & Cham.) A. R. Sm. & Tejero	56	20
Polypodium plesiosorum Kunze	53	27
Phlebodium pseudoaureum (Cav.) Lellinger	52	17
Tectaria heracleifolia (Willd.) Underw.	47	20
Campyloneurum angustifolium (Sw.) Fée	41	15
Adiantum capillus-veneris L.	40	26
Pelazoneuron ovatum (R.P. St. John) A.R.Sm. & S.E. Fawc.	40	21
Llavea cordifolia Lag.	36	19
Pleopeltis crassinervata (Fée) T. Moore	36	10
Adiantum tenerum Sw.	35	13
Asplenium monanthes L.	35	18
Astrolepis sinuata (Lag. ex Sw.) D.M. Benham & Windham	35	26
Blechnum appendiculatum Willd.	34	15
Adiantum poiretii Wikstr.	33	15
Pleopeltis acicularis (Weath.) A.R. Sm. & T. Krömer	33	11
Pteridium aquilinum (L.) Kuhn	33	13
Anemia mexicana Klotzsch	31	20
Notholaena candida (M. Martens & Galeotti) Hook.	31	20
Pteris cretica L.	31	19
Astrolepis integerrima (Hook.) D.M. Benham & Windham	30	23

Formatted: Spanish (Mexico)

**Species**	**Number of records**	**Grid cells**
Pleopeltis polypodioides (L.) E.G. Andrews & Windham	30	20
Anemia adiantifolia (L.) Sw.	29	19
Pleopeltis guttata (Maxon) E. G. Andrews & Windham	29	21
Polypodium arcanum Maxon	29	15
Remaining taxa	3,543	2,289
**Total**	**4,479**	**2,757**

State diversity in the SMOR

Our analysis indicates that Puebla has the highest number of fern and lycophyte species, with 423 species (31.9 species/km^2), followed by Hidalgo and Querétaro, which have 321 (23.3 species/km^2) and 227 (17.6 species/km^2), respectively. Tamaulipas and Veracruz have 181 (9.8 species/km^2) and 117 (8.3 species/km^2), respectively, while the other states of the SMOR have fewer than 100 species each. Endemic ferns or lycophytes confined to a single Mexican state were recorded in Puebla and Tamaulipas, with three taxa, and in Nuevo León and San Luis Potosí, with two species.

From the total identified pteridoflora of the SMOR area as defined by [26], we identified an essential subset of 123 species representing new records for the SMOR. A subset of 40 species has an updated distributional range, previously recorded in the SMOR, and is now documented in other states not previously reported in the literature (Fig. 3).

Figure 3. New pteridophyte records for the SMOR (123 species) and species with an extended distribution in other states from the SMOR (40 spp.).

Eleven of the new records belong to *Elaphoglossum*, seven to *Selaginella*, and six are filmy ferns of the genus *Hymenophyllum*. A total of 41 genera are represented by a single species within the new records in the SMOR. The others have between two and five species. The genera with the most species showing range extension into the SMOR are *Notholaena* and *Amauropelta*, with five and four taxa, respectively. Overall, 16 genera have one species extending its range in the SMOR, while the rest are represented by two or three species.

Common vs. rare species

As is common in biogeographic studies based on herbarium data, few fern species are represented by a disproportionate number of specimens, while many are represented by only a few (Fig. 4). In our study, only four species have more than 50 records (*Phlebodium pseudoaureum, Pleopeltis polylepis, Pleopeltis plebeia*, and *Polypodium plesiosorum*), which contrasts with several other species limited to just one or three localities (252 taxa). These latter data account for approximately 50% of the pteridoflora of this mountain range.

Figure 4. Record frequency for each species represented in the SMOR.

Grid cell species richness and endemism indices

We analyzed 86 grid cells from the SMOR (Fig. 5A), each containing at least one record. Species richness values ranged from 1 to 307 species per grid cell (Fig. 5B). The main center of pteridoflora diversity in the southern part of the SMOR (states of Puebla, Hidalgo,

Querétaro, and Veracruz) consisted of nine neighbouring grid cells with the highest species richness scores (>90), ranging from 96 to 307 species (Fig. 5B). The tenth grid cell with the highest richness score (95) is located in Nuevo León (BB). Among the top-scoring grid cells, BX, CC, CD, and CG have the highest number of species (>200). These high-richness grid cells contrast with other parts of the SMOR, where collections are sparse or absent, as in 13 grid cells with only one fern or lycophyte species and eight grid cells with just two taxa recorded (Fig. 5B).

Figure 5. A. Division of the SMOR into grid cells of 30 minutes by side and latitudinal belts of one geographical degree. B. Species richness of the pteridoflora in the SMOR. Map created by the authors using ArcGIS Pro. Physiographic regionalization (SMOR) and Political Division layers were sourced from INEGI (https://www.inegi.org.mx/), used under the Terms of Free Use of Information which permit commercial utilization.

Polypodium plesiosorum (27), *Adiantum capillus-veneris* and *Astrolepis sinuata* (26 each), *Pleopeltis polylepis* (25), and *Astrolepis integerrima* (23) were found in more grid cells, followed by *Pelazoneuron ovatum* and *Pleopeltis guttata* (21) (Table 2). Conversely, 150 species are only represented in one grid cell. Ferns and lycophytes are mainly distributed in temperate forests, such as oak and pine-oak forests, and in tropical montane cloud forests. However, some species prefer other types of Mexican vegetation that receive less rainfall, such as the arid scrub and tropical deciduous forest. Temperate landscapes mainly influence the key distribution patterns observed in this study.

We undertook an effort-weighted sampling method for our manuscript. In our study using the Chao1 richness estimator, we identified 567 species. According to the estimator, the average estimated richness at this sampling level was about 670 species, with a 95% confidence interval of approximately 632-729 species (Fig. 6). The difference between the lower and upper confidence limits is roughly 100 species, indicating moderate uncertainty around the estimate, as expected in communities with many rare or low-detectability taxa.

Figure 6. Species accumulation curve for ferns and lycophytes of the Sierra Madre Oriental (SMOR) using the Chao1 richness estimator. The observed species richness is shown as a solid line. The Chao1 estimator is represented by three curves: the mean estimated richness and its lower and upper confidence limits (95%).

Comparing the observed richness (567 species) to the Chao1 mean estimate (≈670 species), our data falls short by about 100 species from the estimated total richness. This

~~s~~suggests that our sampling completeness is roughly 85%, which is relatively high, because Chao1 uses the number of very infrequently observed species to infer unseen diversity, tending to produce higher richness estimates when rare species are relatively common in the sample [89, 90]. These curves show that we've captured more than 85% of the expected species at a sampling site, indicating that this type of analysis is feasible [91].

Overall, these results suggest that although the sampling did not fully capture the total estimated richness, it still documented a significant portion of the community. Using Chao1, the accumulation curves for fern and lycophyte species in the SMOR did not reach a defined asymptote, remaining above the observed richness.

Endemism indexes

Figures 7A and 7B display the values of weighted endemism (WE) and corrected weighted endemism (CWE) for ferns and lycophytes of the SMOR. For WE, values ranged from 0.04 to 115.8 across the grid cells analyzed (Fig. 7A). The WE values showed a pattern similar to that of species richness. We found that nearly all grid cells with the highest species richness scores also recorded the highest WE values (>16), except for the grid cell BW.

The corrected weighted endemism values ranged from 0.05 to 0.48 (Fig. 7B), excluding grid cells with only one species, as previously established. The grid cells with the highest CWE values (>0.25) are spread throughout the SMOR, and only one grid cell (CD) with the highest species richness is included.

Figure 7. A. Weighted endemism (WE). B. Corrected weighted endemism (CWE). Map created by the authors using ArcGIS Pro. Political Division layers were sourced from INEGI (https://www.inegi.org.mx/), used under the Terms of Free Use of Information which permit commercial utilization.

Elevation patterns

The pteridoflora in the SMOR is found across the entire elevation range of this mountain range (100 to 3700 m). As is typical in Neotropical mountains, species richness in the SMOR exhibits a hump-shaped elevational pattern, with the highest diversity at mid-elevations (Fig. 8). The greatest number of species occurs between 1000 and 1500 m (455 species), decreasing at both higher and lower elevations.

Figure 8. Elevational distribution of the pteridoflora in the SMOR.

Latitudinal patterns

We believe that the pteridoflora of the SMOR did not strictly follow a latitudinal pattern. Species diversity does not consistently decrease or increase with latitude (Fig. 9). Our analysis of latitude within 1 ° geographic belts (Fig. 5A) revealed low richness at the northern extreme (28°-29° N, 11 species) and high richness at the southern extreme (19°-20° N, 469 species). Another peak in richness occurs at the 24 °- 25 ° N belt (with 350 species). The decline in species richness is more apparent from the 24°-25° N belt to the northernmost point (28°-29° N).

Figure 9. Latitudinal belts of one geographic degree and their values of the taxonomic biodiversity index. The numbers over each column represent the species recorded in each latitudinal belt.

Geographic distribution patterns

Regarding the geographic distribution of ferns and lycophytes inhabiting the SMOR, the Neotropical and Mesoamerican components are the best represented, with 238 and 119 species, respectively (Fig. 10) (see Appendix). They are followed by the endemic component to Mexico, which has 64 species (Fig. 10). Other significant components include the Nearctic and American, represented by 44 and 41 species. The high proportion of Neotropical and Mesoamerican components in the SMOR enhances the significance of this mountain range within the Mexican Transition Zone. Each remaining geographic component has fewer than 30 species (Fig. 10).

We recorded 21 species that are endemic to the SMOR: *Asplenium dianae*, *Asplenium semipinnatum*, *Asplenium ultimum*, *Bryodesma carnerosanum*, *Gaga apiacea*, *Gaga hintoniorum*, *Goniopteris schaffneri*, *Isoetes tamaulipana*, *Myriopteris chipinquensis*, *Myriopteris maxoniana*, *Notholaena brachycaulis*, *Notholaena brevistipes*, *Notholaena jacalensis*, *Notholaena leonina*, *Pellaea notabilis*, *Pellaea ribae*, *Pleopeltis ×gracilis*, *Pleopeltis ×pueblensis*, *Pleopeltis fallacissima*, *Pulviniella gypsophila*, and *Woodwardia martinezii*. At least five other species are closely associated with the SMOR and have geographic distributions almost restricted to this mountain chain (Appendix).

Figure 10. Geographic distribution patterns of the pteridoflora of the SMOR. The abbreviations are: (Cos) Cosmopolitan; (A) American; (Na) Nearctic; (Nt) Neotropical; (M) Mesoamerican; (Mx) Endemic to Mexico; (End) Endemic to the SMOR; (Reg) Regional endemic; (I) Introduced.

Risk categories

There are 20 fern species and two lycophytes listed in the Mexican Official Norm NOM-059 [85]. These species are shown in Table 3. Among them, 12 are classified as special protection category (Pr), 7 as threatened (A), and 3 as at risk of extinction (P). The three species are *Cyathea costaricensis, Cyathea godmanii,* and *Selaginella porphyrospora*. According to the Red Lists of Threatened Species by the IUCN [86], seven fern taxa are categorized as least concern (LC), indicating a low risk of extinction (see Table 3). Only members of Cyatheales are included in Appendix II of CITES [87], comprising eight Cyatheaceae and two Dicksoniaceae (see Table 3).

Table 3. Threatened species of ferns and lycophytes of the SMOR and their risk categories.

**Family**	**Species**	**NOM-059**	**IUCN**	**CITES**
Aspleniaceae	*Asplenium auritum* Sw.	A		
	Asplenium formosum Willd.		LC	
	Asplenium serratum L.	A		

Family	Species	NOM-059	IUCN	CITES
Culcitaceae	*Culcita coniifolia* (Hook.) Maxon	Pr		
Cyatheaceae	*Alsophila firma* (Baker) D. S. Conant	Pr		Appendix II
	Cyathea bicrenata Liebm.	Pr		Appendix II
	Cyathea costaricensis (Mett. ex Kuhn) Domin	P		Appendix II
	Cyathea fulva (M. Martens & Galeotti) Fée	Pr		Appendix II
	Cyathea godmanii (Hook.) Domin	P		
	Cyathea microdonta (Desv.) Domin	Pr		Appendix II
	Cyathea myosuroides (Liebm.) Domin	Pr		Appendix II
	Cyathea schiedeana (C. Presl) Domin	Pr		Appendix II
	Cyathea tuerckheimii R. M. Tryon	Pr		
	Sphaeropteris horrida (Liebm.) R. M. Tryon	Pr		Appendix II
Dicksoniaceae	*Dicksonia navarrensis* Christ	Pr		Appendix II

Family	Species	NOM-059	IUCN	CITES
	Lophosoria quadripinnata (J. F. Gmel.) C. Chr.			Appendix II
Equisetaceae	*Equisetum hyemale* L. var. *affine* (Engelm.) A. A. Eaton		LC	
Lycopodiaceae	*Phlegmariurus dichotomus* (Jacq.) W.H. Wagner	A		
Marattiaceae	*Marattia laxa* Kunze	Pr		
	Marattia weinmanniifolia Liebm.	Pr		
Osmundaceae	*Osmunda spectabilis* Willd.		LC	
Polypodiaceae	*Campyloneurum phyllitidis* (L.) C. Presl	A		
	Serpocaulon triseriale (Sw.) A. R. Sm.	A		
Psilotaceae	*Psilotum ×complanatum* Sw.	A		
	Psilotum flaccidum Wall.	A		
Pteridaceae	*Adiantum capillus-veneris* L.		LC	
	Pteris vittata L.		LC	
Selaginellaceae	*Selaginella porphyrospora* A. Braun	P		
Thelypteridaceae	*Cyclosorus interruptus* (Willd.) H. Itô		LC	

Formatted: Danish

Family	Species	NOM-059	IUCN	CITES
	Goniopteris tetragona (Sw.) C. Presl		LC	

Abbreviations. Risk categories of the NOM-059: (A) Threatened; (Pr) Special protection; (P) Endangered. IUCN: (LC) Least concern category.

Discussion

Our study increased the number of ferns and lycophytes recorded in the SMOR compared to earlier floristic surveys conducted in this Mexican mountain range [18, 19]. We documented 567 species, including 511 ferns and 56 lycophytes, thereby expanding our understanding of these plant groups and the Mexican pteridoflora. Our findings reflect an increase of 211 species over the 356 species in the SMOR, as documented by [12, 19], which reported 1,055 species in Mexico. Tejero's personal database (2025) includes 1,124 taxa, encompassing subspecies, varieties, and hybrids. Given this diversity, the new total of species recorded here in the SMOR represents approximately 50-54% of the Mexican pteridoflora. This study also documents at least 56 names and/or species that were excluded following taxonomic refinement and review.

Our study, mainly based on specimen revisions from herbaria and fieldwork, uncovers new records and range extensions for over 100 species of pteridoflora in the SMOR. Our revision identifies 123 species in 62 genera as new for the area and 40 species in 25 genera showing range extensions within this mountain chain. *Elaphoglossum* and *Hymenophyllum* are the fern genera with the most recent records in the SMOR; a similar

situation occurs in an inventory of the pteridoflora in Veracruz [43], where *Elaphoglossum* and filmy ferns were also reported with the highest number of new records among ferns. Our findings confirm that ongoing fieldwork in the mountains of the Mexican Transition Zone could uncover new records and range extensions of the Mexican pteridoflora [43], even in regions that have already been floristically studied, such as the SMOR (e.g., [18, 19].

The geographic position, its alignment with the oceanic edge, and the size of the SMOR influence the observed patterns of differential richness. A common pattern of species richness along the latitudinal gradient, which shows an increase from the north to the tropics, was disrupted. Despite over 200 years of research, the factors driving geographic patterns of species richness remain largely misunderstood [92]. In the case of the SMOr, an intermediate zone of the gradient (24°–25° N) was found to have a relatively high proportion of pteridoflora compared to the southern latitudinal zones (19°–20° N), with 469 species (especially Puebla; 31.9 species/km²) and the northern zone with the lowest (Coahuila 2.7 species/km²).

Most likely, the ecological factor responsible for this intermediate peak in species richness can be attributed to the combination of the broader SMOR band with the effect of orographic shadow, which creates an unparalleled environmental heterogeneity in the mountain range. Additionally, the states on the oceanic slope are more diverse (Querétaro 17.6) than those on the interior (Guanajuato 1.2 species/km²). By analyzing both the latitude and orographic shadow patterns, we can explain the high richness at southern latitudes (20-21°; HGO, PUE, and VER) and the intermediate richness at latitudes 25-26°,

where richness increases from west to east across the mountain (DGO, COAH, and ZAC; and NL, TAMS) (Fig. 9).

As observed in the mountainous regions of southeastern Mexico [6], the SMOR shows a strong Neotropical influence. Many Neotropical and Mesoamerican elements are well-represented in the Sierra Madre del Sur and the Chiapas Highlands in southeastern Mexico. In the SMOR, the flowering plants display a marked Nearctic influence [18], contrasting with the predominant Neotropical affinities of the pteridoflora reported here. Compared to the Sierra Madre Occidental [14], the SMOR showcases the typical association of Neotropical ferns in montane ecosystems due to its high humidity levels, like those found on the Gulf of Mexico slope, and hosts several of the most diverse epiphytic fern clades, including *Asplenium*, *Elaphoglossum*, Hymenophyllaceae, and Polypodiaceae [3]. The diversity of pteridoflora in the SMOR and the presence of endemic species support the idea of a tropical Mexican fern hotspot, with the SMOR forming part of one of the main regional hotspots in the Neotropics recognized by [93].

Pteridaceae, Dryopteridaceae, and Polypodiaceae are regarded as some of the most diverse fern families worldwide. Some genera and subfamilies reflect the dominant ecological zones in the SMOR. Pteridaceae is a successful group represented there by 131 species and is classified into three major bioclimatic groups: adiantoids, pteroids, and cheilanthoids. The cheilanthoid ferns (*Argyrochosma, Astrolepis, Gaga, Myriopteris, Notholaena, Pellaea*) are primarily found on the north and western slopes facing the SMOR, which features xerophytic and seasonal temperate vegetation. Conversely, the adiantoid ferns are mainly present in the eastern part of the SMOR, characterized by a

warm, humid climate. Lastly, the remnant pteroid group (*Pteris*, *Vittaria*) is predominantly located in the temperate, humid montane region [94].

The other two fern families with high species richness in the SMOR are Dryopteridaceae and Polypodiaceae, mainly inhabiting the TMCF on oceanic slopes [19, 95]. In the SMOR, Polypodiaceae includes 18 genera and 76 species, exhibiting the greatest variation in form among ferns, including epiphytic species [96]. Some genera, such as *Campyloneurum*, *Pecluma* (both with 10 species), *Polypodium* (11 species), and *Pleopeltis* (24 taxa), are mainly represented by epiphytic forms in the SMOR [96]. Dryopteridaceae are primarily found in temperate and tropical montane habitats [95]; they comprise 12 genera and 68 species, mostly terrestrial, except for *Elaphoglossum*, a diverse genus with 30 species, some terrestrial and some epiphytic [97]. *Elaphoglossum*, *Pleopeltis*, and *Polypodium* occupy the vertical space of trees and contribute to the high diversity of these forests compared to other regions of the warm zone. Among lycophytes, the richest family in the SMOR is Selaginellaceae, represented solely by *Selaginella*, with 41 species [12]. The list of lycophytes presented in this study is based on [65]. Members of Selaginellaceae thrive under a wide range of climates and soil types. Species with homophyllous leaves (*Bryodesma*) and heterophyllous, rosette-forming leaves (*Lepidoselaginella*) are poikilohydric, drought-adapted xerophytes [98, 99]. The heterophyllous species are diverse in tropical forests worldwide, predominantly in secondary environments [100]. Both mesic and tropical-xeric ecological zones are well represented in the SMOR. The same is true for other Mexican montane ranges, such as the Sierra Madre Occidental [14] and the Sierra Madre del Sur [6], which, along with the Trans-Mexican Volcanic Belt and SMOR, form the Mexican Transition Zone sensu [101]. Conversely, in the southern Mexican mountains

with homogeneous mesic environments, such as the Chiapas Highlands, there are only 24 species [22].

In the heatmap, the concentration of collection records is located in the central part of the country, corresponding to the southern part of the SMOR. This map shows that collecting effort is not randomly distributed, as is often the case in biodiversity studies [102], and the disproportion of records in the southern part of the SMOR may be linked to accessible geographic locations such as roadsides and urban areas, as well as proximity to research institutions in central Mexico with active botanical research. This pattern has been previously suggested for biodiversity studies based on herbarium specimens [102, 103, 104]. The higher number of collection records and species of ferns and lycophytes in the southern SMOR can also be explained by the higher rainfall and warmer temperatures in this region [28]. These climatic conditions support the presence and abundance of these vascular plants in the tropical montane cloud forests, which are preferred by many botanists for the study and field collection of Mexican ferns and lycophytes, as in other parts of the country [5, 95].

The grid cells with high species richness scores include families and genera that typically inhabit humid environments [4]. The convergence of certain families, such as Lycopodiaceae, Selaginellaceae, Aspleniaceae, Athyriaceae, Cyatheaceae, Dryopteridaceae, Hymenophyllaceae, Polypodiaceae, Pteridaceae, and Thelypteridaceae in these highly diverse grid cells reflects complex plant assemblages in these areas, due to the presence of different phylogenetic lineages of the Mexican pteridoflora, linked to epiphytic and terrestrial lifestyles [92].

The estimator values used showed similar behavior and remained close to the observed values, indicating that a good sampling of the pteridophyte flora was achieved [90]. Although the number of pteridophyte species reported in this study is much higher than that recorded in previous research (e.g., [19]), the analysis of the species accumulation curves suggests that more species can still be recorded to complete the list of ferns and lycophytes of the SMOR.

The WE values obtained for ferns and lycophytes in the SMOR are very similar to the species richness. Nearly all grid cells with high species richness scores (except BW) also showed high WE index values. This correlation between species richness and WE values has been suggested in previous studies [27, 73, 74]. The CWE index emphasizes species with limited distribution, so only one grid cell (CD) is represented in the WE with the highest values. The grid cells with high CWE values (≥0.25 and containing two or more recorded species) are spread across different zones of the SMOR and are influenced by the geographic distribution of the species present in these grid cells, some of which are endemic to the SMOR, such as *Asplenium dianae, A. semipinnatum, A. ultimum, Notholaena brachycaulis,* and *Pellaea ribae*.

We regard the CWE index as an essential measure of endemism because it is independent of species richness [69, 73, 74], as it includes species endemic to the SMOR and Mexico. We recommend that the grid cell CD, which appears in both WE and CWE indexes and is recognized as having the highest diversity (307 species), should be a priority area for conserving the Mexican pteridoflora. Further research and fieldwork are needed in grid cells with the highest CWE scores to boost the number of records and species in these areas.

Following the "World Ferns" sensu [12], some species are found in only one Mexican state. Our study revealed that many of these species (e.g., *Pecluma liebmannii* and *Notholaena brevistipes*), previously recorded as endemic to just one state, are also found in another Mexican state. These two species, along with *Ctenitis bullata*, *Pecluma liebmannii*, *Pleopeltis ×melanoneuron*, and *Selaginella subrugosa*, are documented in our study for the first time in one to three Mexican states, changing their endemism status from local endemic to regional endemic.

Considering the high diversity of the pteridoflora in the SMOR (567 species), we observed that only a small percentage of these vascular plants are classified as threatened by international and national agencies. Regrettably, only 22 species are listed under some risk category in the NOM-059 [85] by the Mexican government, and seven are in the least concern (LC) category of the IUCN [86]. This low number of threatened species suggests that other fern and lycophyte taxa need to be assessed according to the criteria of the IUCN [86] and the NOM-059 [85], such as regional endemics and those species limited to the tropical montane cloud forests, to determine their potential inclusion in the National and International Red Lists.

We suggest that the 19 endemic species be assessed for inclusion in NOM-059, as they are regionally endemic and have few documented instances. None of these species is listed on the Red List, and their traits exceed those considered for some species already included. Candidate species include taxa from Pteridaceae (genus *Notholaena*, with five species) from semiarid zones with xerophytic vegetation in northern SMOR.

The *Phlegmariurus* species (Lycopodiaceae) are notable and are not necessarily restricted to SMOR. [105] proposed that all Mexican *Phlegmariurus* species be regarded as

threatened or endangered. These species inhabit undisturbed areas of mature pine-oak and humid montane forests, often represented by only a few individuals at each site [106]. The NOM-059 includes only one species (*P. dichotomus*), categorized as threatened. The genus *Phlegmariurus* in Mexico comprises 13 species, seven of which are found in SMOR. These lycophytes are rare or absent in cleared areas because they are epiphytes of mature host canopy trees; they are located in limited localities and collections [106], such as *P. linifolius* (5 records), *P. pringlei* (5 records), *P. pithyoides* (3 records), and *P. orizabae*, documented from only a single specimen collected in Puebla.

The family Cyatheaceae is a threatened component in the SMOR. The mountain regions of the Neotropics host most of the species richness of American tree ferns [71]. These vascular plants are critically endangered, facing threats from deforestation, exploitation, and global climate change [71, 107, 108]. Most are listed in the NOM-059 , but their risk categories need reassessment and updating [71, 109]. Unfortunately, deforestation and land-use change in the SMOR have reduced tropical mountain forest areas due to agriculture and livestock raising [17, 109], threatening the survival of these ferns. The extraction of tree fern stems for handicrafts and as growing medium or substratum for orchids and other plants poses a serious threat to these ferns [107, 108], and some initiatives have been proposed within the SMOR (e.g., [110]). According to Appendix II of CITES, tree ferns are not necessarily at risk of extinction but could become threatened if their trade is not properly regulated. Therefore, the Mexican government must establish trade regulations for Cyatheaceae species to prevent their exploitation and use that threaten their survival.

Conclusions

In the SMOR, [18] recorded 8,472 vascular plant species, suggesting that this mountain range has greater floristic richness than any other mountainous region in Mexico. Conversely, [19] documented 6,981 vascular plants in the SMOR, including 356 species of ferns and lycophytes; [18] identified at least 104 as 'characteristic' species of the SMOR without listing the total number of taxa. Our findings include 211 species of pteridoflora, exceeding those reported by [19], and thus increasing the overall number of vascular plants in the SMOR.

Herbarium data are crucial for understanding plant biogeography; however, sampling biases and coverage gaps are common in biogeographic studies [104]. Our analysis, mainly based on herbarium specimens, is therefore influenced by these biases. Some areas within the SMOR boundaries lack records of ferns or lycophytes, and 13 grid cells contain only one species. These low-diversity areas may be related to unexplored zones and insufficient sampling, as observed in many sectors of Mexico [67, 69] and the Neotropics [1, 111]. Widespread species are more frequently collected than rare or microendemic species. Future collection efforts in poorly sampled areas of the SMOR are essential to enhance the fern and lycophyte inventory, emphasising the importance and ongoing need for fieldwork [1].

This study provides a detailed inventory of ferns and lycophytes from one of the world's most biodiverse regions, recognized as part of a biodiversity hotspot [93]. This mountain range serves as a transition zone between different biogeographic regions (Neotropical and Nearctic), with diverse climatic, edaphic, and geological conditions and variations in latitude and elevation. This results in high environmental heterogeneity,

leading to significant plant diversity [18]. Based on taxonomic dominance, the most prominent environments are the subhumid temperate (*Pleopeltis* zone) in the middle and high mountains, followed by the semiarid (*Notholaena* zone) on the northern and western slopes, the warm humid (*Adiantum* zone) on the eastern slope, and humid temperate (*Hymenophyllum* zone) in the southern part of the SMOR.

Our findings show that the greatest diversity of ferns and lycophytes is found in the southwestern part of the SMOr, corresponding to the northern mountains of Puebla. This is confirmed by climate data: the southern part of the SMOR receives the most rainfall (>1,200 mm, up to 4,000 mm in Cuetzalan, Puebla) and has the warmest temperatures (between 22 and 26 °C) [28]. Both climate conditions support the presence and growth of many fern and lycophyte species.

Another distinct region is the central part of the SMOR in the state of Tamaulipas (grid cells AY, BB, and BC), which includes the El Cielo Biosphere Reserve and the Sierra de San Carlos. The climate in this section of the SMOR, covering Tamaulipas, features temperate conditions (12-18 °C), with rainfall ranging from 600 to 1,200 mm [28], supporting species with Nearctic affinities. Due to the unique biology of lycophytes and ferns, involving two different phases in their life cycle- one sensitive to humidity [4, 5, 6] and their stomata's low reactivity [7, 8, 9], it is a taxocenosis that functions as an indicator of local environmental conditions. Appropriate vegetation cover and moisture enhance the richness and abundance of pteridoflora. As a result, the distribution patterns observed in this study can serve as a foundation for predicting future environmental impacts, whether from deforestation or climate change.

Note: In this study, we excluded *Microsorum grossum* (Langsd. & Fisch.) S.B. Andrews, an exotic species growing as an epiphyte in nearby zones of Gómez Farías, Tamaulipas [111]. At the final stage of this research, *Adiantum wilesianum* Hook. was recorded in three localities, two from San Luis Potosí and one from Hidalgo, which are not represented in our analyses.

Contributor roles

JDTD: conceptualization, data curation, writing – original draft, writing – review & editing. PVRS: conceptualization, data curation, writing – original draft. RCM: formal analysis, investigation, methodology, writing – original draft, writing – review & editing. JCRM: formal analysis, investigation, methodology. JDA: supervision, validation. LJGM: data curation. CSF: formal analysis, methodology; ILV: conceptualization, formal analysis, funding acquisition, investigation, methodology, project administration, resources, supervision, writing – original draft; writing – review & editing.

Acknowledgements

We are grateful to the curators and staff of the herbaria mentioned in the text for their courtesy during our review of specimens. ~~Project DGAPA-PAPIIT IN219424 funded the project.~~ The HUAP, through Amparo Cerón, helped develop a database from northern Puebla. The IEB, through Brenda Bedolla, helped develop the database from northeastern Querétaro related to the SMOR. CONABIO, through Susana Ocegueda, helped develop the

database from Veracruz. Jonathan D. Amith, co-author, contributed to this research effort as director of a long-term floristic and ethnobotanical project in the Sierra Nororiental de Puebla, during which > 600 ferns and allies were collected, principally in the municipalities of Cuetzalan del Progreso (182), Zacapoaxtla (157) Zautla (72), Zoquiapan (58), and Zongozotla (41). Fieldwork teams were led by Canek Ledesma Corral and Miriam Jiménez Chimil and included Ceferino Salgado Castañeda, Mariano Gorostiza Salazar, Eleuterio Gorostiza Salazar, and Osbel López Francisco.

References

1. Almeida TE, Salino A. State of the art and perspectives on Neotropical fern and lycophyte systematics. J Syst Evol. 2016;54(6):679–690. doi: 10.1111/jse.12223
2. Kessler M. Biogeography of ferns. In: Mehltreter K, Walker LR, Sharpe JM, editors. Fern ecology. Cambridge (UK): Cambridge University Press; 2010. p. 22–60. doi:10.1017/CBO9780511844898.003
3. Suissa JS, Sundue MA, Testo WL. Mountains, climate and niche heterogeneity explain global patterns of fern diversity. J Biogeogr. 2021;48:1296–308. doi:10.1111/jbi.14076
4. Hernández-Álvarez AG, Sánchez-González A, Tejero-Díez JD. Licofitas y helechos del bosque mesófilo de montaña del estado de Hidalgo. Bot Sci. 2019;97:236–49. doi:10.17129/botsci.2093
5. Tejero-Díez JD, Torres-Díaz AN, Gual-Díaz M. Licopodios y helechos en el bosque mesófilo de montaña de México. In: Gual-Díaz M, Rendón-Correa A, editors. Bosques mesófilos de montaña de México: diversidad, ecología y manejo. México: Comisión Nacional para el Conocimiento y Uso de la Biodiversidad; 2014.
6. Tejero-Díez JD, Torres-Díaz AN, Sánchez-González A. Helechos de la Sierra Madre del Sur. In: Luna-Vega I, Espinosa D, Contreras-Medina R, editors. Biodiversidad de la Sierra Madre del Sur: una síntesis preliminar. Ciudad de México: Universidad Nacional Autónoma de México; 2016. pp. 121–55.

7. Brodribb TJ, Holbrook NM. Stomatal protection against hydraulic failure: a comparison of coexisting ferns and angiosperms. New Phytol. 2004;162(3):663–670. doi:10.1111/j.1469-8137.2004.01060.x

8. Brodribb TJ, McAdam SAM, Jordan GJ, Feild TS. Evolution of stomatal responsiveness to CO_2 and optimization of water-use efficiency among land plants. New Phytol. 2009;183(3):839–847. doi:10.1111/j.1469-8137.2009.02844.x

9. Brodribb TJ, McAdam SAM. Passive origins of stomatal control in vascular plants. Science. 2011;331(6017):582–585. doi:10.1126/science.11979

10. Silva MM, Farias RP, Costa LEN, Barros ICLA. Leaf phenological traits of the tree fern *Cyathea praecincta* (Cyatheaceae) in a Brazilian lowland tropical forest. Aust J Bot. 2018;66(8):618–27. doi:10.1071/BT18084

11. Yang J, Wang D, Liu L, Zhou Y. Fernlike plants establishing the understory of the Late Devonian Xinhang lycopsid forest. Life. 2024;14(5):602. doi:10.3390/life14050602

12. Hassler M. World ferns. Synonymic checklist and distribution of ferns and lycophytes of the world. Version 19.3. 1994–2024 [cited 2024 Jun 18].

13. Villaseñor JL. Checklist of the native vascular plants of Mexico. Revista Mexicana de Biodiversidad. 2016 Sep;87(3):559–902. doi:10.1016/j.rmb.2016.06.017.

14. Tejero-Díez JD, Contreras-Medina R, Torres-Díaz AN, González-Elizondo MS, Sánchez-González A, Luna-Vega I. Diversity of the Pteridoflora of montane northwestern Mexico. Diversity. 2023;15:324. doi:10.3390/d15030324

15. Mickel JT, Smith AR. The pteridophytes of Mexico. Mem NY Bot Gard. 2004;88:1–1054.

16. Luna-Vega I, Alcántara-Ayala O, Contreras-Medina R. Patterns of diversity, endemism and conservation: an example with Mexican species of Ternstroemiaceae Mirb. ex DC. In: Luna-Vega I, Morrone JJ, Espinosa D, editors. Biodiversidad de la Sierra Madre Oriental. Ciudad de México: Universidad Nacional Autónoma de México CONABIO; 2004. pp. 2723–2739.

17. Rzedowski J. Catálogo preliminar de plantas vasculares de distribución restringida a la Sierra Madre Oriental. Flora del Bajío y de regiones adyacentes. Fascículo complementario XXXI; 2015. 36 p.

Formatted: English (United States)

Formatted: English (United States)

18. Villaseñor JL, Ortiz E. A phytogeographic assessment of the Sierra Madre Oriental physiographic province, Mexico. Bot Sci. 2022;100(4):1102–23. doi:10.17129/botsci.3086

19. Salinas-Rodríguez MM, Hernández-Sandoval L, Carrillo-Reyes P, Castillo-Gómez HA, Castro-Castro A, Estrada-Castillón E, et al. Diversidad de plantas vasculares de la provincia fisiográfica de la Sierra Madre Oriental, México. Bot Sci. 2022;100(2):469–92. doi:10.17129/botsci.2864

20. Sanginés-Franco C, Luna-Vega I, Alcántara-Ayala O, Contreras-Medina R. Distributional patterns and biogeographic analysis of ferns in the Sierra Madre Oriental, Mexico. Am Fern J. 2011;101(2):81–104. doi:10.1640/0002-8444-101.2.81

21. Hernández-Cárdenas RA, Serrato-Díaz A, López-Ferrari AR, Espejo-Serna A. Novelties in the genus *Viridantha* Espejo (Tillandsioideae, Bromeliaceae). PhytoKeys. 2019;132:99–110.

22. Smith AR. Pteridophytes. In: Breedlove DE, editor. Flora of Chiapas. Part 2. San Francisco: California Academy of Sciences; 1981. pp. 1–370.

23. Pteridophyte Phylogeny Group (PPG I). A community-derived classification for extant lycophytes and ferns. J Syst Evol. 2016;54:563–603. doi:10.1111/jse.12229

24. Morrone JJ. Neotropical biogeography: regionalization and evolution. Boca Raton (FL): CRC Press; 2017. doi:10.1201/b21824

25. Eguiluz de Antuñano S, Aranda-García M, Marrett R. Tectónica de la Sierra Madre Oriental, México. Bol Soc Geol Mex. 2000;53:1–26.

26. INEGI. Fisiografía. Conjunto de datos vectoriales Fisiográficos. Continuo Nacional. escala 1:1 000 000. Serie I. Aguascalientes, Mexico: Instituto Nacional de Estadística y Geografía; 2001. [cited 2025 Dec] Available from: https://www.inegi.org.mx/temas/fisiografia/

27. Santa Anna del Conde H, Contreras-Medina R, Luna-Vega I. Biogeographic analysis of endemic cacti of the Sierra Madre Oriental, Mexico. Biol J Linn Soc. 2009;97(2):373–89. doi:10.1111/j.1095-8312.2009.01212.x

28. Hernández-Cerda ME, Carrasco-Anaya G. Climatología. In: Luna-Vega I, Morrone JJ, Espinosa D, editors. Biodiversidad de la Sierra Madre Oriental. Mexico City: Universidad Nacional Autónoma de México; 2004. pp. 63–108.

Formatted: English (United States)

29. Álvarez-Zúñiga E, Sánchez-González A, López-Mata L, Tejero-Díez JD. Composición y abundancia de las pteridofitas en el bosque mesófilo de montaña del municipio de Tlanchinol, Hidalgo, México. Bot Sci. 2012;90(2):163–177.
30. Andrés-García MF. Efecto de la perturbación sobre la pteridoflora de un bosque mesófilo de montaña en la Sierra Norte de Puebla [tesis de licenciatura]. Ciudad de México: FES-Iztacala, UNAM; 2022.
31. Cartujano S, Zamudio S, Alcántara O, Luna-Vega I. El bosque mesófilo de montaña en el municipio de Landa de Matamoros, Querétaro, México. Bol Soc Bot Mex. 2002;70:13–43.
32. Cerón-Carpio AB, Arreguín-Sánchez ML, Fernández-Nava R. Listado con anotaciones de las pteridofitas del municipio de Tlatlauquitepec, Puebla, México y su distribución de las especies en los diferentes tipos de vegetación. Polibotánica. 2006;21:45–60.
33. Cerón-Carpio AB, Rojas-Alvarado AF, Tejero-Díez JD, Caamaño-Onofre L. Los híbridos de *Pleopeltis* (Polypodiaceae, Polypodiophyta) en México: novedades y precisiones. Acta Bot Mex. 2017;119:101–14. doi:10.21829/abm119.2017.1234
34. Clausen RT. Ferns of the Monterrey Region, Mexico. Am Fern J. 1950;40(1):89–97. doi:10.2307/1544950
35. García-Morales LJ, Estrada-Castillón AE, García-Jiménez J, Villarreal-Quintanilla JA, Cantú-Ayala C, Jurado-Ybarra E, et al. Florística y vegetación del Área Natural Protegida Altas Cumbres, Tamaulipas, México. In: Barrientos-Lozano L, Correa Sandoval A, Horta Vega JV, García Jiménez J, editors. Biodiversidad de Tamaulipas. Vol. 2. Cd. Victoria: Dirección General de Educación Superior, Instituto Tecnológico de Ciudad Victoria; 2014.
36. Gómez-Noguez F, Mendoza-Ruiz A, Pérez-García A. Nota sobre *Onocleopsis hintonii* (Onocleaceae), un nuevo registro para el estado de Hidalgo (México). Acta Bot Mex. 2013;102:31–7. doi:10.21829/abm102.2013.23
37. Grajales-Tam KM. Azollaceae. Flora del Bajío y de regiones adyacentes, INECOL. 2014;185:1–7. doi:10.21829/fb.60.2014.185
38. Gutiérrez-Lozano M, Sánchez-González A, López-Mata L, Tejero-Díez JD. Taxonomic richness of lycophytes and ferns of the Mexican beech forest: Highest

ever recorded among *Fagus* forests worldwide? Flora. 2017;229:23–31. doi:10.1016/j.flora.2017.02.008

39. Guzmán-Cornejo L, Sánchez-Morales A, Pacheco L. Helechos y licofitas del municipio de Cardonal, Hidalgo, México. Rev Mex Biodivers. 2015;86(3):573–81. doi:10.1016/j.rmb.2015.06.004

40. Hernández-Mendoza AM, Arreguín-Sánchez ML, García-Jiménez J, Herrera-Monsivais MC. Composición taxonómica y datos ecológicos de las licofitas y monilofitas de la Sierra de Tamaulipas, Tamaulipas, México. Polibotánica. 2015;40:29–44.

41. Hernández-Rojas AC, Kluge J, Krömer T, Carvajal-Hernández C, Silva-Mijangos L, Miehe G, et al. Latitudinal patterns of species richness and range size of ferns along elevational gradients at the transition from tropics to subtropics. J Biogeogr. 2020;47(6):1383–97. doi:10.1111/jbi.13841

42. Juárez-Medina AK. Biodiversidad de la flora del bosque mesófilo de montaña del municipio de Huayacocotla, Veracruz, México [Bachelor dissertation]. Mexico City: Facultad de Ciencias, Universidad Nacional Autónoma de México; 2008.

43. Krömer T, Carvajal-Hernández CI, Acebey AR. A decade of new Pteridophyte records for the state of Veracruz, Mexico. Am Fern J. 2015;105(3):162–75.

44. Martínez-Cabrera D, Hernández-Hernández NN, Isidro-Hernández B, Hernández-Álvarez AG, Sánchez-González A. Diversidad de licopodios y helechos del bosque tropical subcaducifolio del estado de Hidalgo, México. Acta Bot Mex. 2019;126:e1434. doi:10.21829/abm126.2019.1434

45. Mendoza A, Windham M, Pérez-García B, Yatskievych G. Una nueva especie de *Pellaea* (Pteridaceae) del estado de San Luis Potosí, México. Acta Bot Mex. 2001;57:15–21. doi:10.21829/abm57.2001.881

46. Mendoza-Ruiz A, Ceja-Romero J. Nuevos registros de licofitas y helechos para el estado de Puebla, México. Acta Bot Mex. 2020;127:e1637. doi:10.21829/abm127.2020.1637

47. Moran RC, Prado J. *Megalastrum* (Dryopteridaceae) in Central America. Kew Bull. 2010;65(2):137–88. doi:10.1007/s12225-010-9214-3

Formatted: Spanish (Mexico)

48. Mora-Olivo A, Mendoza-Ruiz A, Martínez-Ávalos JG. *Isoetes tamaulipana* (Isoetaceae), a new species from Mexico. Phytotaxa. 2016;267(2):113–20. doi:10.11646/phytotaxa.267.2.3
49. Palacios-Ríos M. Las pteridofitas del estado de Veracruz, México [Master's thesis]. Mexico City: Facultad de Ciencias, Universidad Nacional Autónoma de México; 1992.
50. Pérez-Atilano Y, Sánchez-González A, Tejero-Díez JD. Species richness, distribution, and morphological variation of lycophytes and monilophytes in a semi-arid region of Mexico. Am Fern J. 2015;105(3):238–56. doi:10.1640/0002-8444-105.3.238
51. Pérez-Paredes MG, Sánchez-González A, Tejero-Díez JD. Listado de licopodios y helechos del municipio de Zacualtipán de Ángeles, Hidalgo, México. Polibotánica. 2012;33:57–73.
52. Ramírez-Cruz S, Sánchez-González A, Tejero-Díez D. La pteridoflora del Parque Nacional Los Mármoles, Hidalgo, México. Bol Soc Bot Mex. 2009;84:35–44. doi:10.17129/botsci.2293
53. Rojas-Alvarado AF. Notes on *Elaphoglossum* (Lomariopsidaceae) section *Polytrichia* subsection *Hybrida* in Mexico and Central America. Rev Biol Trop. 2003;51(1):33–48.
54. Romero-Santamaría A. Diversidad y estructura de especies leñosas en un fragmento de bosque mesófilo de montaña en Xicotepec, Puebla, México [Bachelor dissertation]. Mexico City: FES-Iztacala, Universidad Nacional Autónoma de México; 2017.
55. Sánchez-González A, Álvarez-Zúñiga E, Tejero-Díez JD. Richness and distribution patterns of ferns and lycopods in Los Mármoles National Park, Hidalgo, Mexico. J Torrey Bot Soc. 2010;137(4):373–9. doi:10.3159/10-RA-002.1
56. Tejero-Díez JD, Pacheco L. Notas taxonómicas y de distribución en *Polypodium plesiosorum* y *P. rhodopleuron* (Polypodiaceae: Pteridophyta). An Inst Biol Univ Nac Autón Méx Ser Bot. 2004;75(1):11–37.

57. Tejero-Díez JD, Pacheco L. Taxa nuevos, nomenclatura, redefinición y distribución de las especies relacionadas con *Polypodium colpodes* Kunze (Polypodiaceae: Pteridophyta). Acta Bot Mex. 2004;67:75–115. doi:10.21829/abm67.2004.976
58. Torres-Colín R, Parra JG, de la Cruz LA, Ramírez MP, Gómez-Hinostrosa C, Bárcenas RT, Hernández HM. Flora vascular del municipio de Guadalcázar y zonas adyacentes, San Luis Potosí, México. Rev Mex Biodivers. 2017;88(3):524–54. doi:10.1016/j.rmb.2017.07.003
59. Tzompa-Coatl R, Cerón-Carpio AB, Mendoza-Ruiz A, Ceja-Romero J. Riqueza específica y distribución de licofitas y helechos por tipos de vegetación, en la Sierra Norte de Puebla, México. Acta Bot Mex. 2022;129:e2063. doi:10.21829/abm129.2022.2063
60. Velazco-Macías CG, Salcedo-Martínez SM, Alanís-Flores GJ, González-Álvarez M, Alvarado-Vázquez MA. Helechos de Nuevo León. Una guía ilustrada. Monterrey: Universidad Autónoma de Nuevo León; 2012.
61. Velázquez-Montes E. Pteridaceae. Flora del Bajío y de Regiones Adyacentes, INECOL. 2019;210:1–254. doi:10.21829/fb.40.2019.210
62. Thiers B. Index Herbariorum: a global directory of public herbaria and associated staff [Internet]. New York: New York Botanical Garden's Virtual Herbarium; [cited 2024 Nov 3].
63. Environmental Systems Research Institute (Esri). ArcGIS Pro. Version 3.0.1. Redlands (CA): Esri; 2022..
64. Turland NJ, Wiersema JH, Barrie FR, Greuter W, Hawksworth DL, Herendeen, Pb et al., editors. International Code of Nomenclature for algae, fungi, and plants (Shenzhen Code) adopted by the Nineteenth International Botanical Congress Shenzhen, China, July 2017. Regnum Vegetabile 159. Glashütten: Koeltz Botanical Books; 2018. doi:10.12705/Code.2018
65. Zhou XM, Zhang LB. Phylogeny, character evolution, and classification of Selaginellaceae (lycophytes). Plant Divers. 2023;45:630–84. doi:10.1016/j.pld.2023.07.003

Formatted: English (United States)

~~65.~~66. Ortiz-Bermúdez, E. Nomenclator de localidades. In: Biodiversidad de la Sierra Madre Oriental, Luna-Vega I, Morrone JJ, Espinosa D, editors. Mexico City: UNAM-CONABIO; 2004. pp. 25-62.

~~66.~~67. Rodríguez A, Castro-Castro A, Vargas-Amado G, Vargas-Ponce O, Zamora-Tavares P, González-Gallegos J, et al. Richness, geographic distribution patterns, and areas of endemism of selected angiosperm groups in México. J Syst Evol. 2018;56(5):537–49. doi:10.1111/jse.12457

~~67.~~68. Villaseñor JL, Ortiz E, Juárez D. Transition zones and biogeographic characterization of endemism in three biogeographic provinces of central Mexico. Bot Sci. 2021;99:938–54. doi:10.17129/botsci.2768

69. Contreras-Medina R, García-Martínez AI, Ramírez-Martínez JC, Espinosa D, Balam-Narváez R, Luna-Vega I. Biogeographic analysis of ferns and lycophytes in Oaxaca: a Mexican beta-diverse area. Bot Sci. 2022;100:204–22. doi:10.17129/botsci.2844

~~68.~~70. Colwell RK. EstimateS: Statistical estimation of species richness and shared species from samples. Version 9.1. User's Guide and application published at: http://purl.oclc.org/estimates; 2009.

~~69.~~71. Ramírez-Barahona S, Luna-Vega I, Tejero-Díez JD. Species richness, endemism and conservation of American tree ferns (Cyatheales). Biodivers Conserv [Internet]. 2011;20:59–72. doi:10.1007/s10531-010-9946-2.

~~70.~~72. Sanginés-Franco C, Luna-Vega I, Contreras-Medina R, Espinosa D, Tejero-Díez JD, Rivas G. Diversity, endemism and conservation of ferns (Polypodiales) in the Mexican Mountain Component. J Mt Sci. 2015;12:891–904. doi:10.1007/s11629-014-3070-9

~~71.~~73. Crisp MD, Laffan SW, Linder HP, Monro A. Endemism in the Australian flora. J Biogeogr 2001 Feb;28(2):183–98.

~~72.~~74. Linder HP. Plant diversity and endemism in subSaharan tropical Africa. J Biogeogr 2001 Feb;28(2):169–82. doi:10.1046/j.1365-2699.2001.00527.x

~~73.~~75. Laffan SW, Lubarsky E, Rosauer DF. Biodiverse, a tool for the spatial analysis of biological and related diversity. Ecography. 2010;33(4):643–47. doi:10.1111/j.1600-0587.2010.06237.xC

Field Code Changed

Formatted: English (United States)

Formatted: Font: (Default) Times New Roman, 12 pt, Font color: Auto, English (United States), Pattern: Clear

~~74.~~76. Aagesen L, Szumik C, Goloboff P. Consensus in the search for areas of endemism. J Biogeogr. 2013;40:2011–2016. doi:10.1111/jbi.12172

~~75.~~77. Morrone JJ. Evolutionary biogeography: An integrative approach with case studies. New York: Columbia University Press; 2009. doi:10.1093/sysbio/syq016

~~76.~~78. Vaghasiya PM, Dhamsaniya S, Paradva BR, Patel RM. Vegetation characteristics of Chapredi Reserve Forest of Bhuj-Kachchh, Gujarat. J Appl For Ecol. 2015;3(1):65–73.

~~77.~~79. Squeo F, Caviers L, Arancio G, Novoa J, Matthei O, Marticorena C, et al. Biodiversidad de la flora vascular en la Región de Antofagasta, Chile. Rev Chil Hist Nat. 1998;71:571–91.

~~78.~~80. Carvajal-Hernández CI, Krömer T. Riqueza y distribución de helechos y licófitos en el gradiente altitudinal del Cofre de Perote, centro de Veracruz, México. Bot Sci. 2015;93:601–14. doi:10.17129/botsci.165

~~79.~~81. Murillo J, Murillo MT. Diversidad de los helechos y licófitos de Colombia. Acta Bot Malacit. 2017;42:23–32. doi:10.24310/abm.v42i1.2654

~~80.~~82. Salazar L, Homeier J, Kessler M, Abrahamczyk S, Lehnert M, Krömer T, Kluge J. Diversity patterns of ferns along elevational gradients in Andean tropical forests. Plant Ecol Divers. 2013;8:13–24. doi:10.1080/17550874.2013.843036

~~81.~~83. Luna-Vega I, Tejero-Díez D, Contreras-Medina R, Heads M, Rivas G. Biogeographical analysis of two *Polypodium* species complexes (Polypodiaceae) in Mexico and Central America. Biol J Linn Soc. 2012;106:940–55. doi:10.1111/j.1095-8312.2012.01921.x

~~82.~~84. Salinas-Moreno Y, Mendoza MG, Barrios MA, Cisneros R, Macías-Sámano J, Zúñiga G. Areography of the genus *Dendroctonus* (Coleoptera: Curculionidae: Scolytinae) in Mexico. J Biogeogr. 2004;31:1163–77. doi:10.1111/j.1365-2699.2004.01110.x

~~83.~~85. SEMARNAT. Modificación del Anexo Normativo III, Lista de especies en riesgo de la Norma Oficial Mexicana NOM-059-SEMARNAT-2010. Protección ambiental-Especies nativas de México de flora y fauna silvestres-Categorías de riesgo y especificaciones para su inclusión, exclusión o cambio-Lista de especies en riesgo. DOF. 2019. https://www.dof.gob.mx/nota_detalle_popup.php?codigo=5578808

~~84.~~86. IUCN. 2025. The IUCN Red List of Threatened Species. Version 2025-1. Accessed on [18 June 2025]. https://www.iucnredlist.org.

87. CITES. Convention on International Trade in Endangered Species of Wild Fauna and Flora. Appendices I, II and III. 2025. Available from: https://cites.org/eng/app/appendices.php

~~85.~~88. Sánchez-Salas J, Muro G, Estrada-Castillón E, Alba-Ávila JA. El MER: un instrumento para evaluar el riesgo de extinción de especies en México. Rev Chapingo Ser Zonas Áridas. 2013;12:30–5. doi:10.5154/r.rchsza.2012.06.037

89. Chao A. Non-parametric estimation of the number of classes in a population. Scand J Stat 1984; 11: 265–270. https://doi.org/10.2307/4615964

90. Chao A. Estimating the population size for capture-recapture data with unequal catchability. Biometrics 1987;43: 783–791. https://doi.org/10.2307/2531532

~~86.~~91. Villarreal H, Álvarez M, Córdoba S, Escobar F. Manual de métodos para el desarrollo de inventarios de biodiversidad. Programa de Inventarios de Biodiversidad. Bogotá, Colombia: Instituto de Investigación de Recursos Biológicos Alexander von Humboldt. 2006.

~~87.~~92. Hernández-Rojas AC, Kluge J, Noben S, Reyes-Chávez JD, Krömer T, Carvajal-Hernández CI, et al. Phylogenetic diversity of ferns reveals different patterns of niche conservatism and habitat filtering between epiphytic and terrestrial assemblages. Front Biogeogr. 2021;13(3). doi:10.21425/F5FBG50023

~~88.~~93. Suissa JS, Sundue MA. Diversity patterns of Neotropical ferns: revisiting Tryon’s centers of richness and endemism. Am Fern J. 2020;110:211–32. doi:10.1640/0002-8444-110.4.211

~~89.~~94. Grajales-Tam KM, Tejero-Díez JD. Pteridaceae I: Subfamilia Cryptogrammoideae. Flora de Veracruz. 2024; fasc. 24. doi:10.21829/fv.620.2024.204

~~90.~~95. Sundue MA, Testo WL, Ranker TA. Morphological innovation, ecological opportunity, and the radiation of a major vascular epiphyte lineage. Evolution. 2015;69:2482–95. doi:10.1111/evo.12749

~~91.~~96. Sessa ES, Zimmer EA, Givnish TJ. Phylogeny, divergence times, and historical biogeography of New World *Dryopteris* (Dryopteridaceae). Am J Bot. 2012;99:730–50. doi:10.3732/ajb.1100294

Field Code Changed

~~92.~~97. Rouhan G, Dubuisson JY, Rakotondrainibe F, Motley TJ, Mickel JT, Labat JN, Moran RC. Molecular phylogeny of the fern genus *Elaphoglossum* (Elaphoglossaceae) based on chloroplast non-coding DNA sequences: contributions of species from the Indian Ocean area. Mol Phylogenet Evol. 2004;33(3):745–63. doi:10.1016/j.ympev.2004.08.006

~~93.~~98. Alejo-Jacuinde G, Kean-Galeno T, Martínez-Gallardo N, Tejero-Díez D, Mehltreter K, Délano-Frier JP, et al. Viability markers for determination of desiccation tolerance and critical stages during dehydration in *Selaginella* species. J Exp Bot. 2022;73(12):3898–3912. doi:10.1093/jxb/erac121

~~94.~~99. Korall P, Kenrick P. Phylogenetic relationships in Selaginellaceae based on Rbcl sequences. Am J Bot. 2002;89(3):506–17. doi:10.3732/ajb.89.3.506. PMID: 21665649

~~95.~~100. Schneider EL, Carlquist S. SEM studies on the vessels of heterophyllous species of *Selaginella*. J Torrey Bot Soc. 2000;127(4):263–70. doi:10.2307/3088644

101. Morrone JJ. The Mexican Transition Zone: A natural biogeographic laboratory to study biotic assembly. Cham: Springer; 2020. doi:10.1007/978-3-030-47917-6

102. James SA, Soltis PS, Belbin L, Chapman AD, Nelson G, Paul DL, Collins M. Herbarium data: Global biodiversity and societal botanical needs for novel research. Appl. Plant Sci. 2018;6(2):e1024. doi:10.1002/aps3.1024

103. Daru BH, Rodriguez J. Mass production of unvouchered records fails to represent global biodiversity patterns. Nat Ecol Evol, 2023;7:816-831. doi:10.1038/s41559-023-02047-3

104. Daru BH. Tracking hidden dimensions of plant biogeography from herbaria. New Phytol. 2025;246(1):61-77. doi:10.1111/nph.70002

~~96.~~105. Tejero-Díez JD, Torres-Díaz AN, Mickel JT, Mehltreter K, Krömer T. Helechos y licopodios. In: Comisión Nacional para el Conocimiento y Uso de la Biodiversidad (CONABIO). La biodiversidad en Veracruz: estudio de estado. México: CONABIO, Gobierno del Estado de Veracruz, Universidad Veracruzana, Instituto de Ecología A.C. 2011. Vol. II. p. 97–115 + apéndice VIII.6.

~~97.~~106. Armenta-Montero S, Carvajal-Hernández CI, Ellis EA, Krömer T. Distribution and conservation status of *Phlegmariurus* (Lycopodiaceae) in the state of

Formatted: Font: (Default) Times New Roman, 12 pt, Font color: Auto, English (United States), Pattern: Clear

Veracruz, Mexico. Trop Conserv Sci. 2015;8:114–137. doi:10.1177/194008291500800

~~98.~~107. Dadang RJ, Casinillo NG, Coritico FP, Simborio LT, Amoroso VB. Living with endangered species: collection of tree ferns in the forest reserve of Marilog district, southern Philippines. Trees For Peop. 2020;2:100041. doi: 10.1016/j.tfp.2020.100041

~~99.~~108. Luna-Vega I, Alcántara-Ayala O, Contreras-Medina R, Ponce-Vargas A. Biogeography, current knowledge and conservation of threatened vascular plants characteristic of Mexican temperate forests. Biodivers Conserv. 2006;15(12):3773–99. doi:10.1007/s10531-005-5401-1

~~100.~~109. Luna-Vega I, Alcántara-Ayala O, García-Morales LJ, Espinosa D, Ramírez-Martínez JC, Contreras-Medina R. Threatened trees characteristic of Mexican tropical montane cloud forests. Diversity. 2023;15(1):42. doi:10.3390/d15010042

~~101.~~110. Eleutério AA, Pérez-Salicrup D. Management of tree ferns (*Cyathea* spp.) for handicraft production in Cuetzalan, Mexico. Econ Bot. 2006;60(2):182–6. doi:0.1663/0013-0001(2006)60[182:MOTFCS]2.0.CO;2

~~102.~~111. Tejero-Díez J D, Torres-Díaz AN. *Phymatosorus grossus* (Polypodiaceae) en México y comentarios sobre otros pteridobiontes no-nativos. Acta Bot Mex. 2012;98:111–124. doi:10.21829/abm98.2012.1143

Departamento de Biología Evolutiva

Facultad de Ciencias ▲ Universidad Nacional Autónoma de México

February 03, 2026.

Dr. Marcela Pagano

Academic Editor

PLOS ONE

Dear Dr. Pagano:

With this letter, we acknowledge the opportunity to revise and resubmit our manuscript PONE-D-25-44575 entitled: "Richness and biogeography of Pteridoflora in montane forests of eastern Mexico" to be published in the journal PLOS ONE. We appreciate the valuable comments, suggestions, and positive feedback from the reviewers, which have significantly enhanced our manuscript.

We thoroughly revised the manuscript, modifying two figures and adding another, and incorporated the reviewers' suggestions. We described and adjusted the methodological framework to address the reviewers' comments. These revisions aim to improve clarity, rigor, and relevance, ensuring our manuscript meets expectations for understanding the distribution patterns of the pteridoflora in the Sierra Madre Oriental, one of Mexico's main mountain chains. Enclosed in the subsequent pages is our detailed response to each comment and suggestion made by the reviewers.

In this revised submission, we have specifically addressed the copyright queries regarding the map figures (Figures 1, 2, 5, and 7). To ensure strict compliance with PLOS ONE's CC BY 4.0 license, we have replaced all previous vector layers with open-access data from Mexico's National Institute of Statistics and Geography (INEGI). These datasets operate under terms that explicitly permit commercial exploitation, effectively resolving the previous licensing conflicts.

We believe that the revisions have addressed the concerns raised during the review process, and we are confident that the updated manuscript reflects the improvements recommended by the two reviewers.

Finally, we appreciate your time and consideration. We look forward to the opportunity to discuss any further adjustments or clarifications that may be required.

Regards,

Dra. Isolda Luna-Vega

Corresponding author

Apartado postal 70-399, México 04510, D. F. Tel: 5622-4827. Fax: 5622-4828

Rebuttal

Dear Editor,

In response to your request regarding PONE-D-25-44575R1:

Thank you for notifying us that the new files were not received. We suspect a technical synchronization issue occurred because the previous filenames remained identical to the original submission.

To resolve this and ensure the correct versions are processed, we have re-uploaded the revised Figures 1, 2, 5, and 7 with distinct filenames (labeled as 'Figure 1 new', 'Figure 2 new', etc.) to distinguish them from the previous versions.

We explicitly confirm that these specific files (uploaded on Feb 09, 2026) are the revised versions. We have double-checked the file inventory to ensure that these images reflect the changes described in our rebuttal letter: they have been reconstructed using exclusively open-access vector layers from INEGI (Fisiografía, Vegetation Series VII, Hypsometry, and Geostatistical Framework), compliant with the CC BY 4.0 license.

The proprietary or restricted layers (National Atlas /CONABIO) have been completely removed from these files.

Thank you for your guidance regarding data licensing. We understand the importance of strict compliance with PLOS ONE's CC BY 4.0 license, particularly regarding unrestricted commercial reuse.

Instead, all geographical information in the revised figures is now sourced exclusively from INEGI (National Institute of Statistics and Geography of Mexico), whose "Open Data" policy explicitly permits commercial exploitation.

1. Verification of Datasets Used (All sourced from INEGI). As requested, here are the exact datasets and direct URLs for the layers used in the revised maps:

- Physiographic Regionalization (SMOR): *Conjunto de datos vectoriales Fisiográficos. Continuo Nacional.*
    - URL: https://www.inegi.org.mx/temas/fisiografia/
- Land Use and Vegetation (Series VII): *Conjunto de datos vectoriales de la carta de Uso del Suelo y Vegetación. Serie VII.*
    - URL: https://www.inegi.org.mx/temas/usosuelo/
- Elevation/Hypsometry: *Conjunto de datos vectoriales de información topográfica (Contour).*

    - URL: https://www.inegi.org.mx/temas/topografia/
- Political Division: *Marco Geoestadístico (National Geostatistical Framework).*
    - URL: https://www.inegi.org.mx/temas/mg/

2. License Compatibility (Proof of Commercial Use) All the datasets listed above are governed by INEGI's "Terms of Free Use of Information" (*Términos de Libre Uso de la Información*).

- License Terms URL: https://www.inegi.org.mx/inegi/terminos.html

These terms are fully compatible with CC BY 4.0. Under Section II of the agreement, INEGI explicitly grants the user permission to:

1. *"Make and distribute copies of the information."*
2. *"Adapt and reorder the information."*
3. "Commercially exploit the information, using it as input to generate other products or services."

We have updated the figure captions in the manuscript to attribute INEGI as the sole source of the vector data, citing the "Terms of Free Use" to ensure clarity for future readers.

We confirm that these figures are now fully compliant with the journal's Open Science and copyright policies.

Reviewer #1:

I have reviewed your manuscript titled "Richness and biogeography of Pteridoflora in montane forests of eastern Mexico" and found it to be well-written. The methods are appropriate, and the results and discussion are well presented. I have only two concerns:

1. You need to explicitly explain how you obtained the geographical coordinates for the specimens that lack them.

Author's response: For herbarium specimens without geographic coordinates, we used topographic maps at scale 1:50,000 produced by the Instituto Nacional de Estadística y Geografía (INEGI) and the previously published nomenclator of localities for the Sierra Madre Oriental (Ortiz-Bermúdez, 2004) to obtain the corresponding geographic coordinates. At the end, we tried to include only georeferenced and verified data in our study.

Ortiz-Bermúdez, E. (2004). Nomenclator de localidades. In: Biodiversidad de la Sierra Madre Oriental, Luna-Vega I., J.J. Morrone & D. Espinosa (eds.). pp. 25-62. Mexico City: UNAM-CONABIO.

2. The dataset used for all the analyses (species with their geographical coordinates) is not available. I encourage you to make this information public.

Author's response: The database is available through personal communication to the first author.

Reviewer #2: Methodologically, I think the article is well presented. Although it is presented from a descriptive perspective of biodiversity, I believe that further exploration is needed to evaluate the reasons behind the results.

Spatial sampling bias:

1.The manuscript integrates data from literature, herbaria, and field collections; however, the potential spatial bias of sampling effort is not evaluated. It is recommended to include a sampling density map to visualize the spatial distribution of records.

Author's response: We have already created a sampling density map to show the spatial distribution of records. We chose not to include it in the first draft because of the manuscript's length. In this new version, we are adding the suggested map to Figure 2.

Reviewer 2: The referee suggested that the use of spatial rarefaction methods or effort-weighted corrections could improve the interpretation of results.

Author's response: As suggested by the referee, we undertook an effort-weighted sampling method for our manuscript. In our study using the Chao1 richness estimator, we identified 567 species. According to the estimator, the average estimated richness at this sampling level was about 670 species, with a 95% confidence interval of approximately 632-729 species. The difference between the lower and upper confidence limits is roughly 100 species, indicating moderate uncertainty around the estimate, as expected in communities with many rare or low-detectability taxa.

Comparing the observed richness (567 species) to the Chao1 mean estimate (≈670 species), our data falls short by about 100 species from the estimated total richness. This indicates that our sampling completeness is approximately 85%, which is fairly high, because Chao1 uses the number of very infrequently observed species to infer unseen diversity, it tends to produce higher richness estimates when rare species are relatively common in the sample (Chao, 1984; Chao, 1987).

Overall, these results suggest that although the sampling did not fully capture the total estimated richness, it still documented a significant portion of the community. To finish the task, we decided to create a new figure to compare our results with the Chao1 richness estimator (Fig. 6).

2. Details of sampling design:
Although field collections are mentioned, the manuscript does not provide information on sampling effort, the number of sites, or the spatial design employed. Providing these details would allow assessment of the representativeness of records and their potential influence on spatial patterns.

Author's response: Our study primarily relies on herbarium data and databases, so our field collection was minimal. Our database has been compiled and curated continuously by a Mexican specialist in ferns and lycophytes for over three decades. Therefore, we can confidently state that we have sufficient information on the spatial patterns of these taxa (see paragraphs above).

3. Data quality and geographic precision:
There is no evaluation of geographic precision or data quality. The manuscript does not describe how duplicate records, uncertain georeferences, or historical localities were handled. Ensuring data quality is essential to confirm that the generated maps reflect biological patterns rather than artifacts of incomplete or imprecise data.

Author's response: For vascular plants, species occurrence data typically come from direct field observations or natural history specimens in herbaria (Daru, 2025), although specimens trump observations in representing global plant biogeographic patterns (Daru & Rodriguez, 2023). In general, no hard-and-fast rule exists for judging data quality in biogeographical analyses (Zizka et al. 2019). Indeed, collecting efforts are not randomly or regularly distributed, and biodiversity patterns are scale-dependent and sensitive to spatial resolution (James et al. 2018). Also, sampling designs were rarely quantified (Daru et al. 2018). In species occurrence records, problems with geographic location are a significant concern; particularly, erroneous or

overly imprecise coordinates can bias biodiversity patterns at multiple spatial scales (Zizka et al. 2019).

As a result, data quality in online databases is a significant concern and has restricted their usefulness and reliability for research and conservation (Anderson et al. 2016). Herbarium specimens and their data have advantages that are verifiable, repeatable, sustainable, and persistent (James et al. 2018). In our paper, fortunately, most records in our database come from herbarium specimens revised directly by some of the authors, mainly the first author, a Mexican specialist in ferns and lycophytes. However, botanists have identified multiple biases in plant sampling from herbarium collections. For example, various analyses based on millions of herbarium and museum records have found spatial, temporal, taxonomic, functional trait, phylogenetic, and collector biases (Daru et al. 2018; Daru & Rodriguez 2023; Daru 2025). Limitations in our database were addressed and revised to minimize, primarily, spatial bias.

In our database, as is common with herbarium specimens, duplicate samples are found across different botanical collections. In our study, we removed duplicate records from the biogeographic analysis to ensure unique locations. Additionally, we identified and corrected records with switched latitude and longitude. We also excluded specimens with vague localities and inconsistencies. For herbarium specimens lacking geographic coordinates, we used topographic maps at a 1:50,000 scale from the Instituto Nacional de Estadística y Geografía (INEGI) and the previously published locality list for the Sierra Madre Oriental (Ortiz-Bermúdez, 2004) to obtain the corresponding coordinates. Ultimately, we aimed to include only georeferenced and verified data in our study.

Specimens stored in Natural History collections are available for long periods, sometimes up to 100 years, as in our study, because we have specimens collected in the 1880s. Unfortunately, collecting efforts are not evenly distributed over time and have declined since the mid-20th century (Daru et al. 2018; James et al. 2018). However, working with historical data presents challenges if there is no information about the species' locality or date. Additionally, the spatial location of the find may only be roughly known, or there could be issues with taxonomic naming (Lütolf et al. 2006). We examined all these issues in the historical records of ferns and lycophytes from the SMOR, and because of this, we did not include the 31 historical records from the 1800s.

Lütolf et al. (2006) highlight the use and importance of historical records, named 'ghost of past presence data', to support presence data in biogeographic studies. These records can be used as a strategy to verify a species' presence and reconfirm its presence where it was once observed. Also, these historical records are important because they can reveal changes

in species distributions driven by anthropogenic activities or climate change (James et al., 2018; Franklin, 2023).

Anderson et al. (2016). Final report of the task group of GBIF data fitness for use in distribution modelling – Are species occurrence data in global online repositories fit for modelling species distributions? The case of the Global Biodiversity Information Facility (GBIF).

Daru, B. H. (2025). Tracking hidden dimensions of plant biogeography from herbaria. New Phytologist, 246(1), 61-77.

Daru, B. H., Rodriguez, J. (2023). Mass production of unvouchered records fails to represent global biodiversity patterns. Nature Ecology & Evolution, 7, 816-831.

Daru, B. H. et al. (2018). Widespread sampling biases in herbaria revealed from large-scale digitization. New Phytologist, 217, 939-955.

Franklin, J. (2023). Species distribution modelling supports the study of past, present and future biogeographies. Journal of Biogeography, 50, 1533-1545.

James et al. (2018). Herbarium data: Global biodiversity and societal botanical needs for novel research. Applications in Plant Science, 6(2), e1024.

Lütolf et al. (2006). The ghost of past species occurrence: improving species distribution models for presence-only data. Journal of Applied Ecology, 43, 802-815.

Ortiz-Bermúdez, E. (2004). Nomenclátor de localidades. In: Biodiversidad de la Sierra Madre Oriental, Luna-Vega I., J.J. Morrone & D. Espinosa (eds.). pp. 25-62. Mexico City: UNAM-CONABIO.

Zizka et al. (2019). CoordinateCleaner: Standardized cleaning of occurrence records from biological collections databases. Methods in Ecology and Evolution, 10, 744-751.

4. Interpretation of maps and absence of records:
The manuscript assumes that the generated maps reflect biological patterns; however, it does not discuss whether absence of records corresponds to true species absence. It is suggested to clarify that some areas with no records may result from limited sampling and to consider the potential effect of this on interpreting richness and endemism hotspots.

Author's response: Fortunately, the first author of this study has been working with these organisms for more than 30 years. However, Dr. Tejero has not collected samples from all sites, so in some areas, the sampling of certain species may be incomplete.

'Presence-only' data are common sources of species distribution information, available through natural history collections like museum and herbarium collections (Lütolf et al. 2006). Absence data are rare or not easily accessible within herbarium datasets, and the absence of collections of a taxon at a specific location and time does not necessarily indicate its absence (James et al. 2018).

Mexico is a megadiverse country and, among ferns and lycophytes, is recognized as one of the Latin American countries with the highest diversity of these two plant groups (Almeida and Salino 2016). Large parts of the Neotropics remain inadequately sampled for vascular plants. The collections in this region are unevenly distributed, and many areas lack records of ferns and lycophytes (Almeida and Salino 2016). Mexico is no exception, and several regions, including the Sierra Madre Oriental, are still poorly sampled for these plants (Contreras-Medina et al. 2022). In our paper, we acknowledge this situation and state that "Some areas within the SMOR boundaries lack records of ferns or lycophytes, and 13 grid cells contain only one species. These low-diversity areas may be related to unexplored zones and insufficient sampling, as observed in many sectors of Mexico [68, 66] and the Neotropics [1, 104]."

5. Sensitivity to spatial scale:
The manuscript states that the 30′ × 30′ resolution provides reliable results, but no spatial sensitivity analysis is presented. It is recommended to recalculate richness, WE, and CWE metrics at different resolutions (e.g., 15′ and 60′) and compare patterns using rank correlations and hotspot overlap. This would allow evaluation of whether the identified richness and endemism centers are robust or dependent on the selected scale.

Author's response: In the original version, we explained the reason for choosing the 30 × 30 minutes latitude/longitude grid-cells (see page 9). "The spatial size of grid cells allows for a reliable spatial resolution of the distribution compared with other studies conducted with plants in the SMOR [18, 20, 27] regarding biodiversity data and biological conservation. We selected this scale because similar scales have been tested in previous studies on biogeography and the diversity of various biological groups in the Mexican biota [Rodríguez et al. 2018, Villaseñor et al, 2021], including ferns and lycophytes [Contreras-Medina et al., 2022]."

A 1° × 1° grid cell spatial scale has been previously used in biogeographic analyses in the SMOR, such as in the studies by Santa Anna del Conde et al.

(2009) with Cactaceae and Villaseñor & Ortiz (2022) with vascular plants. We chose a finer spatial scale to obtain more precise patterns of fern and lycophyte distribution. In biogeographic analyses at the Mexican state level, a finer scale than ours has been applied in Hidalgo (Villaseñor et al. 2022) and Oaxaca (Contreras-Medina et al. 2022), using 15 × 15-minute and 20 × 20-minute grid cells, respectively.

We consider it unnecessary to recalculate the grid-cell richness at 15 and 60 degrees, nor the endemism centers. During the development of this study, we tested different grid cell sizes, as the referee suggested, but the 30x30 size seems most suitable because many spatial studies with other Mexican taxa use this size, which is appropriate for comparative results [Rodríguez et al. 2018, Villaseñor et al., 2021].

Villaseñor JL, Ortiz E. (2022). A phytogeographic assessment of the Sierra Madre Oriental physiographic province, Mexico. Botanical Sciences, 100(4), 1102–23.

Villaseñor JL, Ortiz E, Sánchez-González A. (2022). Riqueza y distribución de la flora vascular del estado de Hidalgo, México. Revista Mexicana de Biodiversidad, 93, e933920.

Santa Anna del Conde H, Contreras-Medina R, Luna-Vega I. (2009). Biogeographic analysis of endemic cacti of the Sierra Madre Oriental, Mexico. Biological Journal of the Linnean Society, 97(2), 373–89.

6. Conclusions

The article lacks proposals for the management, conservation, and restoration of vegetation and species in the study area. I believe that this document could serve as the basis for a series of public policies for the protection of the ecosystem.

Author's response: Indeed, we do not propose in this manuscript ideas about the management, conservation, and restoration of the vegetation of this mountain range. This task involves a different methodology than the one we are not using here. Our current objective is to know the spatial distribution of the taxa. Despite this, we have elaborated some maps of Mexican Natural protected areas that will be part of a subsequent study.
