# Supplementary material for: Richness and biogeography of Pteridoflora in montane forests of eastern Mexico
Source: PLoS One. 2026 May 7;21(5):e0346965. doi: 10.1371/journal.pone.0346965 (PMC13152183; doi:10.1371/journal.pone.0346965)
Supplement: Attachment — Submitted filename: PONE review.docx [file pone.0346965.s002.docx]

1. The study presents the results of original research. Yes
2. Results reported have not been published elsewhere. No
3. Experiments, statistics, and other analyses are performed to a high technical standard and are described in sufficient detail. Yes
4. Conclusions are presented in an appropriate fashion and are supported by the data. Yes
5. The article is presented in an intelligible fashion and is written in standard English. Yes
6. The research meets all applicable standards for the ethics of experimentation and research integrity. Yes
7. The article adheres to appropriate reporting guidelines and community standards for data availability. Yes

Methodologically, I think the article is well presented.

Although it is presented from a descriptive perspective of biodiversity, I believe that further exploration is needed to evaluate the reasons behind the results.

Spatial sampling bias:

The manuscript integrates data from literature, herbaria, and field collections; however, the potential spatial bias of sampling effort is not evaluated. It is recommended to include a sampling density map to visualize the spatial distribution of records and to explicitly discuss how such bias could influence observed richness and endemism patterns. Additionally, the use of spatial rarefaction methods or effort-weighted corrections could improve the interpretation of results.

Details of sampling design:

Although field collections are mentioned, the manuscript does not provide information on sampling effort, the number of sites, or the spatial design employed. Providing these details would allow assessment of the representativeness of records and their potential influence on spatial patterns.

Data quality and geographic precision:

There is no evaluation of geographic precision or data quality. The manuscript does not describe how duplicate records, uncertain georeferences, or historical localities were handled. Ensuring data quality is essential to confirm that the generated maps reflect biological patterns rather than artifacts of incomplete or imprecise data.

Interpretation of maps and absence of records:

The manuscript assumes that the generated maps reflect biological patterns; however, it does not discuss whether absence of records corresponds to true species absence. It is suggested to clarify that some areas with no records may result from limited sampling and to consider the potential effect of this on interpreting richness and endemism hotspots.

Sensitivity to spatial scale:

The manuscript states that the 30′ × 30′ resolution provides reliable results, but no spatial sensitivity analysis is presented. It is recommended to recalculate richness, WE, and CWE metrics at different resolutions (e.g., 15′ and 60′) and compare patterns using rank correlations and hotspot overlap. This would allow evaluation of whether the identified richness and endemism centers are robust or dependent on the selected scale.

Conclusions

The article lacks proposals for the management, conservation, and restoration of vegetation and species in the study area. I believe that this document could serve as the basis for a series of public policies for the protection of the ecosystem.
